# *Leptospira interrogans* biofilm transcriptome highlights adaption to starvation and general stress while maintaining virulence

**DOI:** 10.1038/s41522-024-00570-0

**Published:** 2024-09-30

**Authors:** Grégoire Davignon, Natalia Pietrosemoli, Nadia Benaroudj, Marie-Estelle Soupé-Gilbert, Julie Cagliero, Élodie Turc, Mathieu Picardeau, Linda Guentas, Cyrille Goarant, Roman Thibeaux

**Affiliations:** 1 Leptospirosis Research and Expertise Unit, Institut Pasteur de Nouvelle-Calédonie, Institut Pasteur International Network, Nouméa, New Caledonia; 2 Exact and Applied Sciences Institute (ISEA), University of New Caledonia, BP R4, 98851 Nouméa, New Caledonia; 3 Institut Pasteur, Université Paris Cité, Bioinformatics and Biostatistics Hub, F-75015 Paris, France; 4 Biology of Spirochetes, Institut Pasteur, Université Paris Cité, CNRS UMR 6047, F-75015 Paris, France; 5 Institut Pasteur, Université Paris Cité, Plate-forme Technologique Biomics, F-75015 Paris, France; 6 Pacific Community SPC – Public Health Division – B.P. D5, Nouméa, New Caledonia

**Keywords:** Biofilms, Soil microbiology, Next-generation sequencing, Pathogens

## Abstract

Life-threatening *Leptospira interrogans* navigate a dual existence: surviving in the environment and infecting mammalian hosts. Biofilm formation is presumably an important survival strategy to achieve this process. Understanding the relation between biofilm and virulence might improve our comprehension of leptospirosis epidemiology. Our study focused on elucidating *Leptospira*’s adaptations and regulations involved in such complex microenvironments. To determine the transcriptional profile of *Leptospira* in biofilm, we compared the transcriptomes in late biofilms and in exponential planktonic cultures. While genes for motility, energy production, and metabolism were downregulated, those governing general stress response, defense against metal stress, and redox homeostasis showed a significant upsurge, hinting at a tailored defensive strategy against stress. Further, despite a reduced metabolic state, biofilm disruption swiftly restored metabolic activity. Crucially, bacteria in late biofilms or resulting from biofilm disruption retained virulence in an animal model. In summary, our study highlights *Leptospira’s* adaptive equilibrium in biofilms: minimizing energy expenditure, potentially aiding in withstanding stresses while maintaining pathogenicity. These insights are important for explaining the survival strategies of *Leptospira*, revealing that a biofilm lifestyle may confer an advantage in maintaining virulence, an understanding essential for managing leptospirosis across both environmental and mammalian reservoirs.

## Introduction

Leptospirosis is a worldwide re-emerging zoonosis with a particularly high occurrence in Oceania and Asia. This environment-transmitted infectious disease is responsible for over 1 million Human cases and 60,000 deaths annually^1^. This disease is caused by aerobic virulent bacteria of the genus *Leptospira* that can escape host immune system and induce manifestations ranging from chronic asymptomatic infection to acute infection with severe organ damage^2^.

Asymptomatic chronic carriers of pathogenic *Leptospira*, such as rodents, excrete the bacteria into the environment *via* their urine throughout their lives. Once in the soil, pathogenic bacteria can survive for several weeks and might even be able to multiply^3–5^. Rain and other extreme weather events are considered primary risk factors for infection as cases of leptospirosis increase after hurricanes or floods when people may have to wade through contaminated water^6^. Deciphering factors that promote the environmental survival and transmission of virulent *Leptospira* species has recently attracted renewed interest, driven by the persistence of these bacteria in soil and the large dominance of indirect contaminations from an environmental source in human cases.

One common adaptive strategy developed by environmental bacteria in response to extreme conditions is the production of a protective biofilm structure^7–9^ along with general stress response adaptation at the cellular level.

Biofilms are organized multicellular bacterial microcolonies embedded in a three-dimensional self-produced matrix that favors persistence and survival. This extracellular matrix is often composed of extracellular polymeric substances (EPS), proteins, extracellular DNA (exDNA), cell lysis products and materials from the surrounding environment. EPS components and, to a greater extent, exDNA are present in the *Leptospira* biofilm matrix^10^. Within the genus *Leptospira*, both saprophytes and pathogenic species are able to form a biofilm in vitro, and evidence of *Leptospira* in environmental biofilms or in vivo in animals such as rodents or horses has also been reported^9,11**–**13^.

The infectivity of pathogenic *Leptospira* in the environment is ambiguous as biofilm-forming bacteria are supposed to be less infectious or in a physiological state that is not compatible with effective infection^14–16^. On the other hand, infective dose has been proposed to be a major parameter for human environmental contamination^17^ and biofilm formation is believed to be necessary to maintain *Leptospira* sufficiently concentrated to infect a new host^18^. Therefore, *Leptospira* biofilm could not only allow persistence of the bacteria in the environment but also increase the efficiency of its transmission to mammals^19^.

In order to identify the molecular determinants and the regulatory network involved in biofilm formation and adaptation of the pathogenic *L. interrogans* to long-term persistence, we have conducted an RNA-seq analysis comparing 21 days biofilms to exponential cultures (5 days old). Our analysis revealed a shift in cellular pathways, marked by the downregulation of genes involved in motility, energy production, and metabolism. In contrast, genes implicated in protein quality control (chaperones and ATP-dependent proteolytic complexes), defense against metal stress, and redox balance were significantly upregulated, indicating a tailored protective adaptation to general cellular stress in late-stage biofilms. Interestingly, the most upregulated locus, encompassing *csoR*, *copZ*, and *copA*, was shown to be involved in the copper toxicity response in other bacteria. This suggests a possible metal ion-based stress response in biofilm environments. Additionally, we examined whether prolonged culture leading to late biofilm compromises *Leptospira* virulence. Virulence assessments in a hamster model of infection demonstrated that bacteria from both mature and disrupted biofilms had retained their pathogenicity, suggesting that physiological changes of the bacteria during biofilm maturation does not impede *Leptospira* infectivity.

By investigating global gene expression profiles of *L. interrogans* in advanced stationary phase within biofilms, we gain valuable understanding into how this pathogen adjusts its metabolic program in response to unfavorable starving conditions while maintaining its virulence. These insights contribute significantly to understanding the survival strategies and pathogenic potential of *Leptospira* in biofilms, enhancing our knowledge of its environmental persistence and transmission dynamics.

## Results

### *L. interrogans* biofilm formation and evolution

**Fig. 1:**
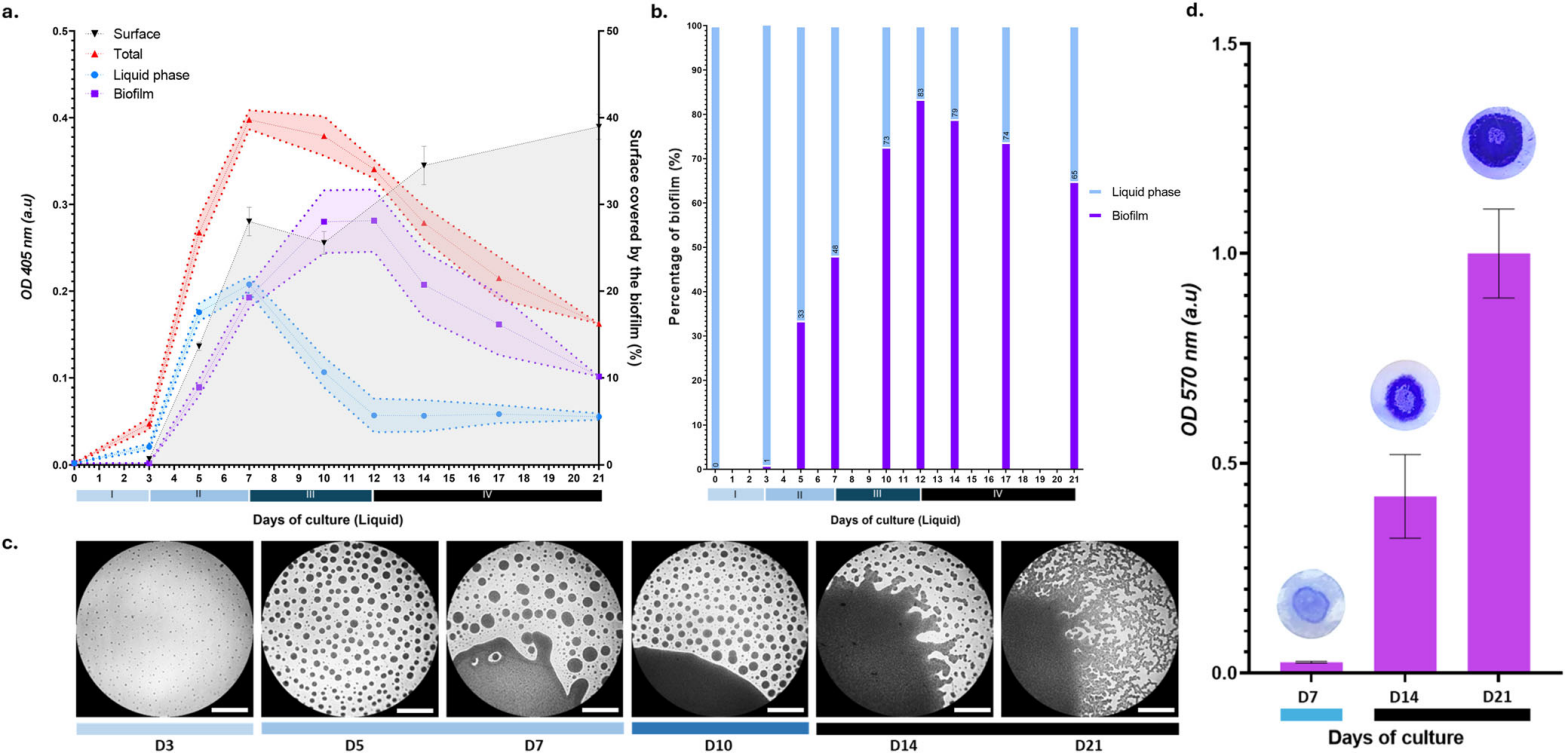
Kinetic and dynamics of *L. interrogans* L495 biofilm development over 21 days. **a** Bacterial population dynamics over 21 days, depicted as planktonic (blue), biofilm-associated (purple), and total populations (red). Error bars represent the standard error of the mean. Identified growth phases I-IV are marked by Roman numerals. Phase-contrast image analysis of biofilm coverage is shown by the gray shaded area, plotted on the secondary *y*-axis. **b** Proportion of bacteria, expressed as a percentage of the total density at each time point, represented by bar graphs for planktonic (blue, liquid phase) and biofilm-forming (purple, biofilm) bacteria. **c** Representative phase-contrast images of biofilm development at days 3, 5, 7, 10, 14, and 21 post-inoculation, illustrating progressive biofilm expansion. Scale bar = 400 μm. **d** Quantification of biofilm biomass using crystal violet staining, with absorbance measured at 570 nm and presented in arbitrary units (a.u.). Error bars depict standard deviation (SD). Insets feature corresponding filter images before crystal violet solubilization at indicated time points.

To elucidate the dynamics of *L. interrogans* biofilm formation, we quantified planktonic and biofilm-associated *Leptospira* across a 21-day period. During the initial three days (phase I), the majority of leptospires remained planktonic, predominantly motile in the liquid phase. Biofilm-associated *Leptospira* were scarcely detectable (Fig. 1a–c). Both biofilm- and liquid phase-associated bacteria exhibited exponential growth between days 3 and 7 (phase II), reaching bacterial density equivalent to 9 × 10^8^ bacteria *per* mL (OD_405_ of 0.18 and 0.21 respectively) (Fig. 1a). Simultaneously, large bacterial colonies formed, eventually coalescing into a unified, extensive biofilm structure (Fig. 1c). During this phase, the area of this structure was directly proportional to the optical density of the bacterial culture. Then, from day 7 to day 12 (phase III), the planktonic population exhibited a significant reduction, decreasing from 9 × 10^8^ to 2.5 × 10^8^ bacteria *per* mL (OD_405_ of 0.18 to 0.06). In contrast, biofilm-associated bacteria proliferated, peaking at an OD_405_ of 0.28 (1.2 × 10^9^ bacteria *per* mL) and constituted 83% of the total bacterial population (Fig. 1b). The late biofilm developmental phase (phase IV) was marked by a decline in biofilm-associated bacteria, decreasing from 1.2 × 10^9^ (OD_405_ of 0.28) to 4.4 × 10^8^ bacteria *per* mL by day 21 (OD_405_ of 0.10). Notably, this 2.8 fold bacterial number reduction did not correspond to an increase in planktonic bacteria, which stabilized at an OD_405_ of 0.06 at day 12. Intriguingly, despite a reduction of bacteria number, the biofilm’s size did not diminish. Instead, as shown by surface quantification and crystal violet staining, there was an expansion of the structure, evidence of the complexity of biofilm development (Fig. 1a, c, d).

These data indicate that a 21-day culture is predominantly composed of biofilm-organized *Leptospira*, establishing it as a suitable condition to explore the transcriptional landscape of pathogenic *Leptospira* in such a complex multicellular arrangement.

### Global transcriptomic response

**Fig. 2:**
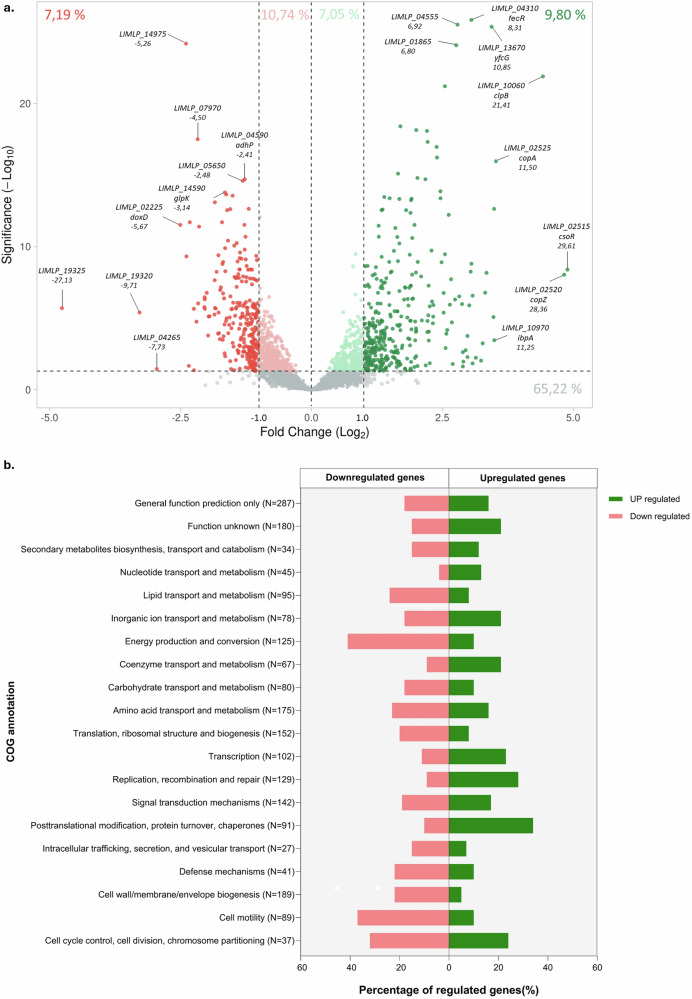
Differential gene expression in late *Leptospira* biofilms. **a** Volcano plot displaying differentially expressed genes in *L. interrogans* L495 21-day-old biofilm. Significantly upregulated genes are shown in green, downregulated in red, with the fold change threshold set at Log_2_FC ± 1 and adjusted *p*-value < 0.05 for statistical significance. Most differentially expressed genes are annotated with their identifier and respective fold changes. **b** COG category distribution of differentially expressed genes in 21-day-old biofilm. Each bar represents the percentage of genes within a COG category that are differentially expressed compared to the total gene number in each category (N), with upregulation in green and downregulation in red. The *y*-axis details the functional categories corresponding to each bar.

To gain insight into the cellular mechanisms underlying the bacterial adaptation and survival upon biofilm production, gene expression profiles of exponentially growing planktonic (5 days of culture) and late biofilm (21 days of culture) cells of *L. interrogans* serovar Manilae strain L495 were determined. Comparison of the planktonic and biofilm gene expression levels revealed a total of 1263 differentially expressed genes (DEGs) with an adjusted *p*-value < 0.05 (Fig. 2, Supplementary Table 1). Of these, 612 and 651 genes were up- and downregulated, respectively (Fig. 2a). They were distributed in similar proportions (32%) on the two chromosomes and the plasmid (Supplementary Fig. 1d). Interestingly, as shown by both the circular representation and the volcano plot, most of the upregulated genes had a foldchange above 2 whereas the downregulated genes showed a more modest change. (Fig. 2a, Supplementary Fig. 1d). Sixty percent of DEGs had a Clusters of Orthologous Groups (COG) annotation and the remaining 40% were screened and manually curated based on gene information and domain prediction (Supplementary Fig. 1c)^20^. Although the vast majority of DEGs were categorized as “Function unknown” or “General function of prediction only”, some genes could be reclassified, including ten transposases in the “Replication, recombination and repair” category. Late biofilm notably induced a downregulation in expression for numerous genes related to energy production and conversion, cell wall/membrane/envelope biogenesis, cell motility (Fig. 2b), indicating an overall decrease in cellular activity and metabolism. On the contrary, there was an upregulation of genes involved in transcription, replication, recombination and repair, or even posttranslational modification, protein turnover, and chaperones. A functional enrichment analysis using the KEGG (Kyoto Encyclopedia of Genes and Genomes^21^,) confirmed these observations with six pathways significantly regulated, including flagellar assembly, ribosome, and repair mechanisms (Supplementary Fig. 2).

### Attenuation of motility in late-stage biofilm

The swimming of leptospires in liquid environments is driven by a complex periplasmic flagellar structure composed of a basal body, hook, and filament. Flagellar rotation is powered by a motor located within the basal body. The self-assembly of its main components is ensured by a specific export apparatus that facilitates the transfer of flagellar filament proteins from the cytoplasm to the distal end of the growing structure.

**Fig. 3:**
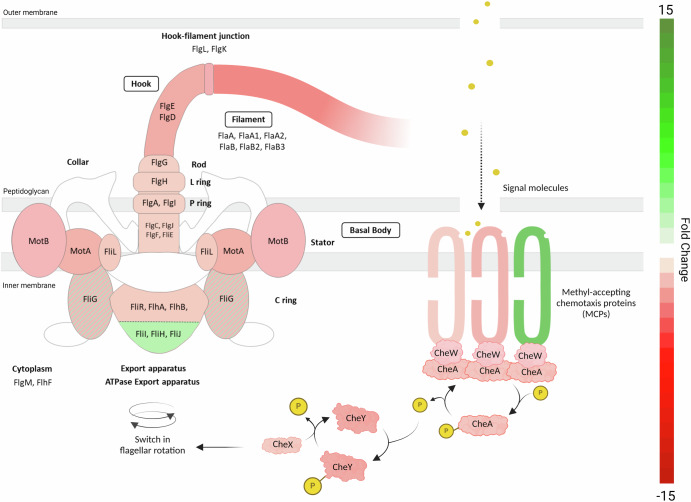
Motility and chemotaxis gene expression in *Leptospira* biofilms. This figure visually represents the gene expression changes associated with motility and chemotaxis in *L. interrogans* L495, mapped onto a schematic representation of their corresponding gene products in the bacterial structure. The diagram highlights key structural components such as the basal body, hook, and filament, and the associated chemotaxis machinery, providing a visual correlation between gene expression and functional protein localization. Gene products from downregulated genes are indicated in red, while those from upregulated genes are marked in green, underscoring a widespread downregulation of these genes within the biofilm context. If multiple paralogs of the same gene have different regulation, the protein is shaded in both colors.

39 genes participating in the flagellum structure and motility were differentially regulated, most of them being downregulated in biofilms (Fig. 3, Supplementary Table 2). Indeed, genes encoding three proteins of flagellar export apparatus, *fliR* (LIMLP_06705), *flhB* (LIMLP_06710) and *flhA* (LIMLP_06715), which belong to a 7-gene putative operon^22^, were downregulated. Downstream genes of this operon, *flhF* (LIMLP_06720) and *flhG* (LIMLP_06725) had also a decreased expression^23^. Genes *motA* (LIMLP_14630) and *motB* (LIMLP_14625) encoding two proteins of the stator of the flagellar motor were downregulated, as well as LIMLP_00125-encoded FliG that interacts with the stator. Another cluster of basal body genes, organized in operon and composed of *flgG* (LIMLP_06485), *flgA* (LIMLP_06490), *flgH* (LIMLP_06495), *flgI* (LIMLP_06500) and *flgJ* (LIMLP_06505) was also downregulated^22^. Genes encoding constituents of the hook (*flgD*, LIMLP_05750 and *flgE*, LIMLP_05755) and of the periplasmic flagellar filament (*flaA1*, LIMLP_13775; *flaA2*, LIMLP_13780; *flaA-*like, LIMLP_11310; *flaB4*, LIMLP_07475; *fcpA*, LIMLP_01630; *fcpB*, LIMLP_09180) had a lower expression when *Leptospira* formed biofilms.

Intriguingly, three genes encoding soluble proteins of the ATPase ring complex of the export apparatus^24^ (*fliH*, LIMLP_06770; *fliI*, LIMLP_06785; *fliJ*, LIMLP_06790) and a paralog of FliG (encoded by LIMLP_08925) were all upregulated in biofilm condition.

Genes associated with chemotaxis were also differentially expressed upon biofilm formation. Among the 10 chemotaxis-related genes identified, 9 were downregulated (Fig. 3, Supplementary Table 2). Those included seven genes involved in signal transduction that governs flagellar rotation, and three encoding membrane-bound chemotaxis sensory transducers, known as methyl-accepting chemotaxis proteins (MCPs). They play a pivotal role in bacterial adaptation to diverse stresses, cell survival, biodegradation, and signal transduction processes^25^. Of the 12 MCPs identified in the *L. interrogans* Manilae genome (as per MIST database^26^), three showed significant modulation in our dataset. Notably, LIMLP_06865 exhibited a striking upregulation with an 11.1 fold-change (FC), while two others, LIMLP_17325 and LIMLP_17355, were downregulated (FC −2.0 and FC −1.8, respectively). MCPs initiate a downstream phosphorylation cascade within the bacterial chemotaxis system. Our dataset revealed downregulation in key components of this system. This included two response regulator CheY-encoding genes (LIMLP_07450, FC −3.1; LIMLP_14950, FC −1.8), the kinase CheA (LIMLP_07440, FC −2.3), CheD (LIMLP_07425, FC −2.1), CheW (LIMLP_11115, FC −1.6), the phosphatase CheX (LIMLP_05655, FC −2.1), and an anti-sigma factor antagonist (LIMLP_07445, FC −2.7) within the chemotaxis operon of *L. interrogans* (LIMLP_07420-LIMLP_07460) (Fig. 3).

Our findings collectively indicate that leptospiral cells undergo extensive downregulation of motility and chemotaxis within a biofilm reflecting that active motility may be less essential compared to planktonic conditions.

### Decrease of cell division is indicative of a quiescent state

After three weeks of culture, when biofilm formation has reached a plateau, we observed a reduced cell division capability of *L. interrogans*, accompanied by a decline in cell growth (Fig. 1).

**Fig. 4:**
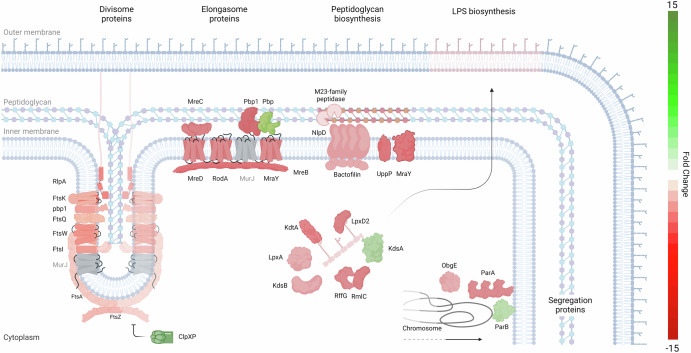
Schematic representation of cell division pathways modulation in *Leptospira* biofilms. This figure depicts the downregulation of genes involved in cell division within *L. interrogans* L495 forming biofilms. The diagram maps key components involved in peptidoglycan biosynthesis, divisome and elongasome structures, LPS biosynthesis, and chromosome partitioning to their corresponding cellular locations. Gene products from downregulated genes are indicated in red, while those from upregulated genes are marked in green, and denote a global downregulation of these pathways in biofilm-forming *Leptospira*. Inspired from refs. ^100**–**102^.

In our dataset, ten genes associated with cell division were downregulated (Fig. 4, Supplementary Table 3), including six genes (*ftsAIKQWZ*) encoding components of the divisome complex. Notably, the *ftsZ* gene (LIMLP_02595, FC −1.7), crucial for cytokinesis and divisome assembly^27^, was downregulated. Parallel to this, genes encoding the ClpXP protease components—ClpX (LIMLP_06920, FC 1.7) and ClpP (LIMLP_06915, FC 2.2)—were upregulated. These genes are known to degrade FtsZ in *E. coli*^28^.

Additionally, a gene encoding a bactofilin domain-containing protein (LIMLP_13115), was also downregulated. This domain is ubiquitous in bacterial cytoskeletal proteins mediating polar localization of a cell wall synthase in various organisms^29–31^.

Furthermore, *parA* (LIMLP_01155), involved in chromosome partitioning^32^, showed downregulation whereas genes annotated as chromosome-partitioning *parA* and *parB* (LIMLP_17770 and LIMLP_17775) were slightly upregulated (FC 1.6 and 1.7). The gene encoding ObgE (LIMLP_03590), essential for chromosome partitioning in *E. coli*^33^, was downregulated, as was LIMLP_00285, encoding the peptidoglycan transglycosylase RlpA, implicated in daughter cell separation and rod shape maintenance in *Pseudomonas aeruginosa*^34^.

Besides divisome downregulation, transcripts for elongasome components, such as *mreB* (LIMLP_06155), *mreC* (LIMLP_06160), *mreD* (LIMLP_06165), *ftsI* (LIMLP_06170), *rodA* (LIMLP_06175), and *mrcA*/*pbp1* (LIMLP_15065), were also downregulated (Fig. 4, Supplementary Table 3). These components catalyze peptidoglycan biosynthesis and are crucial for maintaining cell shape. Conversely, the *pbp* gene (LIMLP_08630) was slightly upregulated (FC 1.5).

Similarly, several genes coding for putative components of the peptidoglycan biosynthesis and cell wall remodeling machinery had a lower expression in biofilms. These genes comprise six peptidase M23 family genes, including four *nlpD* genes (LIMLP_13120, LIMLP_06825, LIMLP_07655, LIMLP_17195), *uppP* (LIMLP_07100), *mraY* (LIMLP_09270) as well as a gene coding for a transglycosylase with a soluble lytic transglycosylase (SLT) domain (LIMLP_19130, FC −1).

Lastly, numerous genes involved in lipopolysaccharide (LPS) biosynthesis, including those responsible for lipid A biosynthesis and maturation were downregulated, such as *lpxD2* (LIMLP_17670), *lpxA* (LIMLP_01675), *kdtA* (LIMLP_11320) or *kdsb* (LIMLP_10685). However, the *kdsA* gene (LIMLP_07520), also linked to peptidoglycan and LPS biosynthesis, was slightly upregulated. Additionally, *rffG*/*rmlB* (LIMLP_10515, FC −1.6) and *rmlC* (LIMLP_10525, FC −9.0) genes, within the *rfb* gene cluster involved in O-antigen biosynthesis, were slightly downregulated.

Taken together, our results indicate that in *L. interrogans*, key cellular processes necessary for cell growth including the divisome and elongasome complexes, peptidoglycan biosynthesis, and the synthesis of lipopolysaccharides (LPS) and O antigen, undergo significant modulation.

### Modulation of energy metabolism in *L. interrogans* biofilm

**Fig. 5:**
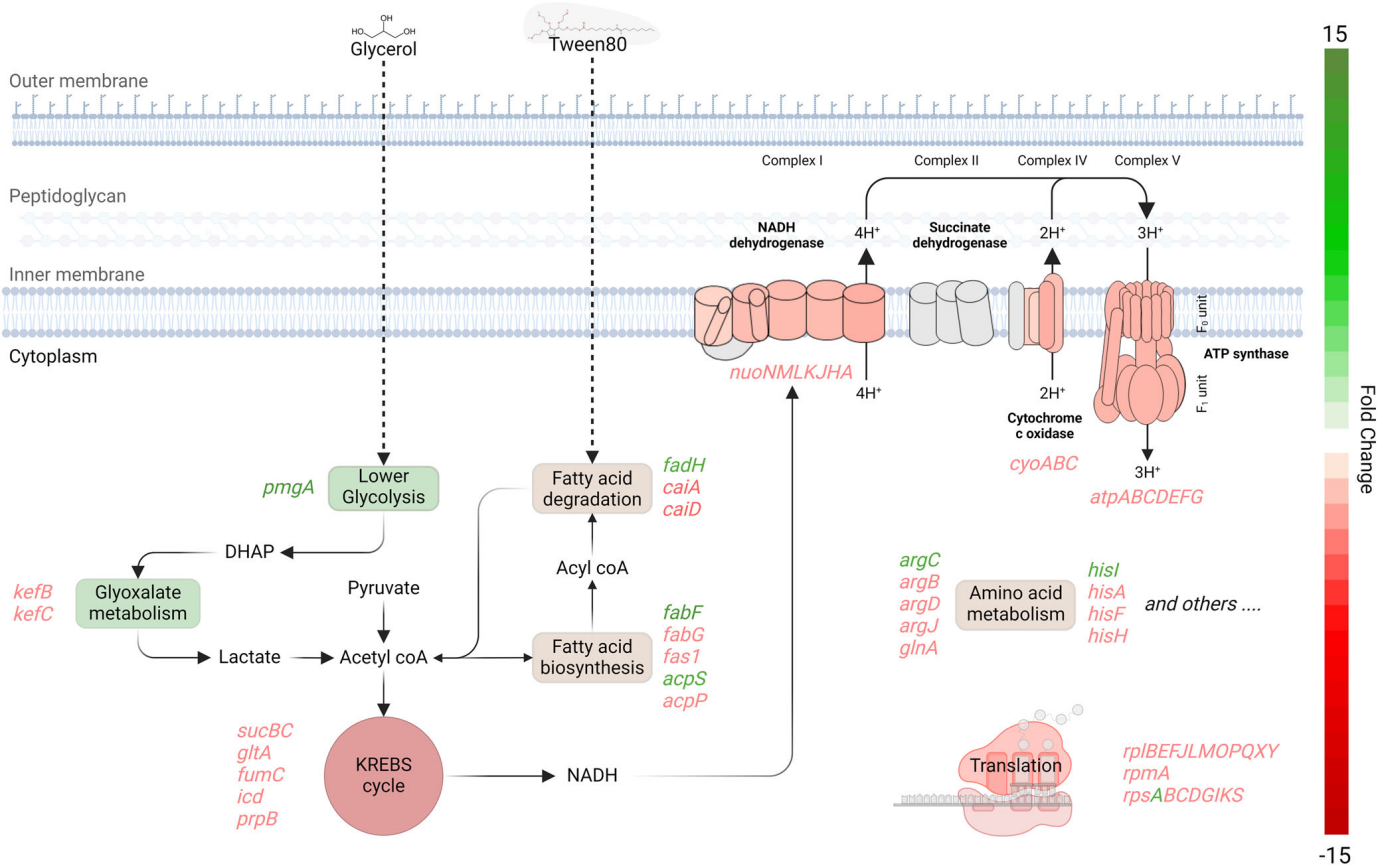
Transcriptomic modulation of metabolic pathways in *Leptospira* biofilm. This diagram highlights expression changes in genes involved in metabolism and energy production in *L. interrogans* L495 as it forms a biofilm. Upregulation in genes related to lower glycolysis, potentially in conjunction with an activated glyoxylate pathway through DHAP, is indicative of an adaptive shift to increased lactate production, feeding into pyruvate synthesis. Varied gene expression in the fatty acid degradation pathways contrasts with the clearly downregulated Krebs cycle enzymes which, coupled with the downregulation of key enzymes in the electron transport chain, suggest restrained ATP production, indicating a shift toward an energy-thrifty metabolic state. The comprehensive downregulation of genes for both 50S and 30S ribosomal subunits points to a minimally active translational machinery, consistent with a lower metabolic requirement in the biofilm state. Gene products from downregulated pathways are denoted in red, and those from upregulated genes in green, visually representing the scale of transcriptomic reprogramming within the bacterial cell.

Among the 97 genes identified as involved in global energy metabolism in *Leptospira* in our dataset, the majority (81 genes) were downregulated, while 16 genes showed upregulation (Fig. 5, Supplementary Table 4).

*Leptospira* primarily relies on long-chain fatty acids (over C-15) as its major carbon and energy sources, predominantly utilizing beta-oxidation for energy derivation, as sugars are neither fermented nor utilized as carbon sources^35^.

Several enzymes of this pathway were differentially expressed. Five homologs of *caiA*, encoding an Acyl-CoA dehydrogenase that catalyzes the conversion of acyl-CoA to enoyl-CoA, were downregulated (FC range −1.8 to −2.3). Conversely, *fadH* (LIMLP_08580), encoding a 2,4-dienoyl-CoA reductase involved in unsaturated fatty acid degradation, was upregulated (FC 2.3). This upregulation aligns with the degradation of Tween80, the primary fatty acid source in EMJH medium, predominantly composed of oleic acid, an unsaturated fatty acid (C18). Additionally, two enoyl-CoA hydratases (*caiD*/LIMLP_03845 and *caiD*/LIMLP_08150) and a putative 3-ketoacyl-CoA thiolase (LIMLP_04665) were downregulated (FC −1.5, −1.9, and −1.7, respectively).

The bacterial fatty acid synthesis pathway also exhibited varied gene expression modulation. For instance, *fabF* (LIMLP_11535), encoding a beta-ketoacyl synthase responsible for acyl-ACP elongation, was strongly upregulated (FC 6.2), while *fabG* genes, responsible for subsequent steps in the fatty acid synthesis cycle, were downregulated (LIMLP_14230, FC −2.7 and LIMLP_00095, FC −1.5). Furthermore, genes LIMLP_07405 (encoding a biotin carboxyl carrier protein of acetyl-CoA carboxylase) and *fas1* (LIMLP_16970, encoding a fatty acid synthase subunit) both contributing to malonyl-CoA synthesis, a precursor in fatty acid synthesis, were downregulated (FC −1.8 and −2.4, respectively). Finally, *acpS* (LIMLP_13460), encoding a holo-[acyl-carrier-protein] synthase, was upregulated (FC 1.4), while *acpP*, encoding an acyl carrier protein (ACP), was downregulated (FC −1.6). The products of these two genes are critical in the fatty acid synthesis pathway.

*Leptospira* does not utilize six-carbon sugars for energy production. However, we observed significant modulation in genes associated with the triosephosphate metabolism pathway, which is linked to lower glycolysis, such as LIMLP_07055 (encoding a Glycerate 2-kinase, FC 3.2) and LIMLP_18450 (encoding a phosphoglycerate mutase, *pmgA*, FC 3.3). In addition, in the context of glycerol degradation, we noted downregulation in two glycerol kinases, LIMLP_14590 (FC −3.1) and LIMLP_08930 (FC −1.6). These enzymes are involved in producing glycerol 3-phosphate, which is subsequently converted to dihydroxyacetone phosphate (DHAP) via glycerol 3-phosphate dehydrogenase (LIMLP_17400, FC 2.3; LIMLP_12830, FC −1.8). These results suggest a preferential utilization of glycerate degradation pathways over glycerol, leading to pyruvate production.

Interestingly, components of the methylglyoxal pathway were also regulated. While the methylglyoxal synthase (LIMLP_03805) and glyoxalase II genes (LIMLP_08945 and LIMLP_01865) were significantly upregulated (FC 5.2, 2.6 and 6.8, respectively), triosephosphate isomerase (LIMLP_10350), and glyoxalase I (LIMLP_00375) were slightly downregulated. KefB (LIMLP_11025, FC −1.8 and LIMLP_17560, FC −1.9) and KefC (LIMLP_17535, FC −1.6) transporters were also slightly downregulated.

In line with the observed reduction in energy production, we noted a significant downregulation of several enzymes integral to the tricarboxylic acid cycle, including a fumarate hydratase (LIMLP_00845, FC −2.0), a succinate-CoA ligase (LIMLP_12885, FC −2.1), a succinyl transferase of the 2-oxoglutarate dehydrogenase complex (LIMLP_12355, FC −1.4), an isocitrate dehydrogenase (LIMLP_01230, FC −2.0), a citrate synthase (LIMLP_02815, FC −1.7), and a 2-methylisocitrate lyase (LIMLP_11605, FC −2.3).

Consistently, our data revealed a downregulation of several components within the electron transport chain (Fig. 5, Supplementary Table 4). Notably, genes encoding components of the Complex I (*nuoN, nuoM, nuoL, nuoK, nuoJ, nuoH*, and *nuoA*, LIMLP_03705-03730 and LIMLP_03760, respectively) and of the complex IV (*cyoA*, *cyoB cyoC* ; LIMLP_01100-01110) as well as the entire operon (LIMLP_06045-06080) encoding the subunits of the ATP-synthase complex were all downregulated.

### Downregulation of protein synthesis machinery

Among the 59 genes associated with the downregulated ribosome KEGG pathway, 20 were similarly differentially expressed in our model strain L495 (Fig. 5, Supplementary Table 4). These genes (*rpl* and *rps*), encoding components of the large 50S and small 30S ribosomal subunits, were downregulated with fold changes ranging between −1.5 and −2.3. Furthermore, the *sua5* gene (LIMLP_08535, FC −1.9), involved in ribosome biogenesis, and several genes involved in aminoacyl-tRNA biosynthesis, such as *gatA* (LIMLP_07120, FC −1.8), *gatB* (LIMLP_01160, FC −1.5), *asnS* (LIMLP_10110, FC −1.4), and *thrS* (LIMLP_12290, FC −1.6), also exhibited downregulation. This suggests a substantial decrease in translational activity during biofilm formation. However, certain genes were upregulated, likely to maintain a minimal translation machinery necessary for cell survival. These include *rpsA* (LIMLP_02720, FC 2.1), encoding the ribosomal protein S1 essential for mRNA translation in *E. coli*^36^, *pcnB* (LIMLP_09645, FC 2.4), encoding a polyA polymerase, and *def* (LIMLP_07370, FC 1.9), encoding a peptide deformylase.

Intriguingly, *bipA* (LIMLP_11740) and *cspR* (LIMLP_02725) genes were upregulated by 2.1 and 2.1-fold, respectively. These genes encode a GTP-binding protein and a tRNA/RNA methyltransferase, respectively, known to enhance survival and growth under stress conditions in other bacteria^37–40^. Notably, *bipA* mutants have been associated with reduced biofilm formation, and virulence in *P. aeruginosa*^41^.

### Alteration in amino acids metabolic processes

A complex regulatory pattern was observed in the amino acid metabolism pathways, characterized by a mix of upregulation and downregulation within the same biosynthetic pathways (Fig. 5, Supplementary Table 4). While two genes of the arginine biosynthesis pathway were upregulated, *argC* (LIMLP_08670, FC 1.6) and LIMLP_06420 (FC 1.8), four showed downregulation, *argJ* (LIMLP_16730, FC −1.9), *argD* (LIMLP_08770, FC −1.5), *argB* (LIMLP_18295, FC −1.6), and *glnA* (LIMLP_11985, FC −2.0). A similar profile was observed in histidine metabolism, with one gene being upregulated (*hisI*/LIMLP_02360, FC 2.4), and three genes being downregulated (*hisH*/LIMLP_00600, FC −1.3; *hisA*/LIMLP_00605, FC −1.4; *hisF*/LIMLP_07125, FC −1.7).

Leucine metabolism also exhibited a contrasting pattern, since *leuA* was upregulated (LIMLP_08570, FC 2.2) while *leuB* was downregulated (LIMLP_08775, FC −1.4). Lysine metabolism, on the other hand, was predominantly upregulated, with *dapB* (LIMLP_13475, FC 1.5), *dapA* (LIMLP_13480, FC 1.5), and *dapF* (LIMLP_00400, FC 2.1) all showing increased expression. In contrast, the L-lysine 2,3-aminomutase encoding gene *ablA* (LIMLP_19035), involved in lysine degradation, was downregulated (FC −1.6), suggesting a shift towards lysine production.

Metabolism of amino acids such as alanine, tyrosine, and glycine appeared slightly stimulated, whereas pathways for glutamate and serine were repressed. This is further indicated by the slight downregulation of transport-related genes *putP* (LIMLP_08865, FC −1.7) for proline and *gltP* (LIMLP_00320, FC −1.3) for glutamate, suggesting reduced transport of these amino acids.

### C-di-GMP metabolic enzymes and effectors

In the context of biofilm formation, the examination of genes involved in cyclic di-GMP metabolism was crucial due to this secondary messenger’s key role in biofilm regulation^10,42^. Our dataset identified low amplitude yet significant modulations of 11 out of the 25 enzymes responsible for c-di-GMP metabolism in *L. interrogans*^43^: three genes associated with c-di-GMP degradation were upregulated (LIMLP_07630, LIMLP_00730, LIMLP_09580) (Supplementary Table 5). In contrast, genes coding for GGDEF domain-containing enzymes, which synthesize c-di-GMP, were predominantly downregulated (LIMLP_05450-05470). The remaining 14 enzymes showed no significant expression changes. Further investigation into c-di-GMP effector proteins, as previously identified in *L. biflexa*^44^, revealed that out of the 41 effectors found in the saprophytic strain, 22 homologous genes were present in *L. interrogans*, of which three were significantly modulated. This included downregulation of a guanylate cyclase with a GAF domain and a PilZ domain-containing protein (LIMLP_06095 and LIMLP_03855), alongside upregulation of a REC domain-containing phosphodiesterase (LIMLP_12520) (Supplementary Table 9).

### Maintenance of redox homeostasis

**Fig. 6:**
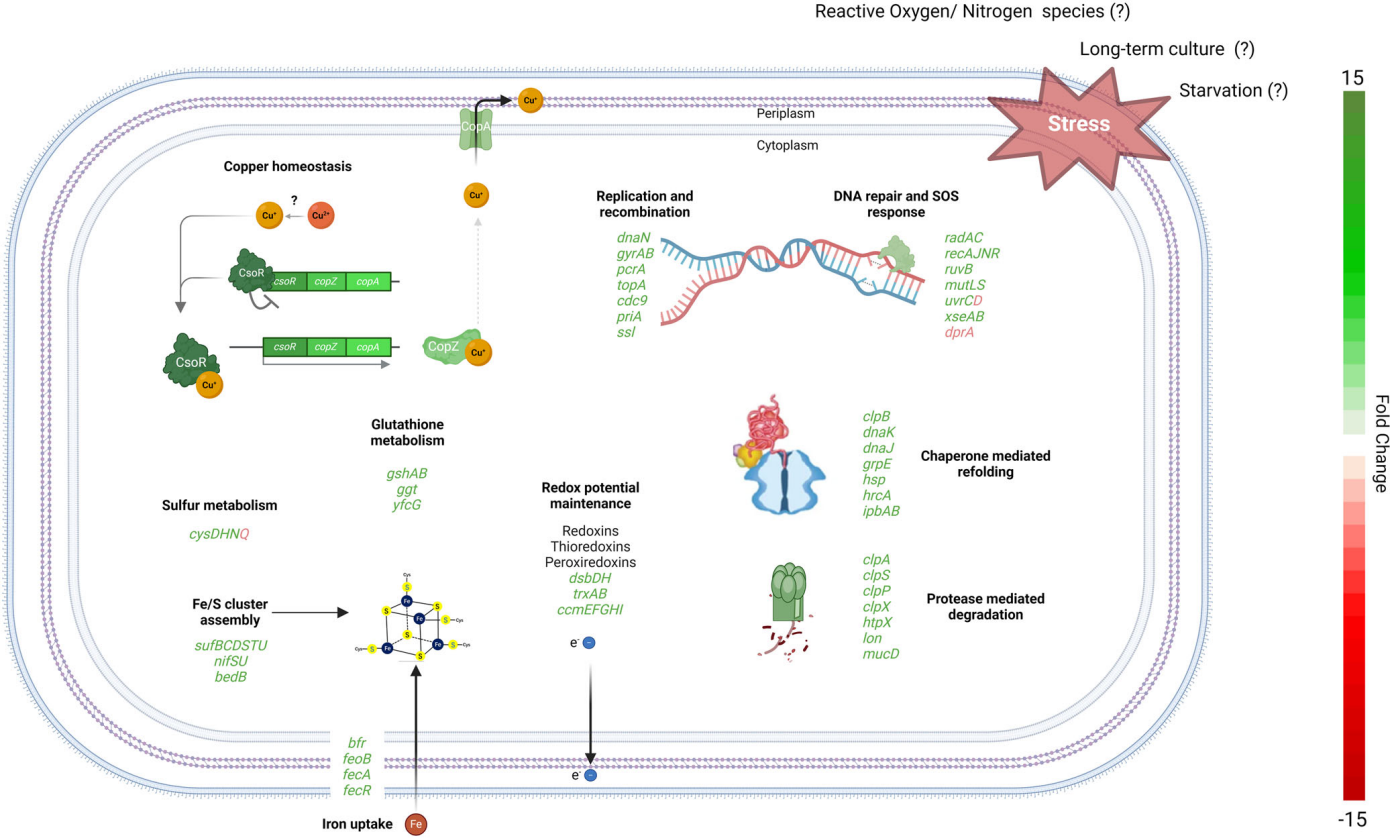
Complex stress response in *Leptospira* biofilm. This cartoon recapitulates the variety of stress responses in *L. interrogans* L495 when it forms a biofilm. The figure maps out key genetic responses to various stress factors, with most related genes showing upregulation. DNA repair machineries, molecular chaperones, ATP-dependent proteolytic complexes, sulfur reduction, iron-sulfur biosynthesis pathways, and copper homeostasis are highlighted. The diagram suggests an orchestrated upregulation of genes across these systems, underlining a sophisticated adaptive strategy to the multifaceted stresses encountered within the biofilm.

Several genes encoding enzymes of the redox homeostasis and defenses against oxidative stress were significantly upregulated (Fig. 6, Supplementary Table 6). Our study revealed the upregulation of two peroxiredoxins (LIMLP_03055, FC 2.4; LIMLP_12405, FC 1.9), enzymes known for reducing hydrogen peroxide and organic hydroperoxides. Consistent with the increased expression of peroxiredoxins was the significant upregulation of the thioredoxin (Trx) system. The thioredoxin (*trxA*, LIMLP_09870, FC 2.5) and thioredoxin reductase (*trxB*, LIMLP_07165, FC 2.0) and three additional genes encoding putative thioredoxins (LIMLP_11770/*tlpA*, FC 2.3; LIMLP_07145, FC 2.7; LIMLP_18140, FC 2.5) were also upregulated. These thioredoxins and thioredoxin reductase could be involved in regenerating the reduced form of peroxiredoxins and/or disulfite isomerase.

In addition, a substantial upregulation of the glutathione system components was also observed. The *gshA* (LIMLP_08995) and *gshB* genes (LIMLP_08990), both critical for the glutathione (GSH) biosynthesis, showed a marked increase in expression (FC 2.6 and 2.6, respectively). Two glutaredoxin genes (LIMLP_08980 and LIMLP_08985) were upregulated (FC 2.5 and 2.6, respectively). Moreover, the *ggt* gene (LIMLP_09000, FC 1.6), involved in GSH metabolism, was modestly upregulated, suggesting an increased turnover and maintenance of intracellular GSH pools. Notably, the *yfcG* gene (LIMLP_13670) whose expression was confirmed by RT-qPCR (Supplementary Fig. 3), exhibiting the highest upregulation in this cluster (FC 10.9), encodes a GSH-dependent disulfide-bond oxidoreductase that potentially plays a role in modulating protein thiol-disulfide status and supporting the assembly and recycling of iron-sulfur clusters under oxidative conditions. Altogether, these findings indicate a strategy to increase GSH levels either for maintaining the redox balance or to function as cofactor for GSH peroxidases glutaredoxins.

Genes participating in the maintenance of correct disulfides in periplasmic proteins also exhibited an increased expression in our conditions. The *dsbD* gene was greatly upregulated (LIMLP_11965, FC 4.5) along with a *dsbH-like* (LIMLP_04015, FC 3.1), a disulfide reductase.

The NADPH-dependent FMN reductase (LIMLP_16870, FC 5.9) and quinolinate synthase A (LIMLP_02615, FC 2.3) were also highly overexpressed in our dataset.

Furthermore, we noted a concurrent upregulation of genes required for cytochrome C maturation (*ccmG*/LIMLP_04655; *ccmEFHI/*LIMLP_13635-13650). This suggests an increased demand for C-type cytochromes, which are essential for maintaining respiratory cytochrome oxidase activity.

These data collectively show an orchestrated upregulation of the several different antioxidant systems, aimed at enhancing the cell’s capacity to maintain a proper redox homeostasis (Fig. 6, Supplementary Table 6).

### Regulation of iron-sulfur (Fe–S) clusters biosynthesis pathways

The Fe–S cluster assembly, a complex and multi-step process, is highlighted by the upregulation of the SUF system (Fig. 6, Supplementary Table 6). This includes the *suf* operon—encompassing *sufT*, *sufU*, *sufS*, *bedB*, *sufD*, *sufC*, in addition to *sufB*, *nifS*, and *nifU* (spanning LIMLP_14555 to LIMLP_13200)—indicative of an elevated need for Fe–S cluster synthesis, particularly under stress conditions that compromise cluster stability.

Sulfur and cysteine metabolism genes *cysN*, *cysD*, *cysH* (LIMLP_17220 to LIMLP_17230) and *cysE* (LIMLP_00290) were upregulated, suggesting the synthesis of essential sulfur components for Fe–S cluster construction. Conversely, the downregulation of an EamA-like transporter (LIMLP_02205), potentially involved in cysteine and its metabolite export, suggests a cellular inclination to retain high intracellular cysteine levels, a key sulfur source for cysteine desulfurases, such as SufS and NifS.

Iron acquisition, integral to Fe–S cluster formation, seems modulated, with the downregulation of a *fecA* gene (LIMLP_04270) putatively involved in iron uptake. This suggests a diminished demand for external iron, likely due to adequate iron availability within the cells. This premise is supported by the subtle changes in *feoB* expression and the upregulation of bacterioferritin (LIMLP_06430), which indicates an excess of iron necessitating storage. The striking upregulation of *fecR*, confirmed by RT-qPCR (LIMLP_04310, FC 8.3, Supplementary Fig. 3), encoding a regulatory protein known to bind the FecI sigma factor, the latter controlling the *fec* operon’s transcription in *E. coli*^45^, aligns with this notion. The elevated FecR levels may serve to sequester FecI, preventing the initiation of iron uptake operons, coherent with the observed downregulation of FecA and its presumed regulation by FecI.

In summary, the gene expression modulations suggest that the bacteria are actively reinforcing the pathways responsible for Fe–S cluster biosynthesis, possibly because of oxidative stress.

### A proteotoxic stress response in *L. interrogans* biofilm

An overall proteotoxic stress response was triggered, including a significant overexpression of heat shock proteins/molecular chaperones and associated factors (Fig. 6, Supplementary Table 6). For instance, the whole DnaK machinery encompassing the molecular chaperone DnaK of the HSP70 family (LIMLP_15115, FC 3.9), and its cofactors DnaJ (LIMLP_15120, FC 3.7) and GrpE (LIMLP_15110, FC 3.6) as well as the HrcA repressor that control their expression (LIMLP_15105, FC 3.5) were upregulated. Remarkably, *clpB* (LIMLP_10060), encoding a chaperone protein that cooperates with DnaK/DnaJ/GrpE in dissolving misfolded protein aggregates showed a significant increase in expression (FC 21.4). Genes encoding small heat shock proteins such as IbpA (LIMLP_10970, FC 11.3) and IbpB (LIMLP_10975, FC 9.6) were also notably upregulated, as well as a gene encoding Hsp90 (LIMLP_12325, FC 3.7).

In addition to factors involved in protein refolding, genes encoding components of proteolytic complexes were also upregulated. This includes the *clpP* (LIMLP_06915, FC 2.2) peptidase gene, its two ATPase cognate complexes, *clpA* (LIMLP_09005, FC 2.8) and *clpX* (LIMLP_06920, 1.7), and its cofactor-encoding *clpS* gene (LIMLP_09010, FC 3.6). A gene encoding an ATP-dependent Lon protease (LIMLP_14705, FC 1.7), a gene having a Lon substrate binding domain (LIMLP_07500, FC 2.8) but lacking the AAA ATPase domain as well as a gene encoding a DegQ-type serine protease MucD (LIMLP_03390, FC 2.8) and the membrane-bound HtpX protease (LIMLP_18560, FC 1.8) had an increased expression.

The collective overexpression of chaperones and proteolytic complexes suggests that the bacteria are mobilizing a comprehensive defense mechanism against proteotoxic stress. This involves not only the protection and repair of existing proteins but also the removal of irreversibly damaged proteins, possibly to maintain cellular integrity. In addition, the upregulation of multiple proteolytic complexes might also indicate proteolysis-based regulation, i.e. the rapid breakdown of factors that are no longer needed.

### Enhanced SOS response and DNA repair in biofilm state

Genes encoding key SOS response proteins such as *radA* (LIMLP_05535, FC 1.8) and *radC* (LIMLP_11400, FC 4.6) showed increased expression, highlighting enhanced repair of DNA breaks (Fig. 6, Supplementary Table 6). Homologous recombination facilitators, including RuvB (LIMLP_03395, FC 3.1) and RecA (LIMLP_08665, FC 1.8), were upregulated, as well as factors involved in mismatch repair including MutS (LIMLP_08800, FC 1.7) and MutL (LIMLP_12570, FC 1.8). The nucleotide excision repair pathway is activated, as seen with the upregulation of *uvrC* (LIMLP_08725, FC 1.9), *xseA* (LIMLP_07755, FC 1.7) and *xseB* (LIMLP_07750, FC 2.8), alongside *alkA* (LIMLP_11765, FC 1.9). Notably, *rad50* (LIMLP_16525, FC 1.8) points to an increased capacity for double-strand break repair.

We also observed an upregulation of key replication factors such as the beta sliding clamp of the DNA polymerase (*dnaN*/LIMLP_00010, FC 1.4), DNA gyrase subunits (*gyrB*/LIMLP_00025, FC 1.4 and *gyrA*/LIMLP_00030, FC 1.3), a replication initiation factor (LIMLP_08300, FC 2.1), DNA topoisomerase I (*topA*/LIMLP_10290, FC 1.9) and ATP-dependent helicases (*pcrA*/LIMLP_07935, FC 1.6; *priA*/LIMLP_07570, FC 1.6 and *ssI*/LIMLP_08885, FC 2.2).

Altogether, the cell appears to be mobilizing a comprehensive response to maintain DNA integrity by combining increased DNA repair capacity with enhanced replication and recombination activities. This response underlines the cell’s strategic priority to ensure genome stability in the face of stress encountered within biofilm environments.

### Biofilm-induced oxidative stress response diverges from peroxide induction

**Fig. 7:**
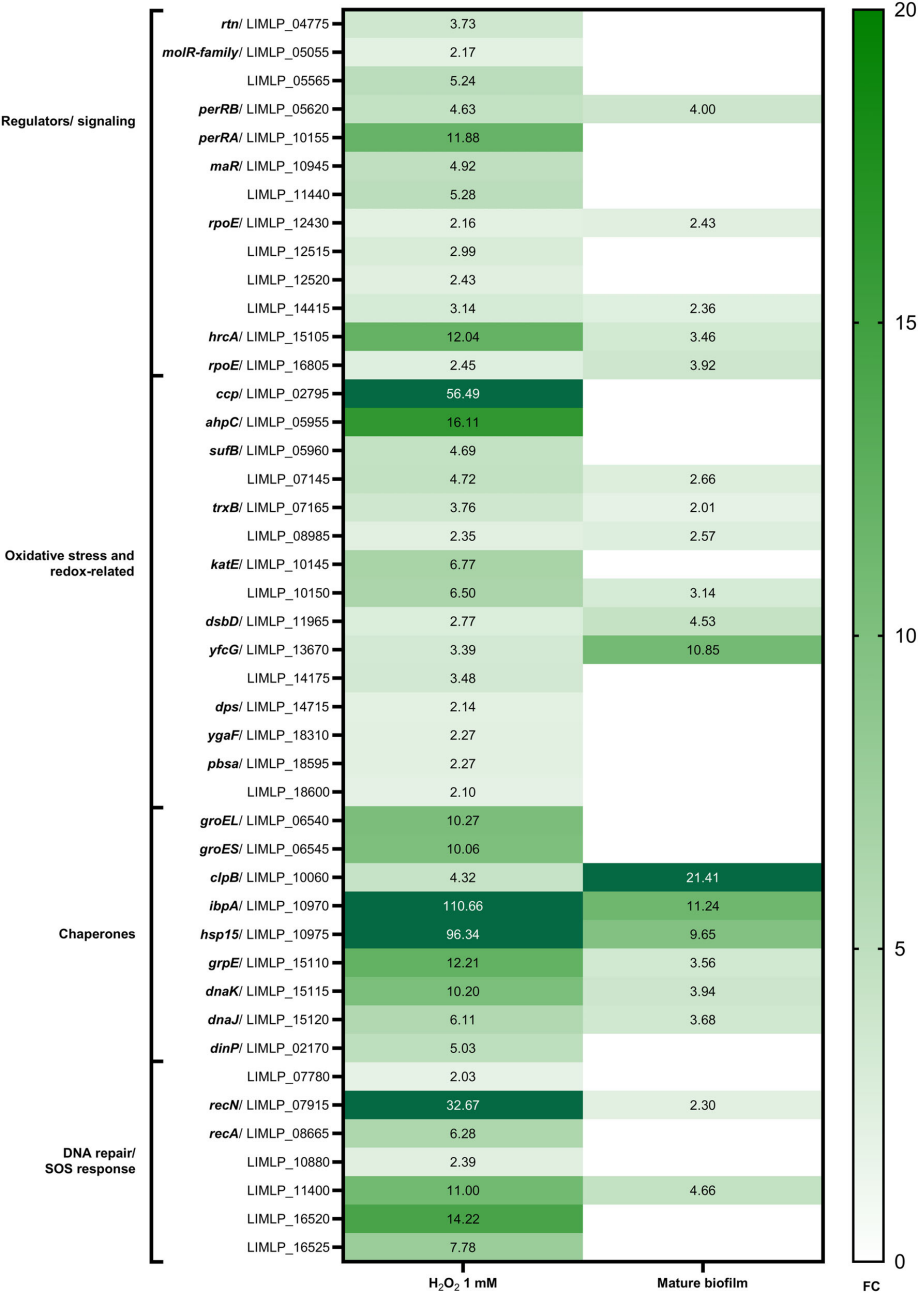
Comparative analysis of gene expression in *L. interrogans* under oxidative and biofilm-induced stress. This heatmap displays the upregulated gene expression profiles when *L. interrogans* L495 is exposed to 1 mM H_2_O_2_ for one hour (as determined in ref. ^46^) compared to a 21-day mature biofilm (as determined in this study). The genes are categorized by function, with their identifiers and fold changes. The color scale transitions from white to green, corresponding to an increase from lower to higher FC values. The late biofilm-associated oxidative stress response in *L. interrogans* suggests a distinct response and mechanisms, diverging from peroxide-induced pathways.

To further understand the stress signature associated with biofilm development, we compared our findings with genes from another dataset that were significantly deregulated (*p*_adj_ <0.05) and exhibiting a |log_2_FC| >1 following a 1-hour exposure to 1 mM H_2_O_2_, a known potent inducer of oxidative stress^46^. Within this overlapping gene set, 155 (31.3%) exhibited significant modulation in our biofilm dataset, with 138 (27.8%) displaying concordant regulation patterns (Supplementary Fig. 4, Supplementary Table 7). Moreover, of the 44 genes identified by the authors as upregulated in response to both lethal and sub-lethal concentrations of H_2_O_2_, 19 were similarly upregulated within the late biofilm state, as illustrated in Fig. 7. The peroxide stress regulator encoded by *perRA* (LIMLP_10155), as well as members of its regulon that are crucial oxidative stress-related genes, such as *ccp* (Cytochrome C Peroxidase), *ahpC* (peroxiredoxin), and *katE* (catalase), did not show significant modulation in the biofilm. In contrast, a second peroxide stress regulator encoded by *perRB* (LIMLP_05620, FC 4.0) was consistently overexpressed in both conditions. Regulatory and signaling genes, including *rpoE*-like genes (LIMLP_12430, FC 2.4; LIMLP_16805, FC 3.9), which encode a unique class of alternative ECF sigma factors associated with various stress responses, exhibited slight upregulation under biofilm conditions. The *hrcA* gene (LIMLP_15105), robustly expressed following H_2_O_2_ exposure, demonstrated a similar increase in expression in the biofilm. This trend of upregulation within the biofilm extended to the *hrcA*-regulated genes *grpE*, *dnaK*, and *dnaJ*, aligning with the results observed upon H_2_O_2_ stress. Other chaperones were generally upregulated across both conditions, apart from the GroEL/GroES complex, which were not modulated during late biofilm formation. The DNA repair and SOS response also diverged, with only four genes upregulated in the biofilm, including *radC* (LIMLP_11400, FC 4.7).

These findings suggest that the oxidative stress-like response encountered in the late biofilm state may arise from sources or through mechanisms distinct from those triggered by a direct exposure to peroxide.

### Predominant upregulation of the copper detoxification operon

A striking overexpression of genes associated with copper detoxification in other bacterial genera was captured in our dataset. The so-called “copper-sensitive operon”, including the repressor protein CsoR, which increased gene expression was confirmed by RT-qPCR (LIMLP_02515, FC 29.6, Supplementary Fig. 3), and the copper homeostasis proteins CopZ (LIMLP_02520, FC 28.3) and CopA (LIMLP_02525, FC 11.4) were the most highly upregulated genes in our conditions (Fig. 6, Supplementary Table 6). CsoR is known to function as a transcriptional repressor^47–49^ responding to copper in a number of bacterial species. Upon copper binding, CsoR dissociates from the DNA promoter region, resulting in the *csoR*/*copZA* operon transcription.

CopZ encodes a copper-transporting ATPase protein, also known as the copper chaperone protein, which is crucial for transporting copper ions across different compartments within the cell. In *L. interrogans*, CopZ contains the typical Cu^+^ binding motif MXCXXC. CopZ is also known to buffer cytosolic copper levels and, in certain organisms, is directly involved in delivering copper to CsoR^50^. CopZ is also involved in delivering Cu^+^ to CopA, a P-type ATPase that catalyzes the efflux of monovalent copper. This efflux system is thought to export copper in the periplasm, helping to regulate and remove excess copper from the reducing cytoplasm environment^51^. Therefore, based on this highly conserved mechanism, upregulation of the *csoR*/*copZA* operon suggests that *Leptospira* needs to mitigate the effects of a copper stress in biofilms.

The late biofilm environment also prompted the upregulation of other genes potentially related to counteracting excessive metals levels. A significant upregulation of a hypothetical Resistance-Nodulation-Division (RND) transporter from the HAE1 family, annotated as *acrB* (LIMLP_01780, FC 3.3) was noticed. Adjacent to *acrB*, another gene annotated as *tolC* (LIMLP_01775, FC 2.3) was also upregulated. The co-localization and upregulation of these genes suggest the presence of an efflux pump system akin to the AcrA/B-TolC system observed in *E. coli*^52^. Surprisingly, *acrR*, a transcriptional repressor of the *acrA*/*B* operon in *E. coli* was also upregulated (FC 3.0)^53^, indicating a more complex response in our model organism.

In addition, the upregulation of genes like *czcD* (LIMLP_08515, FC 1.5), encoding a Co/Zn/Cd efflux pump, and *mdlB* (LIMLP_00720, FC 2.1), coding for a molybdenate ABC transporter, reflects a global response to various metal stresses. Another metal-responsive transcriptional repressor, *arsR* (LIMLP_14415, FC 2.4), that regulates genes involved in efflux and detoxification of Cu/Ni and heavy metals in other bacteria^54–56^ was also upregulated.

The significant upregulation of the copper-sensitive operon and related metal detoxification genes suggests an elevated presence of metal ions capable of eliciting such a transcriptomic response.

### Consistent virulence in *Leptospira* biofilms despite reduced metabolic rates

We evaluated the maintenance of *Leptospira* virulence within biofilms, especially taking into account the diminished metabolic activity and increased stress responses.

**Fig. 8:**
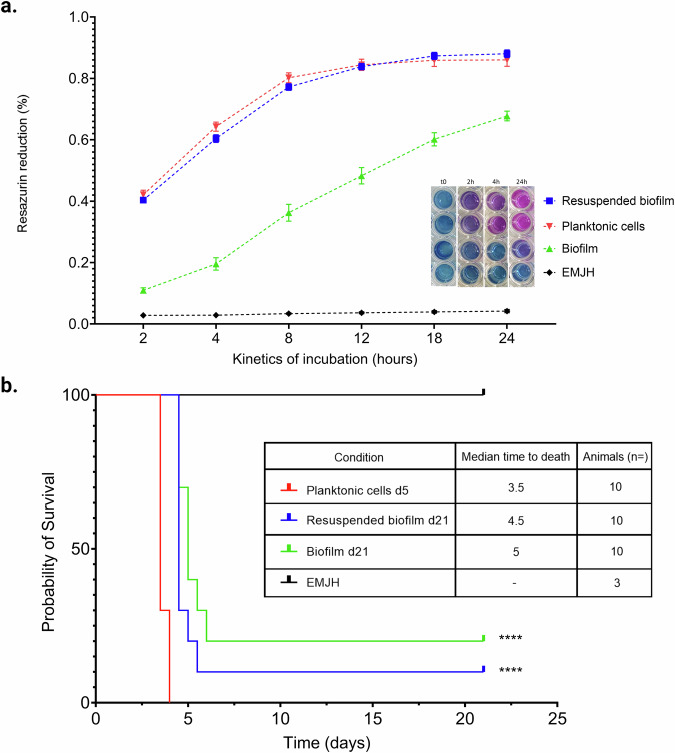
Metabolic activity and virulence of *Leptospira* forming biofilm. **a** The graph represents the metabolic activity as measured by the Resazurin reduction assay for planktonic cells (red), biofilm-associated cells (green), resuspended biofilm cells (blue), and EMJH medium control (black) over 24 hours. Data points show the mean of resazurin reduction to resorufin, indicative of cellular reducing activities, expressed in arbitrary units (a.u.) of absorbance, with error bars representing standard deviation. The plateau in planktonic cells denotes a strong metabolic activity, while the biofilm-forming bacteria demonstrate minimal resazurin reduction. Resuspended biofilm cells rapidly regain metabolic activity comparable to planktonic cells. The inset shows the evolution of the Resazurin assay over time under different conditions. **b** Kaplan–Meier survival curves depicting the time-dependent mortality of hamsters injected with wild-type planktonic bacteria (red line), biofilm forming bacteria (green line), and bacteria resuspended after biofilm disruption (blue line), compared to a control group (EMJH medium—black line). Statistical significance between groups compared to the planktonic condition is denoted by asterisks, where **** denotes *p* < 0.001 after a log-rank (Mantel–Cox) test. The inset table recapitulates the median survival times for each experimental group.

We initially assessed the bacterial reducing activities, a viability proxy, after three weeks in culture using the resazurin reduction assay. In planktonic cells from the exponential growth phase (5-day culture), the rapid conversion of resazurin into resorufin indicated a maintained metabolic activity (Fig. 8 a). Conversely, biofilm-forming bacteria showed a markedly slower resazurin reduction rate. While planktonic cells showed maximum activity within 12 h, biofilm bacteria did not reach similar levels even after 24 h, reflecting a decreased metabolic activity consistent with our transcriptomic findings.

Remarkably, the resuspension of biofilm bacteria almost instantly restored their metabolic activities, equating to planktonic levels within 2 h. This rapid recovery suggests that *Leptospira* in biofilms can rapidly resume high metabolic activity upon biofilm disruption.

We further investigated the infectious capability of biofilm-forming *Leptospira* in a hamster model of acute leptospirosis (Fig. 8b). Hamsters infected with biofilm bacteria had a significantly longer median time to death (5 days) compared to those infected with virulent planktonic bacteria (3.5 days), with two out of ten animals recovering from biofilm-bacterial infection. In contrast, infection with biofilm-resuspended bacteria resulted in a reduced median time to death (4.5 days), with nine out of ten animals succumbing to the infection. These results suggest that while biofilm-forming bacteria exhibit a reduced virulent phenotype than wild-type planktonic bacteria, they retain their infectivity, which is restored when resuspended prior to animal challenge.

To understand these phenotypic changes, we examined the transcriptional regulation of the recently identified virulence-associated genes set under our condition of late biofilm formation. Among the 53 genes reported by Giraud-Gatineau et al.^57^, 16 were significantly modulated. Genes LIMLP_15115, LIMLP_00490, and LIMLP_11105, encoding the molecular chaperone DnaK and two lipoproteins respectively, were upregulated (FC 3.9, 3.7, and 2.2) (Supplementary Table 8). Conversely, genes *flaA1* (LIMLP_13775), *orfC* (LIMLP_13750), *glnA* (LIMLP_11985), *rlpA* (LIMLP_00285), *rsbU* (LIMLP_18140), *lipL32* (LIMLP_06600), and seven others showed downregulation, aligning with the lightly lower virulence phenotype observed in biofilm-infected hamsters. These transcriptional regulations, particularly in virulence factors, support the observed decrease in pathogenicity, suggesting that the biofilm state may modulate virulence traits of *L. interrogans*.

## Discussion

*L. interrogans*, possesses enough adaptive mechanisms to navigate its ecological niche and ensure its transmission among mammalian hosts. Survival in the environment is a key aspect of pathogenic *Leptospira* ecology and a determinant of disease transmission. Biofilm formation, a common survival strategy employed by prokaryotes, affords *Leptospira* an advantage, particularly when faced with harsh external conditions^10,58,59^. These structures not only provide refuge to the bacteria, enhancing persistence, but also facilitate the spread of infectious diseases. Thorough bulk RNA sequencing, we delved into the transcriptional reprogramming that *L. interrogans* undergoes during late biofilm state. The interpretative scope of our study is constrained by the *Leptospira* genome’s partial annotation, with a third of its genes yet to be functionally characterized, directing our focus primarily on the two-thirds that are annotated. Nevertheless, our findings shed light on substantial transformations characteristic of the late biofilm lifestyle, identifying putative key genes involved in these adaptations.

Previous investigations into transcriptional changes during biofilm formation in *Leptospira biflexa*, a saprophytic species thriving in the environment, have highlighted alterations in genes and regulatory networks associated with cell growth, metabolism and DNA regulation^60^. While these findings align with aspects of our study, our results also unveiled the presence of proteotoxic and oxidative stress responses, which contrast with the findings in *L. biflexa*. A closer examination of common signatures revealed that only 10.6 to 15.5% of orthologous genes were significantly modulated in both datasets. This percentage further diminished to 4.2 to 10.2% when considering orthologous genes with matching regulation patterns (Supplementary Table 9). These divergences could be ascribed to differences in experimental setups, notably the duration of biofilm observation (up to 5 days with *L. biflexa* versus 21 days in our study). Despite the high replication rates of *L. biflexa* as compared to those of *L. interrogans*, the discrepancy in time frames remains significant.

Regarding genes involved in cyclic di-GMP metabolism, known for their role in the transition from motile, planktonic states to biofilm formation and vice versa, our observations suggest a trend towards the upregulation of phosphodiesterases and the downregulation of diguanylate cyclases in late biofilms. This pattern may indicate a strategic reduction in c-di-GMP levels, which may prepare the bacteria for a transition back to a planktonic state or enhance their ability to colonize new surfaces. Despite these changes, the balance of c-di-GMP metabolism appears complex, with many genes showing no significant modulation, possibly attributed to the late-stage biofilm conditions sampled in this study, as the significant activities of these genes are likely to be more pronounced during the earlier stages of biofilm formation.

This study reveals the downregulation of genes involved in motility, chemotaxis, energy production, and metabolism within mature biofilms, suggesting a strategic shift towards conservation of resources and a reduced need for motility in this collective living state.

However, two genes related to motility, *fliH* (LIMLP_06770) and *fliI* (LIMLP_06785), exhibited upregulation within the biofilm matrix. In *B. burgdorferi*, another spirochete, transposon mutants of these genes have been reported to have a greatly reduced motility, division defects and non-infectivity in mice^61^. The upregulation of *fliH* and *fliI* in biofilm-forming *Leptospira* may represent a strategic mechanism to retain infectivity or to ensure a swift reactivation of flagellar assembly upon dispersion from the biofilm. This finding aligns with the observed rapid transition from a quiescent state in the biofilm to immediate metabolic and virulent activity, suggesting an underlying readiness to regain motility when environmental conditions become conducive.

We also noticed a downregulation of the three genes *flaA2* (LIMLP_13780), *fcpA* (LIMLP_01630) and *fcpB* (LIMLP_09180) for which *Leptospira* knockout mutants lost their translational motility^62–64^. Additionally, mutants deficient in FcpA and FlaA2 displayed a diminished capacity to induce disease in a hamster model^62,64^. FcpA and FcpB were recently shown to decorate the core filament on the convex side and interact with FlaB to facilitate filament curvature, which is essential for the formation of flagellar supercoiling, thereby contributing to bacterial motility^65^. These gene expression changes strongly correlate with the observed phenotypic change of reduced motility and virulence in *Leptospira* biofilms^10^.

Consistent with a reduced metabolism, we evidenced downregulation of cell division. The *ftsW* gene (LIMLP_09265), vital for FtsI recruitment and peptidoglycan synthesis within the divisome in *E. coli*^66,67^, was downregulated along with two *ftsI* genes (LIMLP_06170, LIMLP_15095). Although the extent of downregulation observed is not drastic, it is nonetheless indicative of substantial changes in these pathways. Taken together, these findings suggest that in the late stages of biofilm development, the bacterial population shifts away from active cell division, opting instead to repress functions related to growth and division. This adaptive response likely reflects a transition to a state where maintenance and stability of the biofilm structure take precedence over bacteria proliferation. Moreover, LbbD (the orthologous gene of LIMLP_13155) and MreB have been identified as crucial components of the *L. biflexa* cytoskeleton, contributing to the maintenance of its shape. Especially, a knockout mutation in the *lbbD* gene resulted in *L. biflexa* displaying a distinct compressed helical morphology, reduced motility, and loss of MreB function was also associated with morphological abnormalities^31,68^. Despite the downregulation of these genes, scanning electron microscopy observations of pathogenic leptospires in biofilm did not evidence similar structural alterations^10^.

In long-term cultures, a typical observation is the marked reduction in metabolic activities, which correlates with diminished requirements for energy production. However, we described an intriguing activation of the methylglyoxal pathway, an auxiliary route branching from glycolysis that processes methylglyoxal (MG) into pyruvate. Notably, the gene coding for methylglyoxal synthase (LIMLP_03805), which converts dihydroxyacetone phosphate (DHAP) to MG, was markedly upregulated. This upregulation suggests a rerouting of DHAP, potentially derived from glycerol catabolism—abundant in EMJH medium—towards MG production. This trend would indicate a preference for glycerate degradation pathways, possibly as a route for DHAP production. The downside of this pathway is the generation of MG itself—a highly reactive dicarbonyl molecule well-known for modifying proteins and forming advanced glycation end products (AGEs), which are generally considered toxic^69^.

Upon its formation, MG can react spontaneously with glutathione to yield hemithiolacetal (HTA), which is subsequently metabolized to D-lactate through the glyoxalase system^70,71^. This pathway’s importance was highlighted by the significant upregulation of glyoxalase II enzymes (LIMLP_08945 and LIMLP_01865), indicating an active conversion of S-lactoylglutathione (SLG) to L-lactate and simultaneous regeneration of glutathione.

One can hypothesize that these transcriptional patterns suggest a strategic redirection of carbon flux from glycerol metabolism towards lactate production, with the concomitant benefit of glutathione regeneration, imperative for maintaining redox balance. The production of methylglyoxal by cells, while paradoxical, may play a crucial role in energy production, assuming a functional MG detoxification system is in place.

The findings that many genes related to a general stress response were markedly upregulated upon biofilm formation strongly suggest that pathogenic *Leptospira* are exposed to a wide range of cellular damage in late biofilm condition, including those caused by oxidants and metals. For instance, key players include peroxiredoxin (*prx*) and thioredoxin (*trxA*, *trxB*) systems, alongside the glutaredoxin system. This response is associated with increased expression of genes involved in glutathione biosynthesis (*gshA*, *gshB*), collectively illustrating *Leptospira* adaptation against oxidative stress within the biofilm. Additionally, genes responsible for proper folding of periplasmic proteins (*dsbD* and dsbH-like) and glutathione S-transferase were upregulated, indicating mechanisms aimed at preserving protein integrity through glutathionylation and thiol-disulfide equilibrium modulation. Notably, there was a significant increase in the expression of NADPH-dependent FMN reductase (LIMLP_16870) and quinolinate synthase A (LIMLP_02615), highlighting the critical role of NADPH recycling. These genes support NADPH regeneration, essential for reducing oxidized proteins and detoxifying reactive oxygen species. This ensures a micro-environment that remains conducive to maintaining the structural and functional integrity of proteins, effectively countering oxidative stress and safeguarding cellular viability.

Moreover, the observed increase in cytochrome C maturation genes provides additional information on the pathogen’s oxidative stress management. Given that cytochrome C biogenesis is a periplasmic pathway involving thiol oxidoreductases and necessitates the reduction of cysteine residues for heme B binding, one can envision that a potent oxidant, could interfere with cysteines and/or the reduction system of the cytochrome C biogenesis pathway. Yet, the upregulation of these genes may reflect a preventive defense mechanism, safeguarding this crucial cellular process against oxidative damage.

Our data also evidence a complex regulatory scheme governing sulfur production pathways, Fe–S cluster assembly, and iron homeostasis, indicating a cellular effort to protect Fe–S cluster integrity. This coordinated response is vital for preserving the function of Fe–S proteins, crucial for cellular energy production, maintaining redox balance, and defending against oxidative stress. The synchronized regulation of genes involved in sulfur metabolism, Fe–S cluster assembly, and iron homeostasis underscores a comprehensive approach to maintaining vital cellular functions during oxidative stress associated with biofilm formation.

Interestingly, only a subset of genes exhibiting modulation when *L. interrogans* were exposed to H_2_O_2_^46^ were deregulated in biofilms. Indeed, expression of key genes associated with H_2_O_2_ reduction—such as *ccp* (cytochrome C peroxidase), *ahpC* (peroxiredoxin), and *katE* (catalase)—remained largely unaltered in our biofilm model. This suggests that the oxidative stress response in late biofilms is distinct from that induced by H_2_O_2_. Nonetheless, it has been shown that oxidative stress can influence biofilm formation in several bacterial species^72,73^. Specifically, genes involved in detoxifying reactive oxygen species, such as *ahpC*, *tpX*, *sodC*, glutathione peroxidase, or genes involved in protein refolding, are often upregulated in biofilms^74–78^. Although pathogenic leptospires do not possess all the previously mentioned genes to manage oxidative stress, we have shown an upregulation of key genes related to oxidative stress.

One important finding of our study is the significant upregulation of the *csoR*/*copZA* operon, putatively integral to the copper ion stress response. This upregulation suggests a potential accumulation of copper that would need to be experimentally demonstrated. The concentration of copper sulfate present in EMJH medium would yield to only 1.2 µM of free copper, a level considered non-toxic to *L. interrogans*^79^. Biofilm formed in a copper-free EMJH medium also showed a significant up-regulation of the *csoR*/*copZA* copper homeostasis system (data not shown) confirming that this 1.2 µM concentration is not responsible for the upregulation observed. Release of copper from deceased cells embedded within the biofilm might contribute to local elevated copper concentrations. Excessive copper can induce protein misfolding/aggregation through the catalysis of improper disulfide bonds^80^ and damage to iron-sulfur clusters^81^. The increased expression of molecular chaperones, iron-sulfur cluster biosynthesis pathways, and redox-related machineries (thioredoxin, glutaredoxin, and glutathione) within the late biofilm could suggest a copper or metal-associated stress. The overexpression of *acrB* and *tolC*, encoding a putative efflux pump, in our dataset also suggests accumulation of copper (or other metal) that would need to be exported from the bacteria. Interestingly, the AcrA/B-TolC system is also known to play a role in biofilm formation in other bacteria^53,82^.

Transcriptomic analysis of *Staphylococcus aureus* under copper stress revealed upregulation of copper homeostasis mechanisms, enhanced oxidative stress resistance and misfolded protein response, and the downregulation of several transporters and global regulators as we described in this study^83^. This suggests a close link between copper stress response and biofilm adaptation. Moreover, in *Streptococcus mutans*, copper addition and manipulation of copper homeostasis operon (*copYAZ*) significantly influenced biofilm formation and stress tolerance^84^. In *Alteromonas macleodii*, copper-unadapted cells subjected to copper stress showed higher biofilm levels^85^. Similarly, *Acinetobacter baumannii* biofilms exhibit increased copper resistance, although copper treatment still reduced bacterial populations within biofilms^86^. These findings underline the relationship between copper stress and biofilm formation in bacterial infections, underscoring the need to explore copper-mediated mechanisms in the context of biofilm-associated infections.

Alternatively, it is conceivable that the *Leptospira* CsoR may also respond to other metals or signals. This could also explain the activation of the copper response operon in an environment where copper is scarce or absent. While a causal link between the copper and oxidative stress responses remains to be proven in *Leptospira* biofilms, one can speculate that the oxidative stress response observed in biofilms was triggered by a copper (or other metal) stress.

These findings highlight the strategies employed by *L. interrogans* to maintain cellular homeostasis and viability amidst the oxidative stress encountered in the biofilm. This resilience is critical, potentially facilitating survival in adverse ecological conditions *via* biofilm formation and the use of observed adaptive mechanisms, while ensuring the continuity of its pathogenic lifecycle.

We extended our investigation to assess the physiological state of bacteria within the biofilm, questioning their viability amidst diminished metabolic activity and its implications for pathogenicity. Viability test based on resazurin reduction indicated that leptospires in biofilms exhibited lower activity compared to their planktonic counterparts in the exponential phase. However whether this lower reducing activity is due to fewer viable bacteria within the biofilm cannot be determined from the current results^87^. Additional experiments suggest that although leptospires in late biofilm form are viable and can be cultured, their ability to form colonies is limited (data not shown). Remarkably, mechanical resuspension restored the metabolic activity of 21-day-old cultured leptospires to levels comparable to those observed in planktonic leptospires, despite the previously described regulatory changes and without the introduction of fresh media. This suggests that resuspension alone may serve as a sufficient trigger to reactivate *L. interrogans* metabolism including virulence. This rapid recovery is of prime importance, suggesting that *Leptospira* within biofilms can quickly revert to high metabolic activity following biofilm disruption, as it could occur upon heavy rainfall event in the environment or upon micturition in chronic carriers.

Perhaps more importantly, we demonstrated here that biofilm-associated *Leptospira* retain their pathogenicity. Indeed, leptospires from resuspended biofilms showed a trend towards increased virulence compared to their biofilm counterparts. This observation could underpin the link between leptospirosis incidence and heavy rainfall events^88^, as leptospires dispersed by water might revert to a planktonic state, enabling them to colonize new niches or hosts. Such a mechanism aligns with the pattern of human leptospirosis cases following significant meteorological events, attributed to the mobilization of pathogenic *Leptospira* in surface waters, likely deriving from biofilms. However, given that the infections were administered intraperitoneally at high doses, these findings should be interpreted with caution. In natural environments, infections are thought to result from minimal bacterial exposure, and biofilms, typically sessile, seldom directly interact with potential hosts.

In conclusion, this study sheds light on the complex transcriptional adjustments *L. interrogans* undergoes during biofilm lifestyle over extended periods. Our findings strongly suggest a strategic reduction in genes associated with motility, energy production, and metabolism, contrasted by an increase in genes pivotal for stress response, defense against metal stress, and redox equilibrium. This adaptive shift hints at a sophisticated defense against oxidative stress, potentially linked to metals, underscored by the notable elevation of the *csoR*/*copZA* operon expression. These insights not only deepen our comprehension of the survival and persistence mechanisms within *Leptospira* biofilms but also challenge the conventional belief that biofilm structure diminishes virulence^14–16^. On the contrary, we demonstrate that *Leptospira* forming biofilm maintain virulence, supporting the vital role biofilms might play in the pathogen’s lifecycle from environmental survival to host invasion. This investigation enriches our understanding of *Leptospira*’s biofilm dynamics, revealing its effective adaptation between environmental resilience and the maintenance of infectivity.

## Methods

### *L. interrogans* cultures and biofilm formation

*L. interrogans* serovar Manilae strain L495 was used for these experiments. Bacteria were cultured in Ellinghausen, McCullough, Johnson, and Harris (EMJH) liquid medium at 30 °C without shaking up to 5 passages before performing biofilm experiments. Bacteria were grown to exponential phase and counted using a Petroff-Hausser cell-counting chamber (Hausser Scientific Company, Horsham, PA, USA) under dark-field microscopy. Culture was diluted in EMJH to a final concentration of 10^6^ bacteria mL^−1^. Under sterile conditions, 40 mL of diluted culture were dispensed into a T25 flask (Easy Flask Nunc, Dutscher). For quantitative assays, 4 replicate flasks were used. Flasks were then incubated vertically at 30 °C for 3 weeks under static conditions without changing the culture medium. At 5 days, 20 mL of planktonic cells in the liquid phase (*i.e*. the non-adhering biofilm-forming bacteria) were collected for RNA extraction. After 3 weeks of culture, the entire supernatant (40 mL of the liquid phase) containing planktonic bacteria was removed. The biofilm-forming bacteria were then resuspended in 10 mL of fresh media and transferred to a new 15 mL Falcon tube prior to quickly proceeding with RNA extraction.

### Biofilm formation quantification and microscope image acquisition

Bacteria were grown to exponential phase and diluted in EMJH to a final concentration of 1 × 10^6^ bacteria.mL^−1^. Then, 200 µL of the bacterial suspension were distributed in wells of a 96-well plates and incubated under static conditions at 30 °C until the desired timepoint (days 3, 5, 7, 10, 12, 14, 17, 21). For quantitative assays, the content of six wells was resuspended (total), the liquid phase from six wells was carefully collected and placed in an empty well (liquid phase), and the remaining biofilm resuspended in a total volume of 200 µL with EMJH medium (biofilm) at each time point. Optical density measurement at 405 nm were obtained using a spectrophotometer (Multiskan FC, Thermos Scientific) to determine the bacterial density.

At each time point, phase contrast images of biofilm were also captured using an upright Leica DM4000 B microscope (Leica microsystems, Mannheim, Germany) equipped with a 5X lens (HCX PL FLUOTAR 5×/0,15, 12 mm working distance). Pictures of six replicate wells per independent experiment were acquired and further analyzed. The surface area of the biofilm was measured using the ImageJ software^89^. Prior quantification, the outer part of the image affected by spherical aberrations and field curvature artefacts was cropped. The 2D grayscale image was then binarized and analyzed using the “Analyze Particles” function. The biofilm score was then expressed as a percentage of surface occupied by the biofilm.

### Crystal violet (CV) staining

Biofilm was grown by adding 2 × 10^5^ leptospires of an exponential phase culture on a sterile polycarbonate hydrophilic membrane (ipPORE^TM^ track Etched Membrane filters, it4ip Belgium), and placing it on a 1.2% agar EMJH medium at 30 °C. After 21 days of incubation, membranes were transferred into a 12-well plate and stained with 200 µL of 0,1 % CV solution. After 10 minutes of incubation the staining solution was removed, membranes were rinsed 3 times with 1 mL PBS and dried overnight. The stain was then released by adding 200 µL of a 50/50 (vol/vol) ethanol/ glacial acetic acid solution and the absorbance was measured at 570 nm using a spectrophotometer (Multiskan FC, Thermos Scientific).

### RNA extraction and sequencing

Total RNA was extracted from biofilm or planktonic cells as previously described for leptospires^90^. Briefly, cells were collected by centrifugation for 15 min at 3000 g at 4°C and resuspended in 1 mL TRIzol^TM^ (15596026, Invitrogen, USA) before adding 260 µL of chloroform. After mixing and a ten-minute incubation, the samples were centrifuged for 15 min at 12000 g at 4 °C to collect the aqueous phase containing RNA. Precipitation and washing were performed with 600 µL isopropanol, centrifugation for 10 min at 12000 g at 4 °C, removal of the supernatant and addition of 1 mL of 75% ethanol. After a last centrifugation, the RNA was air-dried and resuspended in 40 µL of RNase-free water. Samples were treated with TURBO^TM^ DNase (AM2238, ThermoFischer Scientific) and RNA concentration was measured using a Nanodrop^TM^ (ND-2000 Spectrophotometer, Thermofischer Scientific).

RNA libraries were built using the Illumina Stranded total RNA library with Ribo-Zero Plus Preparation Kit (Illumina, USA) with an addition of custom probes targeting *L. interrogans* serovar Manilae ribosome sequences (DNAScript, FR). Sequencing was performed on a NextSeq500 (Illumina, USA) to obtain 75 base single-end reads for a target of an average 10 M reads per sample.

The RNA-seq analysis was performed with Sequana pipeline (version 0.13.0)^91^ built on top of Snakemake 6.1.1^92^. Briefly, reads were trimmed from adapters using Fastp 0.20.1 with default settings^93^ and then mapped to the *L. interrogans* serovar Manilae UP-MMC-NIID LP genome assembly accession numbers NZ_CP011931.1, NZ_CP011932.1 and NZ_CP011933.1 from NCBI, using Bowtie2 (version 2.4.2)^94^. FeatureCounts 2.0.1^95^ were used to produce the transcript count matrix, assigning reads to features using corresponding annotation from NCBI guided by strand-specific information. Quality control statistics were summarized using MultiQC 1.10.1^96^.

Eight libraries were constructed, including 4 biological replicates per condition. The *L. interrogans* serovar Manilae UP-MMC-NIID LP genome contains 3651 predicted genes distributed within three replicons: chromosome I (CI), chromosome II (CII) and a 70 kb plasmid. All of them showed transcriptional activity in our dataset, although 178 genes did not match the LMANV2_v2 annotation. Twenty genes were removed from the analysis because their alignment on the last version of the complete genome did not correspond to any identified gene in *L. interrogans* Manilae L495 genome in MaGe (n = 17), or they coded for tRNAs (n = 3) (Supplementary Table 10).

### Statistical analysis of differentially expressed genes

Statistical analysis was performed with DESeq2 R package (1.24.0^97^) to identify differentially expressed genes between the bacteria within the late biofilm and the planktonic cells. The analysis included (i) data description and quality control, (ii) data normalization and exploration, (iii) and testing for differential expression for each gene between the two selected conditions. Data were normalized according to the DESeq2 model. The variability of the data was explored by performing hierarchical clustering and principal component analysis (PCA) of the entire sample set after the counts were transformed using a variance stabilizing transformation. Hierarchical clustering was performed using the Euclidian distance and the Ward criterion for agglomeration. PCA was performed using the DESeq2 R package to explore the structure and clustering of the samples (Supplementary Fig. 1A). Differential expression results were corrected for multiple testing using the Benjamini-Hochberg method using a false discovery rate [FDR] of <0.05). The log_2_FC values were converted to fold changes, applying the formulas ${FC}={2}^{X}$ and ${FC}=-\frac{1}{{2}^{X}}$ for positive and negative log_2_FC (X) values.

### Functional annotation and enrichment analysis

*L. interrogans* genes were mapped to 98 KEGG biological pathways using the KEGGREST (v 1.36.3) R package and *L. interrogans* serovar Lai 56601 KEGG’s annotation.

*L. interrogans* genes were also mapped to COG annotations retrieved from MaGe website resulting in 2158 COG terms belonging to 22 COG classes and 4 COG processes^20^.

Functional enrichment analysis of KEGG pathways and COG terms was performed using the Camera (competitive gene set test accounting for inter-gene correlation) method from the limma R package (v 3.52.2)^98^.

### Confirmation of differentially expressed genes by quantitative RT-PCR

cDNA synthesis was performed on the RNA samples from this study with the Transcriptor First Strand cDNA Synthesis Kit (Roche) according to the manufacturer’s recommendations. Quantitative PCR were conducted with the LightCycler 480 SYBR Green I Master mix (Roche Applied Science, Auckland, New Zealand) on a LightCycler 480 platform. For all genes, cycling conditions were as follows: 10 min at 95 °C and 50 cycles of 5 s at 95 °C, 5 s at 60 °C plus 15 s at 72 °C. Gene expression was measured with primers listed in supplementary table 11 using 16S rRNA as a reference gene for normalization in this study^99^.

### Cell viability and metabolic activity

*L. interrogans* was cultivated as described before in 96-well plates. After 3 weeks of culture, 200 µL of resuspended biofilm, biofilm, and planktonic cells (5 days of culture) were carefully loaded in a new 96 well plate. Twenty microlitres of resazurin (Alamar Blue Assay, ThermoFisher Scientific) were added and cells were further incubated for 30 h. Viability could be assessed by the reduction of blue resazurin into pink resorufin as explained elsewhere^87^. The chromogenic shift was measured by absorbance at 570 nm/620 nm with a microplate reader (Multiskan FC, Thermos Scientific). Negative controls without bacteria were included. Relative metabolic activity was calculated as recommended by the manufacturer and expressed in percentage. Experiments were repeated at least 4 times.

### Animal infection

After quantification of the number of leptospires in a biofilm control well, 2 × 10^8^ leptospires were inoculated intraperitoneally into 7–8-week-old golden Syrian hamsters (males and females). Conditions were as follows: 5 days old planktonic leptospires, 21 days old biofilm aggregates and 21 days old biofilms aggregates resuspended just prior to injection. When injecting biofilm, the aggregates were carefully harvested using the syringe alone and then injected with a 21 G needle. Of note, tests were carried out beforehand to ensure that the aggregates were not dissolved by the push through the needle during injection (Supplementary Fig. 5). After injection, hamsters were monitored twice daily for up to 21 days for clinical signs of leptospirosis and subsequently euthanized by carbon dioxide inhalation. During this monitoring, animals showing signs of suffering (ruffled fur, prostration, poor response to stimulation) were euthanized, and the moment of euthanasia was taken as the moment of death. The experiment was performed as three biological replications with different cultures and at different dates, infecting three to four hamsters each time, leading to a total of 10 hamsters per condition. For each replication, 1 hamster was injected with EMJH medium as a negative control. All experiments were conducted according to the institutional guidelines of the Animal Care and Use Committees of the Institut Pasteur and followed European (EU directive 2010/63) regulations on Animal Welfare and with Public Health Service recommendations.

## Supplementary information

Supplementary information

nr-reporting-summary_Davignon

## Data Availability

RNA-Seq data generated in this study are available in the NCBI-GEO with the accession number GSE271193.
