# Supplementary material for: Leptospira interrogans biofilm transcriptome highlights adaption to starvation and general stress while maintaining virulence
Source: NPJ Biofilms Microbiomes. 2024 Sep 30;10:95. doi: 10.1038/s41522-024-00570-0 (PMC11442865; doi:10.1038/s41522-024-00570-0)
Supplement: Supplementary file 1 — Supplementary information [file 41522_2024_570_MOESM1_ESM.pdf]

Supplementary information

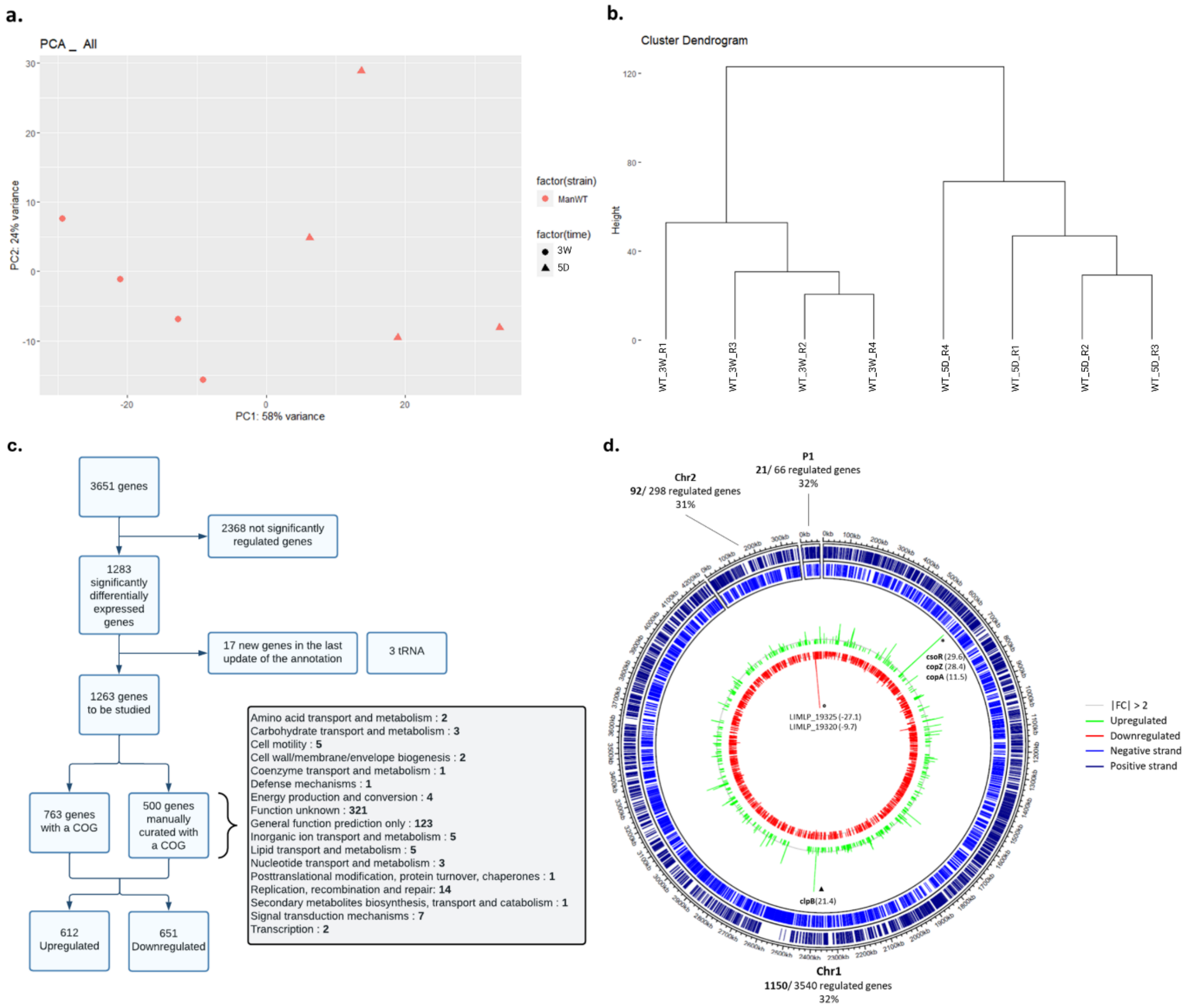

Supplementary figure 1: Dataset Description

(a-b) PCA analysis and cluster dendrogram of the 4 replicates showing a clear separation of the two conditions at 3 weeks and 5 days. **(c)** Diagram summarizing the main different steps of the analysis. 20 genes were filtered out from the study because they either did not match the reference genome or were encoded by tRNAs. 500 genes were further manually annotated with a Cluster of Orthologous Groups (COGs) function to improve the functional annotation. Among them, 10 transposases were added to the Replication, Recombination and Repair category. **(d)** Circular representation of the distribution of deregulations in the *L. interrogans* genome. Positive and negative strand coding sequences are shown in dark and light blue, respectively. Statistically

significantly ($p_{adj}<0.05$) overexpressed and underexpressed genes are shown in green and red, respectively. The grey line indicates a |FC|>2. The modulated genes were evenly distributed on both bacterial chromosomes and on the *L. interrogans* plasmid. The 3 genes or gene groups with the highest CF were annotated on the figure.

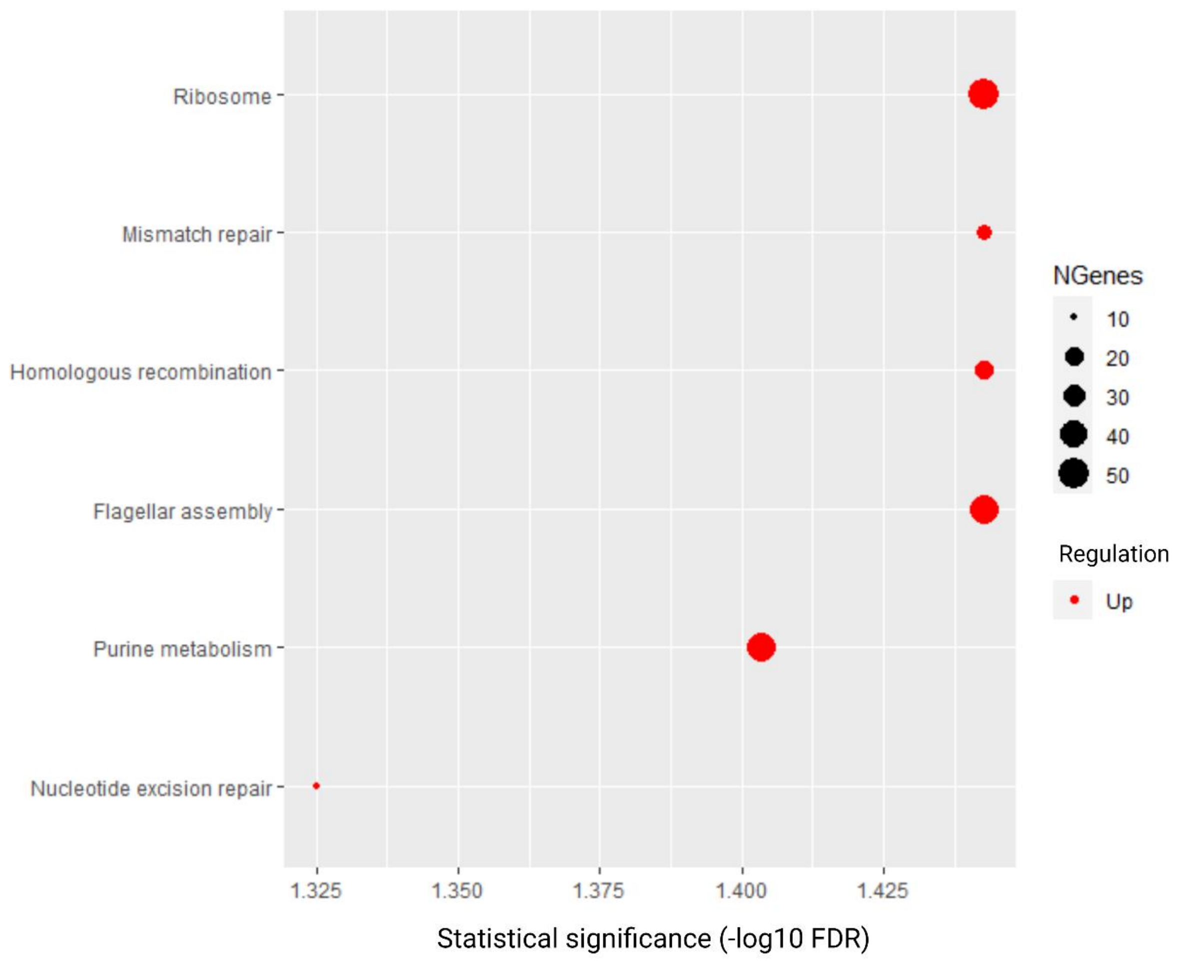

Supplementary figure 2: KEGG Regulations within a Late Biofilm

Bubble graph representing statistically enriched KEGG pathways in the dataset resulting from the comparison between a day 21 biofilm and day 5 planktonic cells. The analysis was carried out on all the significantly differentially expressed genes and shows regulation of 6 major pathways. The Y axis shows the list of KEGG pathways, while the X axis represents the statistical significance. Bubble size is proportional of the number of genes belonging to each pathway, while bubble color shows the direction of over-representation.

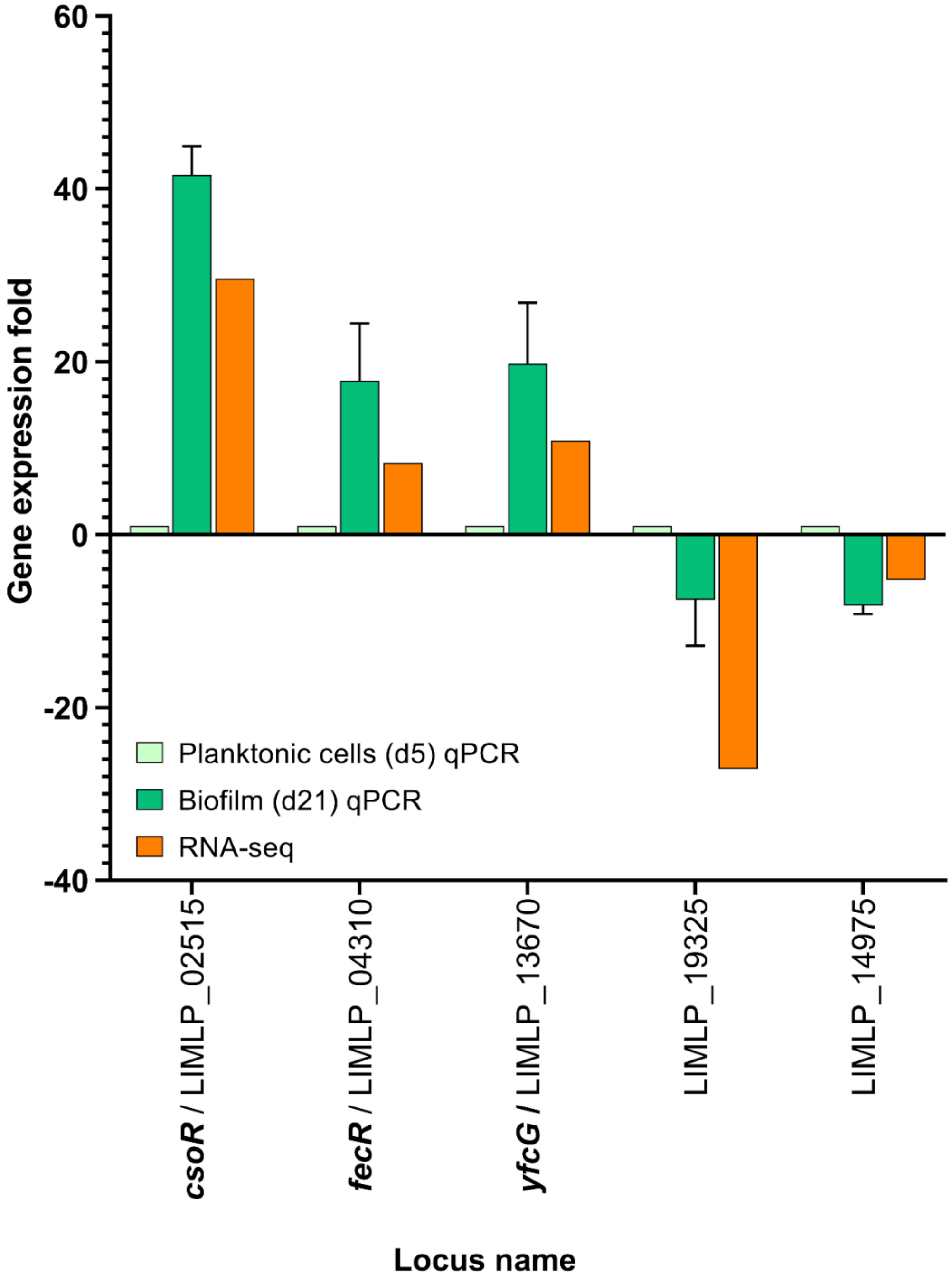

Supplementary figure 3: Quantitative RT-PCR for RNA-seq validation

Gene expression was measured by RT-qPCR reactions to confirm expression levels, ensuring the accuracy and reliability of the data presented. Gene expression in *L. interrogans* biofilm (day 21, dark green bars) was compared to that in planktonic cells (day 5, light green bars). Relative fold

changes from RNA-seq are represented for each gene with the orange bars. For each gene, bar graphs represent the mean ± SEM.

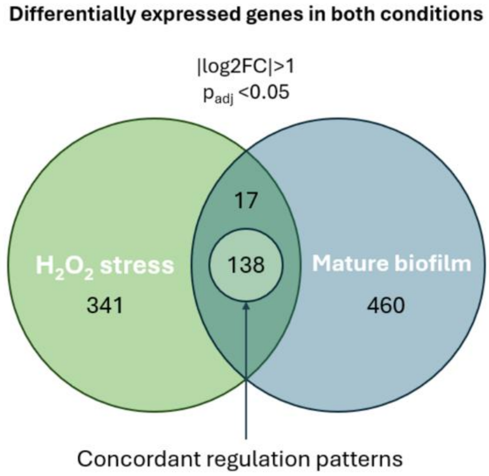

Supplementary figure 4: Venn Diagram of the Comparison between Late Biofilm and H_2O_2 Response.

Venn diagram illustrating the genes differentially expressed in *L. interrogans* L495 upon exposure to 1 mM H_2O_2 for one hour (as determined in ref (46)) and upon biofilm formation (as determined in this study) (p_{adj}<0.05 and |log2FC|>1). Of the 496 genes modulated by H_2O_2, 155 were common to both conditions and 138 showed a concordant pattern of regulation.

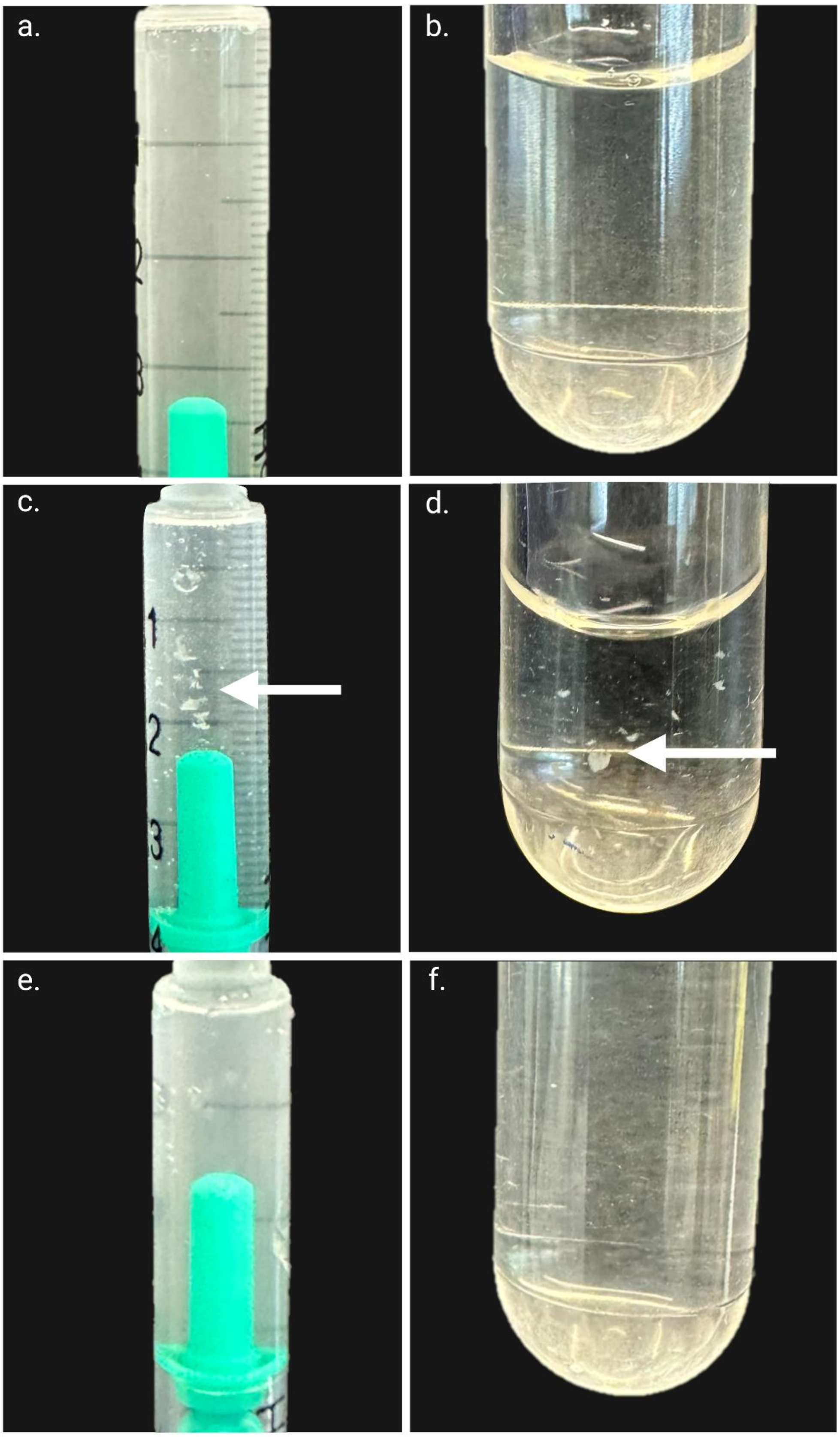
a.
b.
c.
d.
e.
f.

Supplementary figure 5: Preparation of inocula for virulence tests on hamsters.

Leptospires were injected either in planktonic form (day 5) (a,b), in biofilm form (day 21) (c,d) or in resuspended biofilm form (day 21) (e,f). The figures illustrate these conditions in the syringe (a, c, e) and after passage through the needle (b, d, f). Biofilm aggregates are shown by white arrows.

Supplementary table 1: Differentially expressed genes within a mature biofilm (padj0.05)

Index	Locus	log2FoldChange	Fold Change	padj
LIMLP_RS00010	LIMLP_00010	0,46	1,4	3,3,E-02
LIMLP_RS00020	LIMLP_00020	2,29	4,9	1,9,E-10
LIMLP_RS00025	LIMLP_00025	0,50	1,4	1,3,E-02
LIMLP_RS00030	LIMLP_00030	0,38	1,3	1,1,E-02
LIMLP_RS00045	LIMLP_00045	-1,53	-2,9	2,4,E-06
LIMLP_RS00055	LIMLP_00055	-0,83	-1,8	1,7,E-03
LIMLP_RS00075	LIMLP_00075	-1,64	-3,1	5,3,E-09
LIMLP_RS00090	LIMLP_00090	-0,75	-1,7	3,2,E-03
LIMLP_RS00095	LIMLP_00095	-0,63	-1,5	4,1,E-03
LIMLP_RS00100	LIMLP_00100	1,51	2,9	6,4,E-09
LIMLP_RS00125	LIMLP_00125	-0,77	-1,7	1,6,E-03
LIMLP_RS00130	LIMLP_00130	-0,98	-2,0	8,5,E-08
LIMLP_RS00135	LIMLP_00135	-0,53	-1,4	2,6,E-02
LIMLP_RS00140	LIMLP_00140	-0,64	-1,6	1,9,E-02
LIMLP_RS00145	LIMLP_00145	-1,24	-2,4	2,7,E-08
LIMLP_RS00150	LIMLP_00150	-0,36	-1,3	4,9,E-02
LIMLP_RS00190	LIMLP_00190	-1,02	-2,0	2,2,E-05
LIMLP_RS00195	LIMLP_00195	-1,16	-2,2	9,5,E-06
LIMLP_RS00215	LIMLP_00215	0,65	1,6	4,7,E-02
LIMLP_RS00235	LIMLP_00235	-0,82	-1,8	2,7,E-04
LIMLP_RS00240	LIMLP_00240	-0,68	-1,6	3,3,E-02
LIMLP_RS00245	LIMLP_00245	-1,10	-2,1	1,5,E-02
LIMLP_RS00265	LIMLP_00265	-1,37	-2,6	6,3,E-10
LIMLP_RS00285	LIMLP_00285	-1,13	-2,2	1,4,E-06
LIMLP_RS00290	LIMLP_00290	1,79	3,5	7,5,E-08
LIMLP_RS00320	LIMLP_00320	-0,37	-1,3	3,4,E-02
LIMLP_RS00325	LIMLP_00325	-0,80	-1,7	1,1,E-02
LIMLP_RS00330	LIMLP_00330	-0,42	-1,3	2,4,E-02
LIMLP_RS00340	LIMLP_00340	-0,88	-1,8	4,7,E-02
LIMLP_RS00350	LIMLP_00350	-1,05	-2,1	1,9,E-02
LIMLP_RS00370	LIMLP_00370	2,00	4,0	4,6,E-03
LIMLP_RS00375	LIMLP_00375	-0,82	-1,8	2,3,E-03
LIMLP_RS00385	LIMLP_00385	-0,51	-1,4	1,2,E-02
LIMLP_RS00400	LIMLP_00400	1,08	2,1	4,5,E-08
LIMLP_RS00405	LIMLP_00405	-0,83	-1,8	1,2,E-04
LIMLP_RS00410	LIMLP_00410	-0,47	-1,4	1,8,E-02
LIMLP_RS00425	LIMLP_00425	1,09	2,1	5,6,E-03
LIMLP_RS00435	LIMLP_00435	1,12	2,2	1,4,E-02
LIMLP_RS00445	LIMLP_00445	-0,77	-1,7	9,7,E-04
LIMLP_RS00475	LIMLP_00475	3,32	10,0	1,7,E-07
LIMLP_RS00480	LIMLP_00480	2,82	7,1	3,0,E-07
LIMLP_RS00485	LIMLP_00485	2,52	5,8	4,1,E-06
LIMLP_RS00490	LIMLP_00490	1,90	3,7	1,5,E-05

LIMLP_RS00495	LIMLP_00495	1,55	2,9	5,5,E-05
LIMLP_RS00500	LIMLP_00500	-0,71	-1,6	1,7,E-02
LIMLP_RS00595	LIMLP_00590	-1,13	-2,2	1,1,E-02
LIMLP_RS00605	LIMLP_00600	-0,35	-1,3	4,5,E-02
LIMLP_RS00610	LIMLP_00605	-0,46	-1,4	3,5,E-02
LIMLP_RS00615	LIMLP_00610	-1,13	-2,2	3,3,E-03
LIMLP_RS00620	LIMLP_00615	-1,11	-2,2	1,6,E-02
LIMLP_RS00680	LIMLP_00675	0,90	1,9	4,4,E-03
LIMLP_RS00700	LIMLP_00695	0,64	1,6	1,2,E-02
LIMLP_RS00715	LIMLP_00710	-0,88	-1,8	2,5,E-05
LIMLP_RS00725	LIMLP_00720	1,10	2,1	8,1,E-03
LIMLP_RS00730	LIMLP_00725	0,98	2,0	2,1,E-02
LIMLP_RS00735	LIMLP_00730	0,57	1,5	1,2,E-02
LIMLP_RS00740	LIMLP_00735	-1,32	-2,5	4,5,E-03
LIMLP_RS00765	LIMLP_00760	0,60	1,5	1,1,E-03
LIMLP_RS00770	LIMLP_00765	1,29	2,4	2,1,E-11
LIMLP_RS00775	LIMLP_00770	1,73	3,3	1,2,E-03
LIMLP_RS00790	LIMLP_00785	0,49	1,4	7,3,E-03
LIMLP_RS00820	LIMLP_00820	-1,15	-2,2	2,2,E-03
LIMLP_RS00825	LIMLP_00825	-1,44	-2,7	2,2,E-06
LIMLP_RS00845	LIMLP_00845	-0,99	-2,0	6,4,E-07
LIMLP_RS23360	LIMLP_00850	2,90	7,5	1,1,E-02
LIMLP_RS00855	LIMLP_00855	2,70	6,5	1,6,E-02
LIMLP_RS00860	LIMLP_00860	2,89	7,4	2,4,E-02
LIMLP_RS00865	LIMLP_00865	3,08	8,4	1,5,E-02
LIMLP_RS00870	LIMLP_00870	3,24	9,4	9,3,E-03
LIMLP_RS00875	LIMLP_00875	3,14	8,8	5,9,E-03
LIMLP_RS00880	LIMLP_00880	2,98	7,9	5,3,E-03
LIMLP_RS00885	LIMLP_00885	2,94	7,7	1,7,E-03
LIMLP_RS23370	LIMLP_00895	1,30	2,5	3,2,E-02
LIMLP_RS00900	LIMLP_00900	1,53	2,9	1,4,E-02
LIMLP_RS00910	LIMLP_00910	1,30	2,5	4,9,E-02
LIMLP_RS00915	LIMLP_00915	1,46	2,7	6,9,E-03
LIMLP_RS00965	LIMLP_00965	1,38	2,6	4,9,E-02
LIMLP_RS00970	LIMLP_00970	1,17	2,2	4,9,E-02
LIMLP_RS01015	LIMLP_01015	-1,36	-2,6	2,6,E-10
LIMLP_RS01025	LIMLP_01025	1,64	3,1	2,1,E-11
LIMLP_RS01030	LIMLP_01030	1,27	2,4	1,7,E-07
LIMLP_RS01045	LIMLP_01045	1,40	2,6	3,2,E-08
LIMLP_RS01050	LIMLP_01050	0,81	1,8	1,4,E-04
LIMLP_RS01075	LIMLP_01075	-0,96	-1,9	2,1,E-03
LIMLP_RS01100	LIMLP_01100	-0,84	-1,8	3,4,E-02
LIMLP_RS01105	LIMLP_01105	-1,21	-2,3	3,3,E-03
LIMLP_RS01110	LIMLP_01110	-1,28	-2,4	5,0,E-04
LIMLP_RS01120	LIMLP_01120	0,84	1,8	6,6,E-03
LIMLP_RS01125	LIMLP_01125	-1,05	-2,1	1,0,E-03

LIMLP_RS01155	LIMLP_01155	-0,39	-1,3	1,7,E-02
LIMLP_RS01160	LIMLP_01160	-0,59	-1,5	4,0,E-02
LIMLP_RS01230	LIMLP_01230	-0,98	-2,0	1,4,E-02
LIMLP_RS01240	LIMLP_01240	1,24	2,4	3,3,E-06
LIMLP_RS01245	LIMLP_01245	1,01	2,0	9,5,E-04
LIMLP_RS01250	LIMLP_01250	0,82	1,8	6,8,E-04
LIMLP_RS01255	LIMLP_01255	-1,35	-2,5	3,2,E-04
LIMLP_RS01280	LIMLP_01280	0,64	1,6	3,6,E-03
LIMLP_RS01295	LIMLP_01295	-1,08	-2,1	1,2,E-06
LIMLP_RS01320	LIMLP_01320	-1,01	-2,0	1,2,E-02
LIMLP_RS01340	LIMLP_01340	0,62	1,5	4,0,E-02
LIMLP_RS01345	LIMLP_01345	0,79	1,7	7,9,E-03
LIMLP_RS01355	LIMLP_01355	-0,92	-1,9	9,5,E-04
LIMLP_RS01375	LIMLP_01375	-0,96	-1,9	2,4,E-03
LIMLP_RS01380	LIMLP_01380	-0,86	-1,8	3,4,E-02
LIMLP_RS01385	LIMLP_01385	-1,44	-2,7	1,6,E-08
LIMLP_RS01395	LIMLP_01395	-1,34	-2,5	7,0,E-10
LIMLP_RS01405	LIMLP_01405	0,84	1,8	4,9,E-02
LIMLP_RS01435	LIMLP_01435	-0,97	-2,0	7,6,E-05
LIMLP_RS01440	LIMLP_01440	-0,85	-1,8	1,2,E-03
LIMLP_RS01445	LIMLP_01445	-1,37	-2,6	8,1,E-04
LIMLP_RS01450	LIMLP_01450	-0,96	-1,9	2,7,E-03
LIMLP_RS01455	LIMLP_01455	-1,38	-2,6	3,9,E-09
LIMLP_RS01460	LIMLP_01460	-1,08	-2,1	1,5,E-06
LIMLP_RS01475	LIMLP_01475	-1,68	-3,2	1,9,E-04
LIMLP_RS01480	LIMLP_01480	-1,85	-3,6	5,6,E-06
LIMLP_RS01485	LIMLP_01485	-1,00	-2,0	7,8,E-03
LIMLP_RS01500	LIMLP_01500	-0,67	-1,6	2,6,E-04
LIMLP_RS01505	LIMLP_01505	1,40	2,6	1,5,E-04
LIMLP_RS01625	LIMLP_01625	-2,00	-4,0	1,7,E-07
LIMLP_RS01630	LIMLP_01630	-1,47	-2,8	1,3,E-08
LIMLP_RS01665	LIMLP_01665	-1,00	-2,0	3,8,E-06
LIMLP_RS01670	LIMLP_01670	-0,97	-2,0	5,9,E-04
LIMLP_RS01675	LIMLP_01675	-0,80	-1,7	5,7,E-04
LIMLP_RS01690	LIMLP_01690	-2,24	-4,7	2,2,E-06
LIMLP_RS01775	LIMLP_01775	1,22	2,3	4,9,E-04
LIMLP_RS01780	LIMLP_01780	1,70	3,2	3,9,E-19
LIMLP_RS01800	LIMLP_01800	0,96	2,0	1,1,E-04
LIMLP_RS01840	LIMLP_01840	-1,31	-2,5	1,4,E-06
LIMLP_RS01845	LIMLP_01845	0,64	1,6	2,9,E-02
LIMLP_RS01850	LIMLP_01850	-0,70	-1,6	1,2,E-02
LIMLP_RS01865	LIMLP_01865	2,77	6,8	8,3,E-25
LIMLP_RS01875	LIMLP_01875	-1,37	-2,6	3,9,E-09
LIMLP_RS01880	LIMLP_01880	-0,75	-1,7	3,3,E-02
LIMLP_RS01895	LIMLP_01895	0,66	1,6	2,1,E-04
LIMLP_RS01925	LIMLP_01925	-0,54	-1,5	1,3,E-03

LIMLP_RS01930	LIMLP_01930	-0,70	-1,6	2,4,E-04
LIMLP_RS01990	LIMLP_01990	3,49	11,2	2,3,E-13
LIMLP_RS01995	LIMLP_01995	0,61	1,5	1,2,E-03
LIMLP_RS02025	LIMLP_02025	-0,59	-1,5	3,6,E-02
LIMLP_RS02030	LIMLP_02030	-0,61	-1,5	4,2,E-02
LIMLP_RS02040	LIMLP_02040	1,85	3,6	2,6,E-04
LIMLP_RS02045	LIMLP_02045	2,30	4,9	6,9,E-07
LIMLP_RS02050	LIMLP_02050	1,13	2,2	1,4,E-02
LIMLP_RS02070	LIMLP_02070	-0,82	-1,8	4,7,E-02
LIMLP_RS02080	LIMLP_02080	1,60	3,0	2,0,E-03
LIMLP_RS02090	LIMLP_02090	-1,07	-2,1	4,9,E-02
LIMLP_RS02095	LIMLP_02095	-1,28	-2,4	1,0,E-06
LIMLP_RS02105	LIMLP_02105	1,37	2,6	1,5,E-02
LIMLP_RS02110	LIMLP_02110	0,95	1,9	4,6,E-04
LIMLP_RS02120	LIMLP_02120	-1,75	-3,4	2,9,E-04
LIMLP_RS02135	LIMLP_02135	1,60	3,0	5,0,E-12
LIMLP_RS02140	LIMLP_02140	0,81	1,7	3,1,E-02
LIMLP_RS02150	LIMLP_02150	-1,00	-2,0	7,6,E-04
LIMLP_RS02180	LIMLP_02180	-1,04	-2,1	1,1,E-03
LIMLP_RS02205	LIMLP_02205	-1,18	-2,3	1,0,E-02
LIMLP_RS02215	LIMLP_02215	0,66	1,6	4,5,E-02
LIMLP_RS02225	LIMLP_02225	-2,50	-5,7	3,0,E-12
LIMLP_RS02235	LIMLP_02235	1,22	2,3	3,7,E-05
LIMLP_RS02270	LIMLP_02270	0,75	1,7	3,5,E-02
LIMLP_RS02280	LIMLP_02280	0,71	1,6	4,8,E-03
LIMLP_RS02285	LIMLP_02285	-0,57	-1,5	2,2,E-02
LIMLP_RS02295	LIMLP_02295	-1,69	-3,2	1,9,E-07
LIMLP_RS02310	LIMLP_02310	0,60	1,5	2,6,E-02
LIMLP_RS02340	LIMLP_02340	-0,90	-1,9	2,6,E-03
LIMLP_RS02355	LIMLP_02355	0,64	1,6	9,3,E-03
LIMLP_RS02360	LIMLP_02360	1,29	2,4	4,5,E-06
LIMLP_RS02365	LIMLP_02365	1,09	2,1	8,1,E-05
LIMLP_RS02375	LIMLP_02375	-0,93	-1,9	1,3,E-03
LIMLP_RS02390	LIMLP_02390	-0,80	-1,7	1,6,E-02
LIMLP_RS02400	LIMLP_02400	-2,39	-5,2	4,8,E-10
LIMLP_RS02405	LIMLP_02405	-0,69	-1,6	4,7,E-02
LIMLP_RS02455	LIMLP_02455	-0,82	-1,8	1,2,E-03
LIMLP_RS02470	LIMLP_02470	-0,93	-1,9	1,7,E-02
LIMLP_RS02480	LIMLP_02480	-0,65	-1,6	3,2,E-03
LIMLP_RS02515	LIMLP_02515	4,89	29,6	4,1,E-09
LIMLP_RS02520	LIMLP_02520	4,83	28,4	9,1,E-09
LIMLP_RS02525	LIMLP_02525	3,52	11,5	1,1,E-16
LIMLP_RS02535	LIMLP_02535	1,18	2,3	1,1,E-02
LIMLP_RS02550	LIMLP_02550	0,90	1,9	9,4,E-03
LIMLP_RS02555	LIMLP_02555	0,46	1,4	2,2,E-02
LIMLP_RS02560	LIMLP_02560	1,38	2,6	4,5,E-04

LIMLP_RS02565	LIMLP_02565	-0,83	-1,8	8,2,E-03
LIMLP_RS02585	LIMLP_02585	-0,70	-1,6	4,8,E-03
LIMLP_RS02590	LIMLP_02590	-0,72	-1,6	2,7,E-03
LIMLP_RS02595	LIMLP_02595	-0,75	-1,7	3,9,E-05
LIMLP_RS02605	LIMLP_02605	-0,99	-2,0	2,2,E-04
LIMLP_RS02615	LIMLP_02615	1,18	2,3	1,6,E-03
LIMLP_RS02640	LIMLP_02640	-0,82	-1,8	1,3,E-04
LIMLP_RS02645	LIMLP_02645	-0,39	-1,3	3,5,E-02
LIMLP_RS02680	LIMLP_02680	0,82	1,8	1,7,E-03
LIMLP_RS02700	LIMLP_02700	-1,19	-2,3	1,7,E-04
LIMLP_RS02720	LIMLP_02720	1,10	2,1	6,5,E-08
LIMLP_RS02725	LIMLP_02725	1,07	2,1	2,2,E-09
LIMLP_RS02730	LIMLP_02730	0,45	1,4	1,7,E-02
LIMLP_RS02745	LIMLP_02745	-0,50	-1,4	1,7,E-02
LIMLP_RS02750	LIMLP_02750	-1,42	-2,7	8,5,E-07
LIMLP_RS02755	LIMLP_02755	-0,76	-1,7	1,8,E-04
LIMLP_RS02815	LIMLP_02815	-0,76	-1,7	7,9,E-03
LIMLP_RS02845	LIMLP_02845	2,88	7,3	1,0,E-06
LIMLP_RS02895	LIMLP_02895	-1,09	-2,1	8,7,E-05
LIMLP_RS02945	LIMLP_02945	-0,78	-1,7	1,0,E-04
LIMLP_RS02955	LIMLP_02955	1,24	2,4	8,5,E-04
LIMLP_RS02990	LIMLP_02990	0,67	1,6	3,5,E-04
LIMLP_RS03055	LIMLP_03055	1,28	2,4	1,4,E-03
LIMLP_RS03060	LIMLP_03060	1,67	3,2	3,5,E-05
LIMLP_RS03095	LIMLP_03095	-0,91	-1,9	2,6,E-02
LIMLP_RS03100	LIMLP_03100	-0,90	-1,9	2,4,E-02
LIMLP_RS03110	LIMLP_03110	-0,90	-1,9	1,4,E-02
LIMLP_RS03115	LIMLP_03115	-0,77	-1,7	3,3,E-02
LIMLP_RS03135	LIMLP_03135	-0,85	-1,8	2,4,E-02
LIMLP_RS03140	LIMLP_03140	-0,83	-1,8	3,0,E-02
LIMLP_RS03155	LIMLP_03155	-0,84	-1,8	1,8,E-02
LIMLP_RS03175	LIMLP_03175	-0,97	-2,0	4,2,E-03
LIMLP_RS03180	LIMLP_03180	-0,83	-1,8	3,0,E-02
LIMLP_RS03205	LIMLP_03205	-0,62	-1,5	3,8,E-02
LIMLP_RS03210	LIMLP_03210	-0,92	-1,9	3,6,E-03
LIMLP_RS03215	LIMLP_03215	-0,93	-1,9	5,3,E-03
LIMLP_RS03220	LIMLP_03220	-0,84	-1,8	5,6,E-03
LIMLP_RS03270	LIMLP_03270	-0,65	-1,6	4,5,E-03
LIMLP_RS03275	LIMLP_03275	-0,66	-1,6	1,0,E-02
LIMLP_RS03285	LIMLP_03285	-0,89	-1,9	6,7,E-06
LIMLP_RS03320	LIMLP_03320	-0,61	-1,5	1,0,E-02
LIMLP_RS03340	LIMLP_03340	0,82	1,8	2,0,E-02
LIMLP_RS03355	LIMLP_03355	-0,88	-1,8	1,0,E-02
LIMLP_RS03360	LIMLP_03360	2,00	4,0	5,2,E-05
LIMLP_RS03380	LIMLP_03380	1,22	2,3	4,1,E-03
LIMLP_RS03390	LIMLP_03390	1,49	2,8	1,2,E-09

LIMLP_RS03395	LIMLP_03395	1,65	3,1	1,0,E-09
LIMLP_RS03400	LIMLP_03400	1,51	2,8	5,6,E-06
LIMLP_RS03410	LIMLP_03410	1,13	2,2	2,1,E-03
LIMLP_RS03420	LIMLP_03425	2,33	5,0	7,9,E-06
LIMLP_RS03425	LIMLP_03430	2,22	4,6	1,6,E-05
LIMLP_RS03430	LIMLP_03435	1,09	2,1	2,3,E-03
LIMLP_RS03435	LIMLP_03440	0,83	1,8	3,0,E-02
LIMLP_RS03495	LIMLP_03500	-0,44	-1,4	4,8,E-02
LIMLP_RS03535	LIMLP_03540	-0,63	-1,5	5,0,E-02
LIMLP_RS03540	LIMLP_03545	-0,92	-1,9	6,3,E-03
LIMLP_RS03580	LIMLP_03585	-1,19	-2,3	2,2,E-05
LIMLP_RS03585	LIMLP_03590	-1,19	-2,3	1,4,E-02
LIMLP_RS03595	LIMLP_03600	-0,71	-1,6	2,7,E-02
LIMLP_RS03600	LIMLP_03605	-0,81	-1,8	2,4,E-02
LIMLP_RS03605	LIMLP_03610	-0,87	-1,8	9,9,E-03
LIMLP_RS03620	LIMLP_03625	-1,19	-2,3	5,3,E-06
LIMLP_RS03625	LIMLP_03630	-2,32	-5,0	2,0,E-12
LIMLP_RS03630	LIMLP_03635	-1,29	-2,4	2,1,E-03
LIMLP_RS03635	LIMLP_03640	-1,04	-2,1	1,2,E-03
LIMLP_RS03665	LIMLP_03675	0,84	1,8	2,2,E-07
LIMLP_RS03680	LIMLP_03690	1,15	2,2	2,8,E-03
LIMLP_RS03695	LIMLP_03705	-1,09	-2,1	5,6,E-06
LIMLP_RS03700	LIMLP_03710	-1,34	-2,5	2,3,E-04
LIMLP_RS03705	LIMLP_03715	-1,11	-2,2	4,8,E-04
LIMLP_RS03710	LIMLP_03720	-1,03	-2,0	4,5,E-04
LIMLP_RS03715	LIMLP_03725	-0,88	-1,8	4,7,E-03
LIMLP_RS03720	LIMLP_03730	-0,68	-1,6	2,0,E-02
LIMLP_RS03750	LIMLP_03760	-0,51	-1,4	2,6,E-02
LIMLP_RS03770	LIMLP_03780	-0,64	-1,6	1,8,E-02
LIMLP_RS03795	LIMLP_03805	2,39	5,2	1,1,E-17
LIMLP_RS03800	LIMLP_03810	1,09	2,1	1,2,E-03
LIMLP_RS03810	LIMLP_03820	-0,57	-1,5	5,0,E-02
LIMLP_RS03830	LIMLP_03840	0,95	1,9	1,9,E-04
LIMLP_RS03835	LIMLP_03845	-0,57	-1,5	3,8,E-04
LIMLP_RS03840	LIMLP_03850	-0,98	-2,0	8,8,E-13
LIMLP_RS03845	LIMLP_03855	-0,93	-1,9	1,8,E-03
LIMLP_RS03865	LIMLP_03875	-1,24	-2,4	1,4,E-08
LIMLP_RS03875	LIMLP_03885	-0,60	-1,5	9,8,E-03
LIMLP_RS03970	LIMLP_03980	0,58	1,5	3,1,E-02
LIMLP_RS03975	LIMLP_03985	-0,46	-1,4	2,6,E-03
LIMLP_RS03985	LIMLP_03995	-0,30	-1,2	2,0,E-02
LIMLP_RS04005	LIMLP_04015	1,65	3,1	8,0,E-16
LIMLP_RS04010	LIMLP_04020	-0,95	-1,9	3,6,E-02
LIMLP_RS04040	LIMLP_04050	-1,25	-2,4	2,3,E-05
LIMLP_RS04045	LIMLP_04055	-1,84	-3,6	1,9,E-06
LIMLP_RS04050	LIMLP_04060	-1,78	-3,4	4,0,E-10

LIMLP_RS04085	LIMLP_04095	1,90	3,7	5,4,E-08
LIMLP_RS23255	LIMLP_04100	2,40	5,3	5,9,E-17
LIMLP_RS04095	LIMLP_04105	2,55	5,9	3,4,E-10
LIMLP_RS04100	LIMLP_04110	3,34	10,1	6,8,E-09
LIMLP_RS04120	LIMLP_04130	-0,69	-1,6	7,2,E-03
LIMLP_RS04135	LIMLP_04145	-0,78	-1,7	2,4,E-03
LIMLP_RS04160	LIMLP_04170	-0,82	-1,8	6,6,E-03
LIMLP_RS04165	LIMLP_04175	-0,94	-1,9	1,8,E-02
LIMLP_RS04190	LIMLP_04200	-0,80	-1,7	3,8,E-03
LIMLP_RS04195	LIMLP_04205	-1,09	-2,1	7,3,E-04
LIMLP_RS04200	LIMLP_04210	-0,91	-1,9	3,9,E-04
LIMLP_RS04205	LIMLP_04215	-1,01	-2,0	7,1,E-04
LIMLP_RS04210	LIMLP_04220	-1,13	-2,2	4,4,E-08
LIMLP_RS04215	LIMLP_04225	-1,12	-2,2	1,3,E-04
LIMLP_RS22510	LIMLP_04265	-2,95	-7,7	3,6,E-02
LIMLP_RS04260	LIMLP_04270	-2,34	-5,1	2,2,E-02
LIMLP_RS04290	LIMLP_04300	0,63	1,6	2,6,E-03
LIMLP_RS04300	LIMLP_04310	3,05	8,3	1,4,E-26
LIMLP_RS22520	LIMLP_04355	-0,72	-1,6	4,4,E-02
LIMLP_RS04365	LIMLP_04375	1,34	2,5	3,9,E-03
LIMLP_RS04370	LIMLP_04380	-1,65	-3,1	1,3,E-10
LIMLP_RS04375	LIMLP_04385	-1,13	-2,2	5,2,E-06
LIMLP_RS04400	LIMLP_04410	1,32	2,5	2,5,E-04
LIMLP_RS04430	LIMLP_04440	-0,58	-1,5	1,8,E-02
LIMLP_RS04470	LIMLP_04480	3,06	8,3	1,6,E-09
LIMLP_RS04485	LIMLP_04495	-0,89	-1,9	3,5,E-06
LIMLP_RS04510	LIMLP_04520	1,48	2,8	2,9,E-04
LIMLP_RS04520	LIMLP_04530	0,49	1,4	9,8,E-03
LIMLP_RS04540	LIMLP_04550	1,21	2,3	8,4,E-09
LIMLP_RS04545	LIMLP_04555	2,79	6,9	3,0,E-26
LIMLP_RS04550	LIMLP_04560	0,80	1,7	3,9,E-02
LIMLP_RS04570	LIMLP_04580	-2,04	-4,1	2,2,E-06
LIMLP_RS04580	LIMLP_04590	-1,27	-2,4	2,0,E-15
LIMLP_RS04585	LIMLP_04595	-1,21	-2,3	3,2,E-02
LIMLP_RS04645	LIMLP_04655	1,64	3,1	3,1,E-05
LIMLP_RS04655	LIMLP_04665	-0,77	-1,7	1,1,E-02
LIMLP_RS04665	LIMLP_04675	0,81	1,8	1,1,E-02
LIMLP_RS04705	LIMLP_04715	0,93	1,9	1,6,E-02
LIMLP_RS04710	LIMLP_04720	1,02	2,0	2,5,E-05
LIMLP_RS04715	LIMLP_04725	1,56	2,9	5,1,E-03
LIMLP_RS04720	LIMLP_04730	1,34	2,5	7,2,E-03
LIMLP_RS04725	LIMLP_04735	1,04	2,1	1,9,E-03
LIMLP_RS04780	LIMLP_04790	1,11	2,2	2,5,E-05
LIMLP_RS04800	LIMLP_04810	-0,84	-1,8	1,1,E-04
LIMLP_RS04830	LIMLP_04840	0,99	2,0	1,2,E-02
LIMLP_RS04835	LIMLP_04845	-0,97	-2,0	5,5,E-03

LIMLP_RS04930	LIMLP_04940	-0,84	-1,8	3,0,E-02
LIMLP_RS04950	LIMLP_04960	-0,71	-1,6	3,1,E-02
LIMLP_RS05020	LIMLP_05030	1,58	3,0	2,0,E-10
LIMLP_RS05025	LIMLP_05035	1,01	2,0	7,7,E-03
LIMLP_RS05030	LIMLP_05040	0,82	1,8	2,5,E-02
LIMLP_RS05035	LIMLP_05045	1,00	2,0	3,6,E-02
LIMLP_RS23265	LIMLP_05050	0,65	1,6	4,9,E-02
LIMLP_RS05045	LIMLP_05055	0,95	1,9	1,9,E-02
LIMLP_RS05050	LIMLP_05060	-1,27	-2,4	5,8,E-05
LIMLP_RS05105	LIMLP_05110	1,44	2,7	4,5,E-03
LIMLP_RS05110	LIMLP_05115	2,03	4,1	2,2,E-02
LIMLP_RS23455	LIMLP_05120	2,18	4,5	2,0,E-02
LIMLP_RS05125	LIMLP_05130	-1,07	-2,1	4,4,E-05
LIMLP_RS05130	LIMLP_05135	2,62	6,2	6,0,E-13
LIMLP_RS05135	LIMLP_05140	-1,39	-2,6	2,8,E-03
LIMLP_RS05150	LIMLP_05155	0,43	1,3	4,8,E-02
LIMLP_RS05175	LIMLP_05180	-0,68	-1,6	3,3,E-02
LIMLP_RS05190	LIMLP_05195	1,03	2,0	2,3,E-09
LIMLP_RS05210	LIMLP_05215	2,32	5,0	2,3,E-05
LIMLP_RS05230	LIMLP_05235	-1,28	-2,4	3,0,E-12
LIMLP_RS05235	LIMLP_05240	1,39	2,6	3,9,E-02
LIMLP_RS05265	LIMLP_05270	0,61	1,5	3,5,E-02
LIMLP_RS05270	LIMLP_05275	0,58	1,5	5,0,E-02
LIMLP_RS05285	LIMLP_05290	0,97	2,0	2,0,E-02
LIMLP_RS05290	LIMLP_05295	-1,18	-2,3	1,8,E-03
LIMLP_RS05295	LIMLP_05300	-0,89	-1,9	2,0,E-02
LIMLP_RS05325	LIMLP_05330	-1,12	-2,2	3,5,E-02
LIMLP_RS05330	LIMLP_05335	0,62	1,5	3,4,E-02
LIMLP_RS05335	LIMLP_05340	-0,61	-1,5	2,8,E-02
LIMLP_RS05365	LIMLP_05370	-1,06	-2,1	6,2,E-05
LIMLP_RS05370	LIMLP_05375	-1,01	-2,0	7,4,E-05
LIMLP_RS05375	LIMLP_05380	-1,59	-3,0	3,8,E-11
LIMLP_RS05380	LIMLP_05385	1,24	2,4	2,2,E-04
LIMLP_RS05400	LIMLP_05405	1,39	2,6	2,8,E-04
LIMLP_RS05405	LIMLP_05410	-0,49	-1,4	3,0,E-02
LIMLP_RS05435	LIMLP_05440	0,94	1,9	1,3,E-05
LIMLP_RS05445	LIMLP_05450	-1,67	-3,2	4,2,E-07
LIMLP_RS05450	LIMLP_05455	-1,43	-2,7	1,7,E-06
LIMLP_RS05455	LIMLP_05460	-0,98	-2,0	9,7,E-04
LIMLP_RS05460	LIMLP_05465	-0,76	-1,7	7,2,E-06
LIMLP_RS05465	LIMLP_05470	-0,67	-1,6	4,2,E-03
LIMLP_RS05500	LIMLP_05505	-0,44	-1,4	7,6,E-03
LIMLP_RS05505	LIMLP_05510	-0,56	-1,5	9,8,E-04
LIMLP_RS05515	LIMLP_05520	-1,37	-2,6	8,7,E-04
LIMLP_RS05530	LIMLP_05535	0,82	1,8	2,0,E-03
LIMLP_RS05545	LIMLP_05550	-1,14	-2,2	1,2,E-03

LIMLP_RS05555	LIMLP_05560	0,92	1,9	3,3,E-02
LIMLP_RS05575	LIMLP_05580	1,03	2,0	4,9,E-04
LIMLP_RS05580	LIMLP_05585	1,17	2,2	3,4,E-05
LIMLP_RS05600	LIMLP_05605	-0,85	-1,8	4,4,E-02
LIMLP_RS05605	LIMLP_05610	-0,94	-1,9	4,3,E-02
LIMLP_RS05615	LIMLP_05620	2,00	4,0	7,8,E-05
LIMLP_RS05625	LIMLP_05630	-0,56	-1,5	4,2,E-02
LIMLP_RS05640	LIMLP_05645	-1,73	-3,3	7,0,E-08
LIMLP_RS05645	LIMLP_05650	-1,31	-2,5	2,5,E-15
LIMLP_RS05650	LIMLP_05655	-1,08	-2,1	1,9,E-06
LIMLP_RS05655	LIMLP_05660	-0,68	-1,6	4,5,E-04
LIMLP_RS05685	LIMLP_05690	-0,74	-1,7	9,3,E-03
LIMLP_RS05690	LIMLP_05695	1,87	3,7	2,9,E-04
LIMLP_RS05705	LIMLP_05710	1,08	2,1	3,8,E-02
LIMLP_RS05715	LIMLP_05720	-0,54	-1,5	9,9,E-03
LIMLP_RS05720	LIMLP_05725	-1,51	-2,9	2,2,E-06
LIMLP_RS05745	LIMLP_05750	-1,17	-2,2	3,9,E-09
LIMLP_RS05750	LIMLP_05755	-1,28	-2,4	8,0,E-06
LIMLP_RS05770	LIMLP_05775	0,49	1,4	1,5,E-02
LIMLP_RS05805	LIMLP_05810	-0,51	-1,4	1,4,E-02
LIMLP_RS05840	LIMLP_05845	-0,67	-1,6	3,7,E-02
LIMLP_RS05870	LIMLP_05875	0,75	1,7	2,3,E-05
LIMLP_RS05900	LIMLP_05910	-1,43	-2,7	5,6,E-11
LIMLP_RS05905	LIMLP_05915	-1,81	-3,5	5,8,E-09
LIMLP_RS05910	LIMLP_05920	-1,74	-3,3	1,1,E-07
LIMLP_RS05950	LIMLP_05960	0,80	1,7	4,9,E-04
LIMLP_RS05955	LIMLP_05965	0,96	1,9	1,5,E-03
LIMLP_RS05975	LIMLP_05985	-1,08	-2,1	4,0,E-02
LIMLP_RS05995	LIMLP_06005	1,89	3,7	4,4,E-13
LIMLP_RS06005	LIMLP_06015	-0,75	-1,7	6,7,E-03
LIMLP_RS06035	LIMLP_06045	-1,15	-2,2	1,0,E-03
LIMLP_RS06040	LIMLP_06050	-1,23	-2,3	3,0,E-03
LIMLP_RS06045	LIMLP_06055	-1,01	-2,0	1,7,E-02
LIMLP_RS06050	LIMLP_06060	-0,99	-2,0	2,7,E-02
LIMLP_RS06055	LIMLP_06065	-0,97	-2,0	3,3,E-02
LIMLP_RS06060	LIMLP_06070	-0,84	-1,8	1,8,E-02
LIMLP_RS06065	LIMLP_06075	-1,19	-2,3	4,7,E-03
LIMLP_RS06070	LIMLP_06080	-1,18	-2,3	1,8,E-03
LIMLP_RS06075	LIMLP_06085	-1,63	-3,1	2,2,E-14
LIMLP_RS06080	LIMLP_06090	-0,80	-1,7	3,1,E-02
LIMLP_RS06085	LIMLP_06095	-0,84	-1,8	2,2,E-03
LIMLP_RS06145	LIMLP_06155	-0,72	-1,6	2,4,E-02
LIMLP_RS06150	LIMLP_06160	-0,83	-1,8	1,3,E-02
LIMLP_RS06155	LIMLP_06165	-0,93	-1,9	2,4,E-02
LIMLP_RS06160	LIMLP_06170	-0,68	-1,6	3,5,E-02
LIMLP_RS06165	LIMLP_06175	-0,93	-1,9	2,5,E-03

LIMLP_RS06170	LIMLP_06180	1,93	3,8	4,5,E-07
LIMLP_RS06180	LIMLP_06190	1,08	2,1	1,7,E-02
LIMLP_RS06185	LIMLP_06195	0,72	1,6	4,3,E-03
LIMLP_RS06195	LIMLP_06205	0,67	1,6	4,3,E-02
LIMLP_RS06280	LIMLP_06290	1,53	2,9	2,1,E-03
LIMLP_RS06295	LIMLP_06305	-0,95	-1,9	2,4,E-02
LIMLP_RS06300	LIMLP_06310	-0,62	-1,5	2,4,E-02
LIMLP_RS06305	LIMLP_06315	-0,55	-1,5	1,8,E-02
LIMLP_RS06340	LIMLP_06350	-1,09	-2,1	2,9,E-02
LIMLP_RS06365	LIMLP_06375	1,01	2,0	2,5,E-05
LIMLP_RS06375	LIMLP_06385	-0,74	-1,7	1,8,E-02
LIMLP_RS06390	LIMLP_06400	0,66	1,6	1,7,E-02
LIMLP_RS06395	LIMLP_06405	0,96	2,0	1,6,E-04
LIMLP_RS06410	LIMLP_06420	0,87	1,8	4,5,E-02
LIMLP_RS06415	LIMLP_06425	1,31	2,5	1,2,E-02
LIMLP_RS06420	LIMLP_06430	1,42	2,7	3,3,E-02
LIMLP_RS06455	LIMLP_06465	0,81	1,8	4,0,E-03
LIMLP_RS06470	LIMLP_06480	-1,71	-3,3	2,0,E-12
LIMLP_RS06475	LIMLP_06485	-0,82	-1,8	8,2,E-05
LIMLP_RS06480	LIMLP_06490	-0,56	-1,5	8,2,E-04
LIMLP_RS06485	LIMLP_06495	-0,57	-1,5	4,1,E-04
LIMLP_RS06490	LIMLP_06500	-0,68	-1,6	3,8,E-04
LIMLP_RS06495	LIMLP_06505	-0,86	-1,8	1,9,E-02
LIMLP_RS06540	LIMLP_06550	0,50	1,4	3,2,E-03
LIMLP_RS06560	LIMLP_06570	0,93	1,9	5,8,E-03
LIMLP_RS06565	LIMLP_06575	1,15	2,2	1,4,E-03
LIMLP_RS06590	LIMLP_06600	-1,28	-2,4	2,1,E-03
LIMLP_RS06615	LIMLP_06625	-0,69	-1,6	5,5,E-03
LIMLP_RS06620	LIMLP_06630	1,11	2,2	4,3,E-03
LIMLP_RS06635	LIMLP_06645	0,55	1,5	1,9,E-02
LIMLP_RS06640	LIMLP_06650	-0,84	-1,8	2,6,E-03
LIMLP_RS06645	LIMLP_06655	-1,06	-2,1	4,3,E-03
LIMLP_RS06665	LIMLP_06675	-1,00	-2,0	1,2,E-03
LIMLP_RS06695	LIMLP_06705	-0,70	-1,6	3,4,E-02
LIMLP_RS06700	LIMLP_06710	-0,65	-1,6	5,6,E-03
LIMLP_RS06705	LIMLP_06715	-0,82	-1,8	5,3,E-05
LIMLP_RS06710	LIMLP_06720	-0,76	-1,7	1,0,E-05
LIMLP_RS06715	LIMLP_06725	-0,60	-1,5	2,7,E-04
LIMLP_RS06740	LIMLP_06750	-0,83	-1,8	2,3,E-03
LIMLP_RS06745	LIMLP_06755	-0,78	-1,7	5,5,E-04
LIMLP_RS06760	LIMLP_06770	0,72	1,6	2,1,E-02
LIMLP_RS06775	LIMLP_06785	1,27	2,4	8,0,E-03
LIMLP_RS06780	LIMLP_06790	1,20	2,3	2,3,E-03
LIMLP_RS06815	LIMLP_06825	-0,61	-1,5	1,3,E-02
LIMLP_RS06830	LIMLP_06840	0,82	1,8	9,7,E-04
LIMLP_RS06840	LIMLP_06850	0,85	1,8	7,7,E-04

LIMLP_RS06850	LIMLP_06860	1,17	2,3	1,1,E-03
LIMLP_RS06855	LIMLP_06865	3,48	11,1	8,4,E-06
LIMLP_RS06860	LIMLP_06870	0,88	1,8	2,0,E-02
LIMLP_RS06905	LIMLP_06915	1,11	2,2	2,9,E-04
LIMLP_RS06910	LIMLP_06920	0,80	1,7	2,4,E-09
LIMLP_RS06975	LIMLP_06985	-0,48	-1,4	3,7,E-02
LIMLP_RS06995	LIMLP_07005	2,21	4,6	8,1,E-19
LIMLP_RS07000	LIMLP_07010	2,02	4,1	1,6,E-15
LIMLP_RS07015	LIMLP_07025	-1,11	-2,2	2,2,E-03
LIMLP_RS07020	LIMLP_07030	-1,56	-2,9	8,9,E-06
LIMLP_RS07025	LIMLP_07035	2,53	5,8	1,7,E-07
LIMLP_RS07030	LIMLP_07040	3,08	8,4	2,7,E-07
LIMLP_RS07035	LIMLP_07045	2,46	5,5	1,3,E-14
LIMLP_RS07045	LIMLP_07055	1,68	3,2	6,1,E-11
LIMLP_RS07060	LIMLP_07070	1,31	2,5	1,1,E-02
LIMLP_RS07090	LIMLP_07100	-0,58	-1,5	3,3,E-02
LIMLP_RS07100	LIMLP_07110	-1,12	-2,2	2,1,E-04
LIMLP_RS07110	LIMLP_07120	-0,86	-1,8	6,5,E-03
LIMLP_RS07115	LIMLP_07125	-0,77	-1,7	2,4,E-02
LIMLP_RS07120	LIMLP_07130	2,24	4,7	8,7,E-05
LIMLP_RS07130	LIMLP_07145	1,41	2,7	8,1,E-04
LIMLP_RS07135	LIMLP_07150	1,90	3,7	1,1,E-02
LIMLP_RS07140	LIMLP_07155	-0,56	-1,5	1,2,E-02
LIMLP_RS07145	LIMLP_07160	-0,81	-1,8	7,6,E-06
LIMLP_RS07150	LIMLP_07165	1,01	2,0	4,5,E-03
LIMLP_RS07165	LIMLP_07180	-0,81	-1,8	3,5,E-02
LIMLP_RS07240	LIMLP_07255	-1,11	-2,2	1,7,E-08
LIMLP_RS07280	LIMLP_07295	-0,42	-1,3	4,1,E-03
LIMLP_RS07285	LIMLP_07300	-0,64	-1,6	1,5,E-02
LIMLP_RS07290	LIMLP_07305	-0,75	-1,7	4,2,E-03
LIMLP_RS07295	LIMLP_07310	-1,05	-2,1	5,1,E-06
LIMLP_RS07310	LIMLP_07325	1,12	2,2	4,1,E-04
LIMLP_RS07350	LIMLP_07365	0,58	1,5	2,9,E-02
LIMLP_RS07355	LIMLP_07370	0,90	1,9	5,9,E-04
LIMLP_RS07360	LIMLP_07375	-0,32	-1,3	4,7,E-02
LIMLP_RS07390	LIMLP_07405	-0,89	-1,8	3,1,E-02
LIMLP_RS07395	LIMLP_07410	-0,98	-2,0	6,1,E-03
LIMLP_RS07410	LIMLP_07425	-1,08	-2,1	4,9,E-02
LIMLP_RS07425	LIMLP_07440	-1,25	-2,4	2,8,E-02
LIMLP_RS07430	LIMLP_07445	-1,44	-2,7	8,6,E-03
LIMLP_RS07435	LIMLP_07450	-1,65	-3,1	4,6,E-03
LIMLP_RS07455	LIMLP_07470	1,21	2,3	8,7,E-03
LIMLP_RS07460	LIMLP_07475	-1,22	-2,3	1,9,E-06
LIMLP_RS07465	LIMLP_07480	1,07	2,1	4,3,E-02
LIMLP_RS07485	LIMLP_07500	1,48	2,8	1,7,E-07
LIMLP_RS07500	LIMLP_07515	0,57	1,5	9,8,E-04

LIMLP_RS07505	LIMLP_07520	0,34	1,3	3,1,E-02
LIMLP_RS07510	LIMLP_07525	0,62	1,5	4,1,E-03
LIMLP_RS07515	LIMLP_07530	0,77	1,7	3,3,E-03
LIMLP_RS07520	LIMLP_07535	0,71	1,6	4,3,E-03
LIMLP_RS07525	LIMLP_07540	1,12	2,2	3,0,E-05
LIMLP_RS07530	LIMLP_07545	0,84	1,8	1,8,E-05
LIMLP_RS07535	LIMLP_07550	0,65	1,6	3,2,E-02
LIMLP_RS07540	LIMLP_07555	0,43	1,3	1,3,E-02
LIMLP_RS07545	LIMLP_07560	0,53	1,4	3,1,E-02
LIMLP_RS07555	LIMLP_07570	0,67	1,6	1,2,E-02
LIMLP_RS07605	LIMLP_07620	1,69	3,2	2,9,E-05
LIMLP_RS07610	LIMLP_07625	1,43	2,7	4,5,E-05
LIMLP_RS07615	LIMLP_07630	1,40	2,6	6,9,E-05
LIMLP_RS07625	LIMLP_07640	-1,85	-3,6	2,2,E-08
LIMLP_RS07630	LIMLP_07645	0,88	1,8	1,7,E-02
LIMLP_RS07640	LIMLP_07655	-0,78	-1,7	9,4,E-03
LIMLP_RS07715	LIMLP_07730	0,77	1,7	2,7,E-05
LIMLP_RS07735	LIMLP_07750	1,46	2,8	6,8,E-03
LIMLP_RS07740	LIMLP_07755	0,80	1,7	3,5,E-05
LIMLP_RS07750	LIMLP_07765	0,58	1,5	7,7,E-03
LIMLP_RS07755	LIMLP_07770	0,53	1,4	1,2,E-02
LIMLP_RS07760	LIMLP_07775	0,91	1,9	1,5,E-03
LIMLP_RS07770	LIMLP_07785	1,22	2,3	3,4,E-05
LIMLP_RS07775	LIMLP_07790	0,83	1,8	4,1,E-04
LIMLP_RS07795	LIMLP_07810	0,73	1,7	6,5,E-04
LIMLP_RS07830	LIMLP_07845	2,03	4,1	1,2,E-09
LIMLP_RS07835	LIMLP_07850	1,18	2,3	1,9,E-03
LIMLP_RS07840	LIMLP_07855	-0,42	-1,3	4,8,E-02
LIMLP_RS07850	LIMLP_07865	0,50	1,4	1,2,E-02
LIMLP_RS07855	LIMLP_07870	1,35	2,5	7,4,E-03
LIMLP_RS07900	LIMLP_07915	1,20	2,3	4,5,E-02
LIMLP_RS07920	LIMLP_07935	0,64	1,6	1,2,E-03
LIMLP_RS07925	LIMLP_07940	-1,03	-2,0	5,1,E-05
LIMLP_RS07930	LIMLP_07945	-0,99	-2,0	7,1,E-05
LIMLP_RS07935	LIMLP_07950	-1,14	-2,2	1,9,E-05
LIMLP_RS07940	LIMLP_07955	0,58	1,5	3,8,E-02
LIMLP_RS07955	LIMLP_07970	-2,17	-4,5	3,1,E-18
LIMLP_RS07975	LIMLP_07990	-0,94	-1,9	5,9,E-03
LIMLP_RS07980	LIMLP_07995	-1,02	-2,0	3,0,E-06
LIMLP_RS07985	LIMLP_08000	-0,90	-1,9	6,0,E-05
LIMLP_RS08035	LIMLP_08050	-0,80	-1,7	1,9,E-02
LIMLP_RS08045	LIMLP_08060	-0,89	-1,9	5,0,E-03
LIMLP_RS08050	LIMLP_08065	-1,50	-2,8	2,8,E-14
LIMLP_RS08100	LIMLP_08115	1,06	2,1	4,8,E-04
LIMLP_RS08105	LIMLP_08120	0,83	1,8	6,2,E-03
LIMLP_RS08110	LIMLP_08125	1,16	2,2	5,3,E-04

LIMLP_RS08115	LIMLP_08130	0,76	1,7	7,5,E-03
LIMLP_RS08120	LIMLP_08135	0,52	1,4	3,0,E-02
LIMLP_RS08135	LIMLP_08150	-0,93	-1,9	5,4,E-03
LIMLP_RS08180	LIMLP_08195	-0,69	-1,6	2,8,E-02
LIMLP_RS08200	LIMLP_08215	1,32	2,5	2,2,E-02
LIMLP_RS08260	LIMLP_08275	1,91	3,8	6,0,E-05
LIMLP_RS08265	LIMLP_08280	1,19	2,3	4,7,E-02
LIMLP_RS08275	LIMLP_08290	1,58	3,0	5,6,E-03
LIMLP_RS08280	LIMLP_08295	1,99	4,0	9,1,E-05
LIMLP_RS08285	LIMLP_08300	1,04	2,1	5,3,E-03
LIMLP_RS08295	LIMLP_08310	1,42	2,7	5,9,E-03
LIMLP_RS08300	LIMLP_08315	2,68	6,4	2,0,E-05
LIMLP_RS08305	LIMLP_08320	2,58	6,0	1,2,E-04
LIMLP_RS08310	LIMLP_08325	1,60	3,0	6,6,E-03
LIMLP_RS08320	LIMLP_08335	1,49	2,8	2,0,E-02
LIMLP_RS08370	LIMLP_08385	1,30	2,5	1,6,E-03
LIMLP_RS08385	LIMLP_08400	1,33	2,5	1,4,E-04
LIMLP_RS08390	LIMLP_08405	-0,67	-1,6	6,0,E-03
LIMLP_RS08450	LIMLP_08465	-1,23	-2,3	1,2,E-05
LIMLP_RS08460	LIMLP_08475	-0,93	-1,9	1,9,E-05
LIMLP_RS08475	LIMLP_08490	1,42	2,7	3,4,E-02
LIMLP_RS08490	LIMLP_08505	0,50	1,4	4,1,E-03
LIMLP_RS08495	LIMLP_08510	0,91	1,9	9,7,E-03
LIMLP_RS08500	LIMLP_08515	0,54	1,5	1,3,E-02
LIMLP_RS08505	LIMLP_08520	0,53	1,4	2,6,E-02
LIMLP_RS08520	LIMLP_08535	-0,95	-1,9	2,2,E-02
LIMLP_RS08525	LIMLP_08540	-1,40	-2,6	8,5,E-09
LIMLP_RS08530	LIMLP_08545	-0,73	-1,7	3,3,E-03
LIMLP_RS08545	LIMLP_08560	0,89	1,9	7,6,E-06
LIMLP_RS08555	LIMLP_08570	1,12	2,2	5,7,E-04
LIMLP_RS08565	LIMLP_08580	1,21	2,3	1,3,E-03
LIMLP_RS08570	LIMLP_08585	1,37	2,6	1,2,E-02
LIMLP_RS08615	LIMLP_08630	0,58	1,5	3,6,E-02
LIMLP_RS08635	LIMLP_08650	-0,94	-1,9	3,2,E-04
LIMLP_RS08640	LIMLP_08655	-1,32	-2,5	4,8,E-06
LIMLP_RS08650	LIMLP_08665	0,88	1,8	2,4,E-02
LIMLP_RS08655	LIMLP_08670	0,68	1,6	8,7,E-04
LIMLP_RS08710	LIMLP_08725	0,90	1,9	1,0,E-04
LIMLP_RS08715	LIMLP_08730	0,54	1,5	1,5,E-02
LIMLP_RS08735	LIMLP_08750	0,54	1,5	1,4,E-02
LIMLP_RS08740	LIMLP_08755	-1,11	-2,2	9,5,E-07
LIMLP_RS22685	LIMLP_08760	-1,17	-2,3	1,7,E-08
LIMLP_RS08755	LIMLP_08770	-0,55	-1,5	4,9,E-02
LIMLP_RS08760	LIMLP_08775	-0,47	-1,4	1,7,E-02
LIMLP_RS08785	LIMLP_08800	0,74	1,7	7,0,E-04
LIMLP_RS08850	LIMLP_08865	-0,77	-1,7	1,0,E-02

LIMLP_RS08855	LIMLP_08870	1,04	2,1	5,4,E-03
LIMLP_RS08860	LIMLP_08875	0,85	1,8	2,8,E-03
LIMLP_RS08865	LIMLP_08880	0,96	1,9	3,7,E-03
LIMLP_RS08870	LIMLP_08885	1,17	2,2	1,6,E-03
LIMLP_RS08875	LIMLP_08890	1,90	3,7	5,6,E-14
LIMLP_RS08905	LIMLP_08920	0,69	1,6	8,6,E-05
LIMLP_RS08910	LIMLP_08925	0,87	1,8	3,8,E-02
LIMLP_RS08915	LIMLP_08930	-0,72	-1,6	1,6,E-05
LIMLP_RS08925	LIMLP_08940	0,89	1,9	6,4,E-03
LIMLP_RS08930	LIMLP_08945	1,40	2,6	1,2,E-04
LIMLP_RS08935	LIMLP_08950	0,86	1,8	6,3,E-03
LIMLP_RS08960	LIMLP_08975	1,63	3,1	2,6,E-02
LIMLP_RS08965	LIMLP_08980	1,34	2,5	1,2,E-03
LIMLP_RS08970	LIMLP_08985	1,36	2,6	8,2,E-08
LIMLP_RS08975	LIMLP_08990	1,36	2,6	2,6,E-13
LIMLP_RS08980	LIMLP_08995	1,39	2,6	3,4,E-14
LIMLP_RS08985	LIMLP_09000	0,64	1,6	1,5,E-02
LIMLP_RS08990	LIMLP_09005	1,49	2,8	1,2,E-06
LIMLP_RS08995	LIMLP_09010	1,85	3,6	1,3,E-06
LIMLP_RS09000	LIMLP_09015	1,92	3,8	9,1,E-09
LIMLP_RS09045	LIMLP_09060	0,46	1,4	6,3,E-03
LIMLP_RS09050	LIMLP_09065	0,65	1,6	4,3,E-03
LIMLP_RS09055	LIMLP_09070	0,97	2,0	2,0,E-04
LIMLP_RS09060	LIMLP_09075	1,01	2,0	9,5,E-06
LIMLP_RS09080	LIMLP_09095	1,64	3,1	1,7,E-03
LIMLP_RS09095	LIMLP_09110	0,73	1,7	3,4,E-02
LIMLP_RS09105	LIMLP_09120	0,87	1,8	6,2,E-03
LIMLP_RS09120	LIMLP_09135	0,57	1,5	3,6,E-02
LIMLP_RS09160	LIMLP_09175	0,77	1,7	3,7,E-03
LIMLP_RS09165	LIMLP_09180	-1,00	-2,0	2,0,E-05
LIMLP_RS09180	LIMLP_09195	1,12	2,2	6,5,E-03
LIMLP_RS09250	LIMLP_09265	-1,16	-2,2	1,5,E-04
LIMLP_RS09255	LIMLP_09270	-1,02	-2,0	1,8,E-05
LIMLP_RS09290	LIMLP_09305	0,66	1,6	4,5,E-03
LIMLP_RS09305	LIMLP_09320	0,76	1,7	2,7,E-02
LIMLP_RS09310	LIMLP_09325	0,65	1,6	6,8,E-04
LIMLP_RS09315	LIMLP_09330	0,88	1,8	4,1,E-02
LIMLP_RS09325	LIMLP_09340	-0,73	-1,7	5,8,E-03
LIMLP_RS09335	LIMLP_09350	-0,54	-1,5	1,3,E-03
LIMLP_RS09345	LIMLP_09360	-0,90	-1,9	2,3,E-04
LIMLP_RS09355	LIMLP_09370	-1,22	-2,3	3,4,E-06
LIMLP_RS09365	LIMLP_09380	1,83	3,5	6,8,E-03
LIMLP_RS09370	LIMLP_09385	1,63	3,1	1,4,E-02
LIMLP_RS09390	LIMLP_09405	-1,77	-3,4	4,0,E-06
LIMLP_RS09395	LIMLP_09410	-1,63	-3,1	1,6,E-05
LIMLP_RS09465	LIMLP_09480	-1,37	-2,6	3,4,E-02

LIMLP_RS09520	LIMLP_09535	-0,67	-1,6	5,9,E-03
LIMLP_RS09545	LIMLP_09560	0,67	1,6	5,9,E-03
LIMLP_RS09550	LIMLP_09565	0,85	1,8	3,3,E-10
LIMLP_RS09555	LIMLP_09570	0,74	1,7	6,1,E-05
LIMLP_RS09565	LIMLP_09580	1,37	2,6	1,1,E-04
LIMLP_RS09590	LIMLP_09605	-0,94	-1,9	5,9,E-03
LIMLP_RS09595	LIMLP_09610	-0,98	-2,0	1,8,E-03
LIMLP_RS09600	LIMLP_09615	-1,13	-2,2	4,1,E-09
LIMLP_RS22695	LIMLP_09620	-0,66	-1,6	2,1,E-04
LIMLP_RS09625	LIMLP_09640	0,79	1,7	2,9,E-02
LIMLP_RS09630	LIMLP_09645	1,24	2,4	8,7,E-05
LIMLP_RS09740	LIMLP_09755	0,84	1,8	2,1,E-03
LIMLP_RS09745	LIMLP_09760	0,55	1,5	5,8,E-03
LIMLP_RS09760	LIMLP_09775	1,29	2,4	3,9,E-04
LIMLP_RS09795	LIMLP_09810	-0,76	-1,7	3,6,E-02
LIMLP_RS09810	LIMLP_09825	0,61	1,5	3,9,E-06
LIMLP_RS09830	LIMLP_09845	0,74	1,7	1,9,E-04
LIMLP_RS09835	LIMLP_09850	-0,52	-1,4	4,1,E-02
LIMLP_RS09845	LIMLP_09860	0,48	1,4	1,7,E-02
LIMLP_RS09850	LIMLP_09865	0,82	1,8	1,7,E-04
LIMLP_RS09855	LIMLP_09870	1,34	2,5	5,5,E-08
LIMLP_RS09860	LIMLP_09875	0,94	1,9	1,5,E-02
LIMLP_RS09890	LIMLP_09905	1,18	2,3	1,9,E-02
LIMLP_RS09905	LIMLP_09920	-1,21	-2,3	4,1,E-09
LIMLP_RS09910	LIMLP_09925	-0,83	-1,8	1,7,E-04
LIMLP_RS09955	LIMLP_09970	0,46	1,4	1,9,E-02
LIMLP_RS09980	LIMLP_09995	1,98	3,9	1,7,E-05
LIMLP_RS09990	LIMLP_10005	1,23	2,3	3,1,E-02
LIMLP_RS09995	LIMLP_10010	1,62	3,1	2,5,E-03
LIMLP_RS10040	LIMLP_10055	-1,06	-2,1	4,5,E-02
LIMLP_RS10045	LIMLP_10060	4,42	21,4	1,3,E-22
LIMLP_RS10065	LIMLP_10080	-1,47	-2,8	8,6,E-11
LIMLP_RS10070	LIMLP_10085	1,08	2,1	2,9,E-02
LIMLP_RS10090	LIMLP_10105	-2,14	-4,4	4,0,E-12
LIMLP_RS10095	LIMLP_10110	-0,48	-1,4	1,5,E-02
LIMLP_RS10135	LIMLP_10150	1,65	3,1	2,7,E-03
LIMLP_RS10145	LIMLP_10160	-0,88	-1,8	2,3,E-04
LIMLP_RS10190	LIMLP_10205	1,14	2,2	1,8,E-06
LIMLP_RS10195	LIMLP_10210	0,97	2,0	5,8,E-03
LIMLP_RS10200	LIMLP_10215	-1,49	-2,8	8,2,E-07
LIMLP_RS10210	LIMLP_10225	1,33	2,5	2,4,E-04
LIMLP_RS10220	LIMLP_10235	-1,22	-2,3	7,6,E-03
LIMLP_RS10225	LIMLP_10240	-1,42	-2,7	1,4,E-08
LIMLP_RS10230	LIMLP_10245	-1,30	-2,5	1,2,E-08
LIMLP_RS10235	LIMLP_10250	0,67	1,6	4,3,E-02
LIMLP_RS10240	LIMLP_10255	1,89	3,7	1,5,E-05

LIMLP_RS10245	LIMLP_10260	1,18	2,3	3,4,E-05
LIMLP_RS10250	LIMLP_10265	-0,84	-1,8	2,3,E-03
LIMLP_RS10255	LIMLP_10270	0,94	1,9	2,0,E-02
LIMLP_RS10260	LIMLP_10275	1,64	3,1	1,1,E-02
LIMLP_RS10270	LIMLP_10285	0,90	1,9	5,0,E-03
LIMLP_RS10275	LIMLP_10290	0,95	1,9	6,2,E-03
LIMLP_RS10290	LIMLP_10305	1,08	2,1	1,7,E-04
LIMLP_RS10300	LIMLP_10315	-0,64	-1,6	2,1,E-02
LIMLP_RS10305	LIMLP_10320	-1,01	-2,0	1,1,E-03
LIMLP_RS10335	LIMLP_10350	-0,78	-1,7	2,3,E-02
LIMLP_RS10340	LIMLP_10355	-0,72	-1,6	1,0,E-02
LIMLP_RS10345	LIMLP_10360	-0,82	-1,8	1,0,E-03
LIMLP_RS10455	LIMLP_10470	-0,80	-1,7	5,6,E-03
LIMLP_RS10470	LIMLP_10490	-0,84	-1,8	7,0,E-03
LIMLP_RS10495	LIMLP_10515	-0,70	-1,6	4,3,E-02
LIMLP_RS10505	LIMLP_10525	-0,58	-1,5	3,8,E-02
LIMLP_RS10665	LIMLP_10685	-0,64	-1,6	9,8,E-03
LIMLP_RS10705	LIMLP_10725	-0,54	-1,5	6,6,E-03
LIMLP_RS10800	LIMLP_10820	-0,54	-1,5	3,3,E-02
LIMLP_RS10935	LIMLP_10960	0,94	1,9	4,1,E-02
LIMLP_RS10945	LIMLP_10970	3,49	11,3	3,5,E-04
LIMLP_RS10950	LIMLP_10975	3,27	9,6	5,8,E-04
LIMLP_RS10970	LIMLP_10995	-1,13	-2,2	2,4,E-02
LIMLP_RS10975	LIMLP_11000	-1,66	-3,2	2,9,E-03
LIMLP_RS10980	LIMLP_11005	-1,49	-2,8	9,8,E-04
LIMLP_RS11000	LIMLP_11025	-0,82	-1,8	6,5,E-04
LIMLP_RS11020	LIMLP_11045	0,85	1,8	3,6,E-02
LIMLP_RS11035	LIMLP_11060	1,26	2,4	9,4,E-07
LIMLP_RS11040	LIMLP_11065	1,25	2,4	1,8,E-08
LIMLP_RS11065	LIMLP_11090	1,02	2,0	7,8,E-04
LIMLP_RS11070	LIMLP_11095	1,03	2,0	2,7,E-02
LIMLP_RS11075	LIMLP_11100	1,41	2,7	1,7,E-03
LIMLP_RS11080	LIMLP_11105	1,11	2,2	2,6,E-05
LIMLP_RS11090	LIMLP_11115	-0,69	-1,6	1,8,E-03
LIMLP_RS11095	LIMLP_11120	-0,98	-2,0	9,8,E-05
LIMLP_RS11175	LIMLP_11200	0,67	1,6	2,4,E-02
LIMLP_RS11185	LIMLP_11210	0,84	1,8	6,8,E-03
LIMLP_RS11205	LIMLP_11230	-1,09	-2,1	1,0,E-02
LIMLP_RS11210	LIMLP_11235	-1,49	-2,8	2,3,E-05
LIMLP_RS11250	LIMLP_11275	-0,74	-1,7	5,5,E-04
LIMLP_RS11255	LIMLP_11280	-1,04	-2,1	2,4,E-06
LIMLP_RS11285	LIMLP_11310	-1,38	-2,6	1,3,E-06
LIMLP_RS11295	LIMLP_11320	-0,58	-1,5	4,3,E-02
LIMLP_RS11310	LIMLP_11335	-1,12	-2,2	5,7,E-04
LIMLP_RS11320	LIMLP_11345	-2,04	-4,1	4,8,E-07
LIMLP_RS11325	LIMLP_11350	-1,83	-3,6	2,1,E-06

LIMLP_RS11330	LIMLP_11355	-2,04	-4,1	3,5,E-07
LIMLP_RS11365	LIMLP_11390	1,09	2,1	4,2,E-02
LIMLP_RS11375	LIMLP_11400	2,22	4,6	9,4,E-05
LIMLP_RS11380	LIMLP_11405	2,24	4,7	3,6,E-04
LIMLP_RS11440	LIMLP_11465	0,54	1,4	4,2,E-02
LIMLP_RS11475	LIMLP_11500	-0,83	-1,8	3,0,E-03
LIMLP_RS11480	LIMLP_11505	-0,77	-1,7	7,6,E-03
LIMLP_RS11485	LIMLP_11510	-1,01	-2,0	1,4,E-04
LIMLP_RS11490	LIMLP_11515	-1,17	-2,3	1,6,E-03
LIMLP_RS11510	LIMLP_11535	2,64	6,2	1,1,E-06
LIMLP_RS11515	LIMLP_11540	2,74	6,7	5,0,E-07
LIMLP_RS11540	LIMLP_11565	0,86	1,8	2,7,E-02
LIMLP_RS11550	LIMLP_11575	0,83	1,8	2,0,E-02
LIMLP_RS11555	LIMLP_11580	1,07	2,1	8,3,E-03
LIMLP_RS11560	LIMLP_11585	1,21	2,3	1,2,E-03
LIMLP_RS11565	LIMLP_11590	1,33	2,5	8,9,E-05
LIMLP_RS11580	LIMLP_11605	-1,22	-2,3	5,4,E-06
LIMLP_RS11585	LIMLP_11610	-0,82	-1,8	1,6,E-03
LIMLP_RS11610	LIMLP_11635	-1,30	-2,5	2,2,E-03
LIMLP_RS11615	LIMLP_11640	-1,23	-2,3	4,9,E-03
LIMLP_RS11630	LIMLP_11655	3,01	8,1	1,3,E-06
LIMLP_RS11635	LIMLP_11660	1,60	3,0	9,6,E-04
LIMLP_RS11650	LIMLP_11675	-0,86	-1,8	1,8,E-02
LIMLP_RS11715	LIMLP_11740	1,10	2,1	4,6,E-02
LIMLP_RS11735	LIMLP_11760	-0,64	-1,6	2,6,E-02
LIMLP_RS11740	LIMLP_11765	0,95	1,9	5,6,E-07
LIMLP_RS11745	LIMLP_11770	1,17	2,3	1,1,E-05
LIMLP_RS11785	LIMLP_11810	-0,75	-1,7	2,3,E-02
LIMLP_RS11815	LIMLP_11840	-1,94	-3,8	7,3,E-06
LIMLP_RS11850	LIMLP_11875	-0,88	-1,8	3,8,E-02
LIMLP_RS11860	LIMLP_11885	-0,58	-1,5	1,4,E-02
LIMLP_RS11865	LIMLP_11890	-0,80	-1,7	5,9,E-04
LIMLP_RS11870	LIMLP_11895	-0,90	-1,9	1,1,E-03
LIMLP_RS11885	LIMLP_11910	0,84	1,8	1,2,E-02
LIMLP_RS11890	LIMLP_11915	0,81	1,8	5,7,E-03
LIMLP_RS11940	LIMLP_11965	2,18	4,5	2,0,E-15
LIMLP_RS11945	LIMLP_11970	-1,16	-2,2	2,6,E-05
LIMLP_RS11960	LIMLP_11985	-1,00	-2,0	1,6,E-02
LIMLP_RS11980	LIMLP_12005	1,90	3,7	5,5,E-08
LIMLP_RS11985	LIMLP_12010	1,06	2,1	1,1,E-02
LIMLP_RS11990	LIMLP_12015	-0,79	-1,7	4,2,E-03
LIMLP_RS12020	LIMLP_12045	-0,97	-2,0	8,3,E-04
LIMLP_RS12065	LIMLP_12090	0,80	1,7	1,1,E-02
LIMLP_RS12085	LIMLP_12110	0,93	1,9	1,4,E-02
LIMLP_RS12090	LIMLP_12115	1,23	2,3	1,2,E-03
LIMLP_RS12205	LIMLP_12230	-0,46	-1,4	1,5,E-02

LIMLP_RS12240	LIMLP_12265	-0,65	-1,6	9,5,E-04
LIMLP_RS12245	LIMLP_12270	-0,61	-1,5	4,4,E-03
LIMLP_RS12265	LIMLP_12290	-0,64	-1,6	9,5,E-03
LIMLP_RS12300	LIMLP_12325	1,88	3,7	3,7,E-10
LIMLP_RS12305	LIMLP_12330	1,49	2,8	4,2,E-14
LIMLP_RS12310	LIMLP_12335	0,86	1,8	4,3,E-02
LIMLP_RS12330	LIMLP_12355	-0,47	-1,4	1,9,E-02
LIMLP_RS12380	LIMLP_12405	0,91	1,9	1,2,E-02
LIMLP_RS12385	LIMLP_12410	-0,99	-2,0	3,9,E-05
LIMLP_RS12395	LIMLP_12420	0,94	1,9	1,0,E-02
LIMLP_RS12400	LIMLP_12425	2,22	4,7	4,8,E-18
LIMLP_RS12405	LIMLP_12430	1,28	2,4	3,4,E-12
LIMLP_RS12450	LIMLP_12480	1,34	2,5	1,8,E-05
LIMLP_RS12480	LIMLP_12510	-2,24	-4,7	4,4,E-02
LIMLP_RS12485	LIMLP_12515	0,86	1,8	1,4,E-02
LIMLP_RS12490	LIMLP_12520	0,88	1,8	3,0,E-03
LIMLP_RS23515	LIMLP_12545	1,36	2,6	1,3,E-03
LIMLP_RS12520	LIMLP_12550	0,70	1,6	5,2,E-03
LIMLP_RS12540	LIMLP_12570	0,86	1,8	9,7,E-05
LIMLP_RS12555	LIMLP_12585	-0,79	-1,7	1,5,E-02
LIMLP_RS12575	LIMLP_12605	-1,06	-2,1	9,5,E-06
LIMLP_RS12580	LIMLP_12610	-1,62	-3,1	1,0,E-05
LIMLP_RS12585	LIMLP_12615	-1,35	-2,6	2,7,E-05
LIMLP_RS12590	LIMLP_12620	-1,08	-2,1	1,7,E-02
LIMLP_RS12595	LIMLP_12625	-0,81	-1,7	1,8,E-02
LIMLP_RS12640	LIMLP_12670	1,53	2,9	5,5,E-09
LIMLP_RS12655	LIMLP_12685	-1,07	-2,1	1,7,E-02
LIMLP_RS12730	LIMLP_12760	-0,82	-1,8	8,1,E-04
LIMLP_RS12735	LIMLP_12765	-0,67	-1,6	2,3,E-03
LIMLP_RS12750	LIMLP_12780	0,77	1,7	5,5,E-03
LIMLP_RS12760	LIMLP_12790	0,79	1,7	1,7,E-02
LIMLP_RS12775	LIMLP_12805	1,68	3,2	1,3,E-07
LIMLP_RS12800	LIMLP_12830	-0,85	-1,8	1,2,E-02
LIMLP_RS12825	LIMLP_12855	0,90	1,9	3,3,E-04
LIMLP_RS12855	LIMLP_12885	-1,10	-2,1	4,9,E-03
LIMLP_RS12890	LIMLP_12920	0,67	1,6	3,3,E-02
LIMLP_RS12925	LIMLP_12955	-0,67	-1,6	5,2,E-03
LIMLP_RS12980	LIMLP_13010	1,64	3,1	9,9,E-03
LIMLP_RS12985	LIMLP_13015	1,70	3,2	1,7,E-02
LIMLP_RS12990	LIMLP_13020	1,66	3,2	1,0,E-02
LIMLP_RS12995	LIMLP_13025	1,68	3,2	1,2,E-02
LIMLP_RS13000	LIMLP_13030	1,67	3,2	2,0,E-02
LIMLP_RS13005	LIMLP_13035	1,69	3,2	1,7,E-02
LIMLP_RS13010	LIMLP_13040	1,90	3,7	4,4,E-02
LIMLP_RS13020	LIMLP_13050	1,80	3,5	4,6,E-02
LIMLP_RS13030	LIMLP_13060	1,79	3,5	3,4,E-02

LIMLP_RS13040	LIMLP_13070	1,89	3,7	4,3,E-02
LIMLP_RS13045	LIMLP_13075	1,89	3,7	3,3,E-02
LIMLP_RS13050	LIMLP_13080	1,97	3,9	2,6,E-02
LIMLP_RS13055	LIMLP_13085	1,78	3,4	3,6,E-02
LIMLP_RS13060	LIMLP_13090	1,98	3,9	6,1,E-03
LIMLP_RS13085	LIMLP_13115	-0,63	-1,5	2,5,E-03
LIMLP_RS13090	LIMLP_13120	-0,54	-1,5	4,2,E-02
LIMLP_RS13110	LIMLP_13140	-0,55	-1,5	2,5,E-02
LIMLP_RS13135	LIMLP_13165	2,89	7,4	2,4,E-03
LIMLP_RS13140	LIMLP_13170	2,33	5,0	1,2,E-06
LIMLP_RS13145	LIMLP_13175	0,49	1,4	3,0,E-02
LIMLP_RS13150	LIMLP_13180	0,88	1,8	3,9,E-05
LIMLP_RS13160	LIMLP_13190	-0,64	-1,6	2,4,E-02
LIMLP_RS13170	LIMLP_13200	1,16	2,2	6,1,E-04
LIMLP_RS13175	LIMLP_13205	1,64	3,1	3,3,E-06
LIMLP_RS13185	LIMLP_13215	-0,47	-1,4	4,1,E-02
LIMLP_RS13205	LIMLP_13235	-1,14	-2,2	1,4,E-04
LIMLP_RS13220	LIMLP_13250	-0,66	-1,6	1,6,E-03
LIMLP_RS13250	LIMLP_13280	-1,14	-2,2	4,6,E-04
LIMLP_RS13255	LIMLP_13285	-1,20	-2,3	1,2,E-03
LIMLP_RS13260	LIMLP_13290	-0,88	-1,8	3,4,E-02
LIMLP_RS13265	LIMLP_13295	-0,87	-1,8	3,4,E-02
LIMLP_RS13270	LIMLP_13300	-0,82	-1,8	4,0,E-02
LIMLP_RS13300	LIMLP_13330	0,65	1,6	2,0,E-02
LIMLP_RS13305	LIMLP_13335	1,23	2,3	2,1,E-03
LIMLP_RS13340	LIMLP_13370	-1,04	-2,1	7,6,E-06
LIMLP_RS13350	LIMLP_13380	-0,63	-1,6	1,8,E-02
LIMLP_RS13405	LIMLP_13435	-0,74	-1,7	3,1,E-02
LIMLP_RS13410	LIMLP_13440	-0,73	-1,7	2,4,E-02
LIMLP_RS13430	LIMLP_13460	0,45	1,4	3,1,E-02
LIMLP_RS13435	LIMLP_13465	0,62	1,5	6,4,E-03
LIMLP_RS13440	LIMLP_13470	0,49	1,4	3,4,E-02
LIMLP_RS13445	LIMLP_13475	0,54	1,5	3,1,E-03
LIMLP_RS13450	LIMLP_13480	0,58	1,5	8,4,E-03
LIMLP_RS13475	LIMLP_13505	1,19	2,3	3,4,E-04
LIMLP_RS13480	LIMLP_13510	0,99	2,0	1,9,E-02
LIMLP_RS13490	LIMLP_13520	1,08	2,1	1,1,E-04
LIMLP_RS13540	LIMLP_13570	-1,13	-2,2	1,3,E-05
LIMLP_RS13550	LIMLP_13580	-1,36	-2,6	8,5,E-07
LIMLP_RS13555	LIMLP_13585	-1,07	-2,1	4,8,E-03
LIMLP_RS13560	LIMLP_13590	-1,04	-2,1	3,9,E-06
LIMLP_RS13565	LIMLP_13595	-0,98	-2,0	1,0,E-02
LIMLP_RS13585	LIMLP_13615	0,76	1,7	1,3,E-04
LIMLP_RS13590	LIMLP_13620	2,98	7,9	1,8,E-04
LIMLP_RS13605	LIMLP_13635	1,93	3,8	2,5,E-11
LIMLP_RS13610	LIMLP_13640	1,36	2,6	1,6,E-05

LIMLP_RS13615	LIMLP_13645	1,18	2,3	1,5,E-03
LIMLP_RS13620	LIMLP_13650	2,23	4,7	6,4,E-06
LIMLP_RS13625	LIMLP_13655	2,10	4,3	1,2,E-08
LIMLP_RS13635	LIMLP_13665	1,04	2,1	6,4,E-04
LIMLP_RS13640	LIMLP_13670	3,44	10,9	4,3,E-26
LIMLP_RS13655	LIMLP_13685	-0,86	-1,8	2,5,E-03
LIMLP_RS13660	LIMLP_13690	-0,71	-1,6	4,5,E-03
LIMLP_RS13665	LIMLP_13695	-0,92	-1,9	1,2,E-04
LIMLP_RS13670	LIMLP_13700	-1,21	-2,3	3,1,E-05
LIMLP_RS13675	LIMLP_13705	0,83	1,8	7,5,E-03
LIMLP_RS13685	LIMLP_13715	-1,35	-2,6	9,6,E-06
LIMLP_RS13690	LIMLP_13720	-1,96	-3,9	7,7,E-06
LIMLP_RS13695	LIMLP_13725	-2,16	-4,5	9,4,E-07
LIMLP_RS13700	LIMLP_13730	-2,19	-4,6	1,8,E-05
LIMLP_RS13720	LIMLP_13750	-0,98	-2,0	4,0,E-02
LIMLP_RS13725	LIMLP_13755	-1,04	-2,1	3,2,E-03
LIMLP_RS13730	LIMLP_13760	-1,04	-2,1	2,4,E-08
LIMLP_RS13735	LIMLP_13765	1,01	2,0	4,7,E-02
LIMLP_RS13745	LIMLP_13775	-0,96	-2,0	8,4,E-04
LIMLP_RS13750	LIMLP_13780	-1,24	-2,4	2,2,E-09
LIMLP_RS13790	LIMLP_13820	-1,14	-2,2	4,4,E-04
LIMLP_RS13800	LIMLP_13830	-1,30	-2,5	1,4,E-04
LIMLP_RS13815	LIMLP_13845	-1,00	-2,0	1,1,E-02
LIMLP_RS13850	LIMLP_13880	2,00	4,0	7,0,E-19
LIMLP_RS13865	LIMLP_13895	-0,66	-1,6	1,0,E-02
LIMLP_RS13870	LIMLP_13900	-0,67	-1,6	3,8,E-02
LIMLP_RS13875	LIMLP_13905	0,76	1,7	1,2,E-02
LIMLP_RS13880	LIMLP_13910	0,41	1,3	8,2,E-03
LIMLP_RS13900	LIMLP_13930	-0,79	-1,7	2,2,E-02
LIMLP_RS13920	LIMLP_13950	-1,00	-2,0	2,5,E-05
LIMLP_RS13925	LIMLP_13955	-0,74	-1,7	1,6,E-03
LIMLP_RS13975	LIMLP_14005	-0,96	-1,9	5,1,E-07
LIMLP_RS14035	LIMLP_14065	1,08	2,1	3,5,E-02
LIMLP_RS14045	LIMLP_14075	-0,75	-1,7	2,1,E-02
LIMLP_RS14050	LIMLP_14080	0,61	1,5	6,9,E-03
LIMLP_RS14105	LIMLP_14135	0,57	1,5	7,8,E-03
LIMLP_RS14110	LIMLP_14140	0,51	1,4	2,3,E-02
LIMLP_RS14115	LIMLP_14145	0,68	1,6	8,0,E-04
LIMLP_RS14125	LIMLP_14155	1,13	2,2	1,1,E-02
LIMLP_RS14155	LIMLP_14185	-0,87	-1,8	6,9,E-04
LIMLP_RS14165	LIMLP_14195	-1,09	-2,1	2,6,E-03
LIMLP_RS14170	LIMLP_14200	-1,38	-2,6	5,5,E-05
LIMLP_RS14175	LIMLP_14205	-1,29	-2,4	1,1,E-03
LIMLP_RS14185	LIMLP_14215	-1,13	-2,2	2,3,E-03
LIMLP_RS14190	LIMLP_14220	-0,74	-1,7	1,0,E-02
LIMLP_RS14200	LIMLP_14230	-1,41	-2,7	1,1,E-09

LIMLP_RS14205	LIMLP_14235	-0,81	-1,7	1,1,E-02
LIMLP_RS14240	LIMLP_14275	-0,88	-1,8	1,4,E-02
LIMLP_RS14245	LIMLP_14280	-1,00	-2,0	1,2,E-06
LIMLP_RS14250	LIMLP_14285	-0,58	-1,5	2,4,E-02
LIMLP_RS14255	LIMLP_14290	-1,57	-3,0	3,0,E-05
LIMLP_RS14260	LIMLP_14295	1,06	2,1	1,1,E-02
LIMLP_RS14265	LIMLP_14300	1,10	2,1	1,0,E-06
LIMLP_RS14285	LIMLP_14320	-0,59	-1,5	3,9,E-02
LIMLP_RS14315	LIMLP_14350	-0,71	-1,6	3,0,E-02
LIMLP_RS14350	LIMLP_14385	-0,85	-1,8	7,7,E-04
LIMLP_RS14365	LIMLP_14400	0,99	2,0	1,1,E-02
LIMLP_RS14380	LIMLP_14415	1,24	2,4	2,1,E-02
LIMLP_RS14425	LIMLP_14460	-1,21	-2,3	1,0,E-03
LIMLP_RS14430	LIMLP_14465	1,00	2,0	1,1,E-02
LIMLP_RS14460	LIMLP_14495	0,88	1,8	4,7,E-03
LIMLP_RS14465	LIMLP_14500	0,80	1,7	1,9,E-02
LIMLP_RS14515	LIMLP_14550	1,32	2,5	7,5,E-08
LIMLP_RS14520	LIMLP_14555	1,87	3,7	7,5,E-08
LIMLP_RS14525	LIMLP_14560	2,26	4,8	1,5,E-06
LIMLP_RS14530	LIMLP_14565	1,22	2,3	4,1,E-06
LIMLP_RS14535	LIMLP_14570	1,15	2,2	3,7,E-04
LIMLP_RS14540	LIMLP_14575	0,76	1,7	4,6,E-02
LIMLP_RS14545	LIMLP_14580	0,71	1,6	6,9,E-04
LIMLP_RS14555	LIMLP_14590	-1,65	-3,1	1,6,E-14
LIMLP_RS14580	LIMLP_14615	-0,72	-1,6	1,0,E-03
LIMLP_RS14585	LIMLP_14620	-1,24	-2,4	1,8,E-06
LIMLP_RS14590	LIMLP_14625	-1,08	-2,1	2,0,E-08
LIMLP_RS14595	LIMLP_14630	-1,18	-2,3	5,8,E-07
LIMLP_RS14600	LIMLP_14635	-1,26	-2,4	2,1,E-11
LIMLP_RS14605	LIMLP_14640	-1,70	-3,2	1,6,E-09
LIMLP_RS14615	LIMLP_14650	1,38	2,6	3,5,E-08
LIMLP_RS14625	LIMLP_14660	-0,54	-1,5	1,7,E-02
LIMLP_RS14630	LIMLP_14665	-0,61	-1,5	1,5,E-02
LIMLP_RS14640	LIMLP_14675	-1,17	-2,2	2,1,E-02
LIMLP_RS14665	LIMLP_14700	1,43	2,7	1,6,E-08
LIMLP_RS14670	LIMLP_14705	0,74	1,7	1,5,E-05
LIMLP_RS14685	LIMLP_14720	0,83	1,8	3,1,E-02
LIMLP_RS14690	LIMLP_14725	-0,99	-2,0	2,2,E-02
LIMLP_RS14700	LIMLP_14735	-0,83	-1,8	4,1,E-02
LIMLP_RS14740	LIMLP_14775	-0,38	-1,3	2,7,E-02
LIMLP_RS14745	LIMLP_14780	0,89	1,9	2,0,E-02
LIMLP_RS14770	LIMLP_14805	-0,50	-1,4	8,7,E-03
LIMLP_RS14820	LIMLP_14855	-0,39	-1,3	3,7,E-02
LIMLP_RS14830	LIMLP_14865	1,32	2,5	9,6,E-07
LIMLP_RS14865	LIMLP_14900	1,26	2,4	1,6,E-05
LIMLP_RS14870	LIMLP_14905	1,70	3,2	7,0,E-05

LIMLP_RS14895	LIMLP_14930	-1,47	-2,8	4,4,E-02
LIMLP_RS14900	LIMLP_14935	1,59	3,0	3,3,E-06
LIMLP_RS14905	LIMLP_14940	1,77	3,4	5,0,E-07
LIMLP_RS14910	LIMLP_14945	1,94	3,8	4,3,E-07
LIMLP_RS14915	LIMLP_14950	-0,84	-1,8	2,7,E-02
LIMLP_RS14920	LIMLP_14955	-0,84	-1,8	1,7,E-04
LIMLP_RS14930	LIMLP_14965	-0,62	-1,5	1,2,E-02
LIMLP_RS14940	LIMLP_14975	-2,39	-5,3	6,4,E-25
LIMLP_RS14990	LIMLP_15025	-0,98	-2,0	3,4,E-04
LIMLP_RS15000	LIMLP_15035	1,04	2,1	3,6,E-03
LIMLP_RS15010	LIMLP_15045	0,79	1,7	4,0,E-02
LIMLP_RS15015	LIMLP_15050	-1,04	-2,0	9,2,E-07
LIMLP_RS15025	LIMLP_15065	-0,51	-1,4	4,3,E-02
LIMLP_RS15055	LIMLP_15095	-0,82	-1,8	2,5,E-02
LIMLP_RS15065	LIMLP_15105	1,79	3,5	7,0,E-08
LIMLP_RS15070	LIMLP_15110	1,83	3,6	9,1,E-06
LIMLP_RS15075	LIMLP_15115	1,98	3,9	3,6,E-04
LIMLP_RS15080	LIMLP_15120	1,88	3,7	8,0,E-04
LIMLP_RS15090	LIMLP_15130	0,98	2,0	3,4,E-02
LIMLP_RS15095	LIMLP_15135	-0,86	-1,8	1,8,E-02
LIMLP_RS15130	LIMLP_15170	0,96	1,9	1,0,E-02
LIMLP_RS15155	LIMLP_15195	1,30	2,5	2,5,E-05
LIMLP_RS15165	LIMLP_15205	-0,83	-1,8	4,8,E-03
LIMLP_RS15170	LIMLP_15210	-0,94	-1,9	2,3,E-03
LIMLP_RS15175	LIMLP_15215	-0,76	-1,7	5,9,E-03
LIMLP_RS15180	LIMLP_15220	-1,55	-2,9	1,0,E-02
LIMLP_RS15185	LIMLP_15225	-1,10	-2,1	3,2,E-04
LIMLP_RS15210	LIMLP_15250	0,96	1,9	1,5,E-02
LIMLP_RS15245	LIMLP_15285	0,65	1,6	3,7,E-02
LIMLP_RS15250	LIMLP_15290	1,33	2,5	8,2,E-04
LIMLP_RS15255	LIMLP_15295	0,64	1,6	4,4,E-04
LIMLP_RS15260	LIMLP_15300	0,66	1,6	4,6,E-03
LIMLP_RS15265	LIMLP_15305	0,67	1,6	4,6,E-02
LIMLP_RS15295	LIMLP_15335	-1,53	-2,9	3,6,E-02
LIMLP_RS15305	LIMLP_15345	-1,97	-3,9	1,6,E-04
LIMLP_RS15380	LIMLP_15420	2,59	6,0	6,7,E-05
LIMLP_RS15385	LIMLP_15425	2,56	5,9	1,2,E-03
LIMLP_RS15415	LIMLP_15455	-0,57	-1,5	1,2,E-02
LIMLP_RS15440	LIMLP_15480	-0,95	-1,9	2,4,E-04
LIMLP_RS15445	LIMLP_15485	-0,80	-1,7	1,8,E-03
LIMLP_RS15450	LIMLP_15490	0,86	1,8	1,6,E-03
LIMLP_RS15455	LIMLP_15495	-1,14	-2,2	1,7,E-02
LIMLP_RS15470	LIMLP_15510	-0,70	-1,6	3,6,E-02
LIMLP_RS15475	LIMLP_15515	-0,78	-1,7	5,2,E-04
LIMLP_RS15480	LIMLP_15520	-0,95	-1,9	2,8,E-05
LIMLP_RS15485	LIMLP_15525	-1,25	-2,4	7,8,E-10

LIMLP_RS15515	LIMLP_15555	-0,78	-1,7	8,7,E-03
LIMLP_RS15550	LIMLP_15590	-0,87	-1,8	2,4,E-02
LIMLP_RS15555	LIMLP_15595	-0,89	-1,9	5,9,E-04
LIMLP_RS15575	LIMLP_15615	-0,40	-1,3	4,9,E-02
LIMLP_RS15630	LIMLP_15670	-0,80	-1,7	3,4,E-02
LIMLP_RS15650	LIMLP_15690	-0,84	-1,8	1,6,E-03
LIMLP_RS15655	LIMLP_15695	-0,46	-1,4	3,1,E-03
LIMLP_RS15670	LIMLP_15710	-1,07	-2,1	6,0,E-03
LIMLP_RS15700	LIMLP_15740	-0,48	-1,4	2,8,E-02
LIMLP_RS15715	LIMLP_15755	-1,29	-2,4	8,2,E-05
LIMLP_RS15735	LIMLP_15775	-0,61	-1,5	2,5,E-03
LIMLP_RS15760	LIMLP_15800	-1,08	-2,1	3,2,E-04
LIMLP_RS15765	LIMLP_15805	-0,84	-1,8	2,8,E-02
LIMLP_RS15820	LIMLP_15860	-1,13	-2,2	5,9,E-05
LIMLP_RS15850	LIMLP_15890	1,34	2,5	1,6,E-02
LIMLP_RS15895	LIMLP_15935	-0,82	-1,8	3,1,E-02
LIMLP_RS15915	LIMLP_15955	-0,99	-2,0	6,0,E-03
LIMLP_RS15950	LIMLP_15990	0,86	1,8	1,0,E-02
LIMLP_RS15965	LIMLP_16005	-0,72	-1,7	6,0,E-03
LIMLP_RS15970	LIMLP_16010	-0,64	-1,6	3,0,E-03
LIMLP_RS15975	LIMLP_16015	2,47	5,5	4,2,E-14
LIMLP_RS15990	LIMLP_16030	0,46	1,4	3,1,E-02
LIMLP_RS15995	LIMLP_16035	-0,84	-1,8	2,4,E-03
LIMLP_RS16045	LIMLP_16085	0,50	1,4	1,2,E-02
LIMLP_RS16090	LIMLP_16130	-1,43	-2,7	6,5,E-08
LIMLP_RS16095	LIMLP_16135	-1,55	-2,9	2,4,E-13
LIMLP_RS16110	LIMLP_16150	-1,25	-2,4	7,6,E-07
LIMLP_RS16115	LIMLP_16155	-1,05	-2,1	4,4,E-10
LIMLP_RS16120	LIMLP_16160	-0,75	-1,7	1,6,E-04
LIMLP_RS16145	LIMLP_16185	-0,67	-1,6	2,8,E-02
LIMLP_RS16150	LIMLP_16190	-0,62	-1,5	9,8,E-03
LIMLP_RS16170	LIMLP_16210	0,74	1,7	1,1,E-03
LIMLP_RS16190	LIMLP_16230	0,85	1,8	1,5,E-02
LIMLP_RS16205	LIMLP_16245	-1,16	-2,2	8,1,E-03
LIMLP_RS16225	LIMLP_16265	1,40	2,6	2,1,E-04
LIMLP_RS16235	LIMLP_16275	-0,84	-1,8	3,8,E-06
LIMLP_RS16240	LIMLP_16280	-1,07	-2,1	8,6,E-06
LIMLP_RS16250	LIMLP_16290	-0,60	-1,5	3,1,E-02
LIMLP_RS16255	LIMLP_16295	-1,00	-2,0	7,9,E-03
LIMLP_RS16260	LIMLP_16300	-0,93	-1,9	4,6,E-04
LIMLP_RS16280	LIMLP_16320	-1,41	-2,7	1,8,E-03
LIMLP_RS16285	LIMLP_16325	-0,99	-2,0	4,2,E-02
LIMLP_RS16290	LIMLP_16330	-0,72	-1,6	1,4,E-02
LIMLP_RS16320	LIMLP_16360	-1,12	-2,2	3,9,E-02
LIMLP_RS16325	LIMLP_16365	-0,65	-1,6	1,0,E-02
LIMLP_RS16330	LIMLP_16370	-0,95	-1,9	2,1,E-04

LIMLP_RS16335	LIMLP_16375	-1,84	-3,6	7,9,E-14
LIMLP_RS16345	LIMLP_16385	-1,33	-2,5	7,8,E-06
LIMLP_RS16350	LIMLP_16390	-1,46	-2,7	6,8,E-06
LIMLP_RS16365	LIMLP_16405	-0,64	-1,6	7,9,E-03
LIMLP_RS16375	LIMLP_16415	-0,63	-1,5	9,1,E-03
LIMLP_RS16425	LIMLP_16465	1,18	2,3	8,4,E-03
LIMLP_RS16430	LIMLP_16470	-1,38	-2,6	1,5,E-08
LIMLP_RS16440	LIMLP_16480	-0,62	-1,5	2,5,E-02
LIMLP_RS16445	LIMLP_16485	0,50	1,4	3,0,E-03
LIMLP_RS16465	LIMLP_16505	1,81	3,5	2,8,E-13
LIMLP_RS16470	LIMLP_16510	1,68	3,2	4,7,E-14
LIMLP_RS16475	LIMLP_16515	-0,82	-1,8	2,9,E-02
LIMLP_RS16485	LIMLP_16525	0,86	1,8	2,5,E-02
LIMLP_RS16495	LIMLP_16535	0,74	1,7	4,9,E-02
LIMLP_RS16500	LIMLP_16540	1,44	2,7	8,5,E-03
LIMLP_RS16515	LIMLP_16555	1,56	2,9	4,0,E-04
LIMLP_RS16525	LIMLP_16565	-0,79	-1,7	1,1,E-02
LIMLP_RS16530	LIMLP_16570	-1,00	-2,0	6,9,E-04
LIMLP_RS16545	LIMLP_16585	-0,79	-1,7	5,3,E-03
LIMLP_RS16550	LIMLP_16590	-0,71	-1,6	2,3,E-03
LIMLP_RS16575	LIMLP_16615	-1,26	-2,4	5,8,E-05
LIMLP_RS16610	LIMLP_16650	-0,58	-1,5	1,5,E-02
LIMLP_RS16615	LIMLP_16655	-0,57	-1,5	4,2,E-03
LIMLP_RS16630	LIMLP_16670	0,94	1,9	2,0,E-04
LIMLP_RS23555	LIMLP_16695	1,33	2,5	3,2,E-04
LIMLP_RS16660	LIMLP_16700	0,66	1,6	4,9,E-03
LIMLP_RS16665	LIMLP_16705	0,77	1,7	3,7,E-02
LIMLP_RS16690	LIMLP_16730	-0,91	-1,9	1,3,E-05
LIMLP_RS16695	LIMLP_16735	-0,47	-1,4	1,6,E-03
LIMLP_RS16700	LIMLP_16740	-0,50	-1,4	9,0,E-03
LIMLP_RS16725	LIMLP_16765	1,64	3,1	3,3,E-06
LIMLP_RS16760	LIMLP_16800	2,00	4,0	6,5,E-09
LIMLP_RS16765	LIMLP_16805	1,97	3,9	3,0,E-12
LIMLP_RS16770	LIMLP_16810	1,91	3,8	4,5,E-14
LIMLP_RS16775	LIMLP_16815	1,33	2,5	2,4,E-08
LIMLP_RS16830	LIMLP_16870	2,55	5,9	6,2,E-22
LIMLP_RS16845	LIMLP_16885	0,93	1,9	1,9,E-02
LIMLP_RS16865	LIMLP_16905	0,79	1,7	2,6,E-02
LIMLP_RS16870	LIMLP_16910	1,43	2,7	2,7,E-02
LIMLP_RS16930	LIMLP_16970	-1,29	-2,4	2,9,E-04
LIMLP_RS16940	LIMLP_16980	-0,85	-1,8	2,8,E-03
LIMLP_RS16945	LIMLP_16985	-0,88	-1,8	4,2,E-04
LIMLP_RS16950	LIMLP_16990	-1,66	-3,2	2,8,E-05
LIMLP_RS16970	LIMLP_17010	-0,81	-1,8	1,8,E-02
LIMLP_RS16980	LIMLP_17020	-1,19	-2,3	2,5,E-03
LIMLP_RS17000	LIMLP_17040	-0,61	-1,5	2,2,E-02

LIMLP_RS17125	LIMLP_17165	-0,50	-1,4	4,9,E-02
LIMLP_RS17155	LIMLP_17195	-1,61	-3,0	2,8,E-13
LIMLP_RS17160	LIMLP_17200	-1,69	-3,2	1,2,E-09
LIMLP_RS17170	LIMLP_17210	0,96	1,9	5,8,E-04
LIMLP_RS17175	LIMLP_17215	0,95	1,9	2,6,E-03
LIMLP_RS17180	LIMLP_17220	0,98	2,0	3,5,E-03
LIMLP_RS17185	LIMLP_17225	0,96	2,0	2,3,E-03
LIMLP_RS17190	LIMLP_17230	1,14	2,2	2,1,E-03
LIMLP_RS17205	LIMLP_17245	1,03	2,0	3,6,E-02
LIMLP_RS17210	LIMLP_17250	1,51	2,9	7,8,E-06
LIMLP_RS17220	LIMLP_17260	-0,76	-1,7	4,6,E-03
LIMLP_RS17230	LIMLP_17270	-0,78	-1,7	2,1,E-02
LIMLP_RS17235	LIMLP_17275	-0,75	-1,7	2,4,E-02
LIMLP_RS17245	LIMLP_17285	-0,94	-1,9	2,9,E-04
LIMLP_RS17250	LIMLP_17290	1,15	2,2	1,9,E-02
LIMLP_RS17285	LIMLP_17325	-0,97	-2,0	1,3,E-03
LIMLP_RS17295	LIMLP_17335	1,51	2,8	2,8,E-04
LIMLP_RS17315	LIMLP_17355	-0,88	-1,8	3,5,E-05
LIMLP_RS17330	LIMLP_17370	-0,86	-1,8	9,0,E-03
LIMLP_RS17335	LIMLP_17375	0,98	2,0	5,7,E-05
LIMLP_RS17360	LIMLP_17400	1,20	2,3	5,0,E-05
LIMLP_RS17370	LIMLP_17410	1,34	2,5	3,4,E-02
LIMLP_RS17375	LIMLP_17415	0,62	1,5	3,5,E-02
LIMLP_RS17380	LIMLP_17420	2,26	4,8	1,4,E-02
LIMLP_RS17440	LIMLP_17480	-0,98	-2,0	8,1,E-03
LIMLP_RS17450	LIMLP_17490	-1,39	-2,6	2,1,E-03
LIMLP_RS17460	LIMLP_17500	-0,85	-1,8	1,1,E-02
LIMLP_RS17465	LIMLP_17505	0,67	1,6	1,4,E-02
LIMLP_RS17495	LIMLP_17535	-0,72	-1,6	5,7,E-03
LIMLP_RS17515	LIMLP_17555	-1,20	-2,3	2,3,E-13
LIMLP_RS17520	LIMLP_17560	-0,93	-1,9	2,9,E-05
LIMLP_RS17525	LIMLP_17565	1,07	2,1	4,5,E-02
LIMLP_RS17530	LIMLP_17570	-0,88	-1,8	3,1,E-03
LIMLP_RS17545	LIMLP_17585	-0,88	-1,8	1,3,E-03
LIMLP_RS17550	LIMLP_17590	-1,11	-2,2	1,7,E-06
LIMLP_RS17580	LIMLP_17620	-0,75	-1,7	1,7,E-03
LIMLP_RS17590	LIMLP_17630	-0,90	-1,9	1,2,E-02
LIMLP_RS17595	LIMLP_17635	-0,54	-1,5	2,3,E-02
LIMLP_RS17620	LIMLP_17660	-2,04	-4,1	1,7,E-06
LIMLP_RS17625	LIMLP_17665	-1,09	-2,1	3,3,E-03
LIMLP_RS17630	LIMLP_17670	-0,78	-1,7	1,1,E-02
LIMLP_RS17660	LIMLP_17700	1,04	2,1	4,3,E-02
LIMLP_RS17665	LIMLP_17705	0,79	1,7	2,7,E-02
LIMLP_RS17670	LIMLP_17710	-1,04	-2,1	8,8,E-10
LIMLP_RS17675	LIMLP_17715	-0,61	-1,5	1,4,E-02
LIMLP_RS17725	LIMLP_17765	0,57	1,5	1,4,E-02

LIMLP_RS17730	LIMLP_17770	0,66	1,6	4,8,E-04
LIMLP_RS17735	LIMLP_17775	0,75	1,7	2,7,E-03
LIMLP_RS17745	LIMLP_17785	-0,87	-1,8	2,3,E-04
LIMLP_RS17880	LIMLP_17920	0,67	1,6	6,7,E-03
LIMLP_RS17885	LIMLP_17925	-1,09	-2,1	6,5,E-03
LIMLP_RS17895	LIMLP_17935	1,57	3,0	2,5,E-03
LIMLP_RS17965	LIMLP_18005	-0,99	-2,0	9,0,E-06
LIMLP_RS18010	LIMLP_18050	-0,44	-1,4	1,7,E-02
LIMLP_RS18020	LIMLP_18060	0,36	1,3	3,1,E-02
LIMLP_RS18025	LIMLP_18065	0,81	1,7	3,6,E-05
LIMLP_RS18030	LIMLP_18070	1,23	2,3	2,7,E-09
LIMLP_RS18035	LIMLP_18075	0,85	1,8	1,2,E-02
LIMLP_RS18040	LIMLP_18080	1,10	2,1	2,1,E-05
LIMLP_RS18055	LIMLP_18095	-1,26	-2,4	9,1,E-04
LIMLP_RS18090	LIMLP_18130	-1,01	-2,0	1,6,E-03
LIMLP_RS18095	LIMLP_18135	1,34	2,5	1,4,E-05
LIMLP_RS18100	LIMLP_18140	1,34	2,5	5,1,E-10
LIMLP_RS18120	LIMLP_18160	-0,72	-1,6	1,9,E-02
LIMLP_RS18130	LIMLP_18170	-1,27	-2,4	1,6,E-10
LIMLP_RS18135	LIMLP_18175	-0,89	-1,8	3,5,E-05
LIMLP_RS18140	LIMLP_18180	-1,06	-2,1	4,9,E-03
LIMLP_RS18145	LIMLP_18185	-1,03	-2,0	7,0,E-04
LIMLP_RS18160	LIMLP_18200	1,35	2,5	7,6,E-05
LIMLP_RS18165	LIMLP_18205	2,49	5,6	1,2,E-09
LIMLP_RS18175	LIMLP_18215	1,11	2,2	1,1,E-02
LIMLP_RS18195	LIMLP_18235	1,23	2,3	2,4,E-02
LIMLP_RS18200	LIMLP_18240	1,35	2,5	2,1,E-02
LIMLP_RS18235	LIMLP_18275	0,91	1,9	3,2,E-04
LIMLP_RS18255	LIMLP_18295	-0,63	-1,6	3,6,E-03
LIMLP_RS18260	LIMLP_18300	-0,47	-1,4	4,9,E-02
LIMLP_RS18265	LIMLP_18305	1,03	2,0	2,8,E-03
LIMLP_RS18340	LIMLP_18380	-0,97	-2,0	4,8,E-03
LIMLP_RS18345	LIMLP_18385	0,61	1,5	3,3,E-03
LIMLP_RS18360	LIMLP_18400	1,17	2,3	2,1,E-05
LIMLP_RS18370	LIMLP_18410	-1,15	-2,2	2,6,E-02
LIMLP_RS18400	LIMLP_18440	0,50	1,4	1,8,E-02
LIMLP_RS18405	LIMLP_18445	2,11	4,3	1,4,E-05
LIMLP_RS18410	LIMLP_18450	1,72	3,3	7,6,E-06
LIMLP_RS18415	LIMLP_18455	0,88	1,8	1,2,E-03
LIMLP_RS18420	LIMLP_18460	1,21	2,3	1,5,E-03
LIMLP_RS18425	LIMLP_18465	1,41	2,7	6,8,E-03
LIMLP_RS18450	LIMLP_18490	0,90	1,9	4,7,E-02
LIMLP_RS18460	LIMLP_18500	1,12	2,2	1,9,E-02
LIMLP_RS18465	LIMLP_18505	0,91	1,9	3,4,E-02
LIMLP_RS18470	LIMLP_18510	1,21	2,3	2,6,E-03
LIMLP_RS18475	LIMLP_18515	1,21	2,3	1,6,E-03

LIMLP_RS18490	LIMLP_18530	1,05	2,1	6,3,E-03
LIMLP_RS18520	LIMLP_18560	1,84	3,6	4,6,E-07
LIMLP_RS18570	LIMLP_18610	0,63	1,5	4,1,E-03
LIMLP_RS18590	LIMLP_18630	1,10	2,1	2,2,E-04
LIMLP_RS18625	LIMLP_18665	-1,11	-2,2	1,3,E-03
LIMLP_RS18630	LIMLP_18670	-1,53	-2,9	5,1,E-05
LIMLP_RS18635	LIMLP_18675	-1,07	-2,1	6,7,E-04
LIMLP_RS18640	LIMLP_18680	-1,16	-2,2	4,9,E-03
LIMLP_RS18650	LIMLP_18690	-0,66	-1,6	2,6,E-02
LIMLP_RS18655	LIMLP_18695	-1,13	-2,2	4,5,E-03
LIMLP_RS18660	LIMLP_18700	-1,50	-2,8	5,4,E-04
LIMLP_RS18665	LIMLP_18705	-1,11	-2,2	1,4,E-02
LIMLP_RS18680	LIMLP_18720	-0,81	-1,8	7,0,E-03
LIMLP_RS18685	LIMLP_18725	-1,59	-3,0	1,1,E-04
LIMLP_RS18690	LIMLP_18730	-1,33	-2,5	2,7,E-08
LIMLP_RS18700	LIMLP_18740	1,03	2,0	5,7,E-08
LIMLP_RS18710	LIMLP_18750	0,63	1,6	7,3,E-04
LIMLP_RS18720	LIMLP_18760	-1,19	-2,3	1,7,E-03
LIMLP_RS18745	LIMLP_18785	0,60	1,5	2,0,E-02
LIMLP_RS18775	LIMLP_18815	-1,55	-2,9	3,0,E-07
LIMLP_RS18805	LIMLP_18845	-0,75	-1,7	1,1,E-02
LIMLP_RS18825	LIMLP_18865	0,59	1,5	2,2,E-03
LIMLP_RS18850	LIMLP_18890	-0,83	-1,8	3,8,E-03
LIMLP_RS18855	LIMLP_18895	-0,80	-1,7	5,0,E-03
LIMLP_RS18875	LIMLP_18915	0,90	1,9	4,3,E-04
LIMLP_RS18890	LIMLP_18930	0,67	1,6	4,1,E-03
LIMLP_RS18905	LIMLP_18945	0,77	1,7	2,3,E-03
LIMLP_RS18910	LIMLP_18950	1,17	2,2	1,0,E-08
LIMLP_RS18915	LIMLP_18955	0,66	1,6	4,4,E-02
LIMLP_RS18920	LIMLP_18960	0,51	1,4	1,6,E-02
LIMLP_RS18940	LIMLP_18980	0,76	1,7	1,5,E-02
LIMLP_RS18960	LIMLP_19000	1,74	3,3	8,1,E-04
LIMLP_RS18990	LIMLP_19030	-1,78	-3,4	1,2,E-04
LIMLP_RS18995	LIMLP_19035	-0,64	-1,6	1,9,E-04
LIMLP_RS19030	LIMLP_19070	0,88	1,8	3,9,E-04
LIMLP_RS19090	LIMLP_19130	-0,90	-1,9	2,0,E-02
LIMLP_RS19095	LIMLP_19135	1,05	2,1	5,1,E-05
LIMLP_RS19105	LIMLP_19145	1,27	2,4	2,3,E-04
LIMLP_RS19110	LIMLP_19150	1,34	2,5	2,8,E-13
LIMLP_RS19115	LIMLP_19155	1,09	2,1	4,6,E-05
LIMLP_RS19135	LIMLP_19175	-0,78	-1,7	4,4,E-02
LIMLP_RS19150	LIMLP_19190	2,24	4,7	5,4,E-08
LIMLP_RS19155	LIMLP_19195	2,67	6,3	1,9,E-08
LIMLP_RS19160	LIMLP_19200	1,30	2,5	5,2,E-04
LIMLP_RS19165	LIMLP_19205	1,04	2,1	3,0,E-03
LIMLP_RS19170	LIMLP_19210	0,76	1,7	2,8,E-03

LIMLP_RS19180	LIMLP_19220	-1,77	-3,4	1,1,E-04
LIMLP_RS19230	LIMLP_19270	0,56	1,5	7,4,E-03
LIMLP_RS19260	LIMLP_19300	0,56	1,5	4,8,E-02
LIMLP_RS19280	LIMLP_19320	-3,28	-9,7	4,0,E-06
LIMLP_RS19285	LIMLP_19325	-4,76	-27,1	2,0,E-06
LIMLP_RS19305	LIMLP_19345	0,51	1,4	2,3,E-02
LIMLP_RS19330	LIMLP_19370	0,64	1,6	3,0,E-02
LIMLP_RS19350	LIMLP_19390	1,17	2,2	5,5,E-04
LIMLP_RS19355	LIMLP_19395	-0,62	-1,5	3,3,E-02
LIMLP_RS19370	LIMLP_19410	0,98	2,0	3,3,E-03
LIMLP_RS19375	LIMLP_19415	1,11	2,2	1,2,E-02
LIMLP_RS19395	LIMLP_19435	-0,80	-1,7	2,0,E-05
LIMLP_RS19400	LIMLP_19440	-0,80	-1,7	3,3,E-07
LIMLP_RS22310	LIMLP_19445	-0,62	-1,5	2,1,E-03
LIMLP_RS19410	LIMLP_19450	1,17	2,3	5,3,E-04
LIMLP_RS19415	LIMLP_19455	-0,78	-1,7	3,3,E-03
LIMLP_RS19455	LIMLP_19495	-0,44	-1,4	7,9,E-03
LIMLP_RS19470	LIMLP_19510	1,10	2,1	5,5,E-04
LIMLP_RS19525	LIMLP_19565	-0,64	-1,6	2,2,E-04
LIMLP_RS19565	LIMLP_19605	1,04	2,0	1,6,E-02
LIMLP_RS19570	LIMLP_19610	3,04	8,2	5,6,E-09
LIMLP_RS19575	LIMLP_19615	1,59	3,0	2,4,E-03
LIMLP_RS19580	LIMLP_19620	0,95	1,9	9,1,E-03
LIMLP_RS19590	LIMLP_19630	0,91	1,9	6,9,E-05

Supplementary Table 2: Differentially expressed genes related to motility and chemotaxis

	Locus	Gene name	Fold Change	padj
Flagellar body and mobility	LIMLP_06785	*fliI*	2,4	8,0,E-03
	LIMLP_06790	*fliJ*	2,3	2,3,E-03
	LIMLP_07480	*flaB*	2,1	4,3,E-02
	LIMLP_08925	*fliG*	1,8	3,8,E-02
	LIMLP_06770	*fliH*	1,6	2,1,E-02
	LIMLP_06490	*flgA*	-1,5	8,2,E-04
	LIMLP_06495	*flgH*	-1,5	4,1,E-04
	LIMLP_06725	*flhG*	-1,5	2,7,E-04
	LIMLP_06710	*flhB*	-1,6	5,6,E-03
	LIMLP_05180	*flgM*	-1,6	3,3,E-02
	LIMLP_06500	*flgI*	-1,6	3,8,E-04
	LIMLP_06705	*fliR*	-1,6	3,4,E-02
	LIMLP_14615	*fliL*	-1,6	1,0,E-03
	LIMLP_06720	*flhF*	-1,7	1,0,E-05
	LIMLP_00125	*fliG*	-1,7	1,6,E-03
	LIMLP_06715	*flhA*	-1,8	5,3,E-05
	LIMLP_06485	*flgG*	-1,8	8,2,E-05
	LIMLP_16275	*flgC*	-1,8	3,8,E-06
	LIMLP_06505	*flgJ*	-1,8	1,9,E-02
	LIMLP_10160	*flgF*	-1,8	2,3,E-04
	LIMLP_17585	*flgL*	-1,8	1,3,E-03
	LIMLP_13775	*flaA-1*	-2,0	8,4,E-04
	LIMLP_09180	*fcpB*	-2,0	2,0,E-05
	LIMLP_02150		-2,0	7,6,E-04
	LIMLP_16280	*fliE*	-2,1	8,6,E-06
	LIMLP_14625	*motB*	-2,1	2,0,E-08
	LIMLP_17590	*flgK*	-2,2	1,7,E-06
	LIMLP_05750	*flgD*	-2,2	3,9,E-09
	LIMLP_14630	*motA*	-2,3	5,8,E-07
	LIMLP_02700		-2,3	1,7,E-04
	LIMLP_07475	*flaB*	-2,3	1,9,E-06
	LIMLP_13780	*flaA-2*	-2,4	2,2,E-09
	LIMLP_14620	*fliL*	-2,4	1,8,E-06
	LIMLP_14635	*flbD*	-2,4	2,1,E-11
	LIMLP_05755	*flgE*	-2,4	8,0,E-06
	LIMLP_11310	*flaA*	-2,6	1,3,E-06
	LIMLP_01630	*fcpA*	-2,8	1,3,E-08
	LIMLP_09410	*flaB-3*	-3,1	1,6,E-05
	LIMLP_09405	*flaB-2*	-3,4	4,0,E-06
Chemotaxis	LIMLP_06865		11,1	8,4,E-06
	LIMLP_11115	*cheW*	-1,6	1,8,E-03

	LIMLP_14950	*cheY-like*	-1,8	2,7,E-02
	LIMLP_17355		-1,8	3,5,E-05
	LIMLP_17325	*dmcA*	-2,0	1,3,E-03
	LIMLP_05655	*cheX*	-2,1	1,9,E-06
	LIMLP_07425	*cheD*	-2,1	4,9,E-02
	LIMLP_07440	*cheA*	-2,4	2,8,E-02
	LIMLP_07445		-2,7	2,0,E-03
	LIMLP_07450	*cheY*	-3,1	4,6,E-03

Supplementary Table 3: Differentially expressed genes related to cell division

	Locus	Gene name	Fold Change	padj
Cell Wall/ membrane/ envelope biogenesis	LIMLP_09075		2,0	9,5,E-06
	LIMLP_12790	*lnt*	1,7	1,7,E-02
	LIMLP_08630	*pbp*	1,5	3,6,E-02
	LIMLP_07520	*kdsA*	1,3	3,1,E-02
Cell cycle control, cell division, chromosome	LIMLP_07150	*ats1*	3,7	1,1,E-02
	LIMLP_02955		2,4	8,5,E-04
	LIMLP_12010	*smc*	2,1	1,1,E-02
	LIMLP_17775	*parA/ soj*	1,7	2,7,E-03
	LIMLP_17770	*parB*	1,6	4,8,E-04
Divisome	LIMLP_01155	*parA*	-1,3	1,7,E-02
	LIMLP_13115		-1,5	2,5,E-03
	LIMLP_02585	*ftsQ*	-1,6	4,8,E-03
	LIMLP_02590	*ftsA*	-1,6	2,7,E-03
	LIMLP_02595	*ftsZ*	-1,7	3,9,E-05
	LIMLP_15095	*ftsI*	-1,8	2,5,E-02
	LIMLP_05130	*ftsK*	-2,1	4,4,E-05
	LIMLP_00285	*rlpA*	-2,2	1,4,E-06
	LIMLP_09265	*ftsW*	-2,2	1,5,E-04
	LIMLP_03590	*obgE*	-2,3	1,4,E-02
Elongasome	LIMLP_15065	*mrcA / pbp1*	-1,4	4,3,E-02
	LIMLP_06170	*ftsI*	-1,6	3,5,E-02
	LIMLP_06155	*mreB*	-1,6	2,4,E-02
	LIMLP_07295		-1,3	4,1,E-03
	LIMLP_13845		-2,0	1,1,E-02
	LIMLP_11230		-2,1	1,0,E-02
	LIMLP_06160	*mreC*	-1,8	1,3,E-02
	LIMLP_06175	*rodA*	-1,9	2,5,E-03
	LIMLP_06165	*mreD*	-1,9	2,4,E-02
Peptidoglycan biosynthesis	LIMLP_05580		2,0	4,9,E-04
	LIMLP_04840		2,0	1,2,E-02
	LIMLP_13120	*nlpD*	-1,5	4,2,E-02
	LIMLP_07100	*uppP*	-1,5	3,3,E-02
	LIMLP_06825	*nlpD*	-1,5	1,3,E-02
	LIMLP_15510	*glmM*	-1,6	3,6,E-02
	LIMLP_07655	*nlpD*	-1,7	9,4,E-03
	LIMLP_04810	*lruA*	-1,8	1,1,E-04
	LIMLP_19130		-1,9	2,0,E-02
	LIMLP_15520		-1,9	2,8,E-05
	LIMLP_09270	*mraY*	-2,0	1,8,E-05
	LIMLP_17195	*nlpD*	-3,0	2,8,E-13

**LPS Bionsynthesis**	LIMLP_09065	*gmhA*	1,6	4,3,E-03
	LIMLP_10725	*kdnB*	-1,5	6,6,E-03
	LIMLP_11320	*kdtA/ waaA*	-1,5	4,3,E-02
	LIMLP_10525	*rmlC*	-1,5	3,8,E-02
	LIMLP_10685	*kdsB*	-1,6	9,8,E-03
	LIMLP_10515	*rffG/ rmlB*	-1,6	4,3,E-02
	LIMLP_17670	*lpxD2*	-1,7	1,1,E-02
	LIMLP_01675	*lpxA*	-1,7	5,7,E-04
**Membrane biogenesis**	LIMLP_12410		-2,0	3,9,E-05
	LIMLP_16615	*lnt*	-2,4	5,8,E-05
	LIMLP_05140	*lgt*	-2,6	2,8,E-03

Supplementary Table 4: Differentially expressed genes related to global cellular metabolism

	Locus	Gene name	Fold Change	padj
NADH metabolism (complex I)	LIMLP_03760	*nuoA*	-1,4	2,6,E-02
	LIMLP_03730	*nuoH*	-1,6	2,0,E-02
	LIMLP_03725	*nuoJ*	-1,8	4,7,E-03
	LIMLP_03720	*nuoK*	-2,0	4,5,E-04
	LIMLP_03705	*nuoN*	-2,1	5,6,E-06
	LIMLP_03715	*nuoL*	-2,2	4,8,E-04
	LIMLP_03710	*nuoM*	-2,5	2,3,E-04
Cytochrome C (KEGG complex IV)	LIMLP_00150		-1,3	4,9,E-02
	LIMLP_13300	*nrfD*	-1,8	4,0,E-02
	LIMLP_01100	*cyoA*	-1,8	3,4,E-02
	LIMLP_13290		-1,8	3,4,E-02
	LIMLP_01375		-1,9	2,4,E-03
	LIMLP_01105	*cyoB*	-2,3	3,3,E-03
	LIMLP_09370		-2,3	3,4,E-06
	LIMLP_00145		-2,4	2,7,E-08
	LIMLP_01110	*cyoC*	-2,4	5,0,E-04
ATP synthase	LIMLP_06070	*atpG*	-1,8	1,8,E-02
	LIMLP_06065	*atpA*	-2,0	3,3,E-02
	LIMLP_06060	*atpH*	-2,0	2,7,E-02
	LIMLP_06055	*atpF*	-2,0	1,7,E-02
	LIMLP_06045	*atpB*	-2,2	1,0,E-03
	LIMLP_06080	*atpC*	-2,3	1,8,E-03
	LIMLP_06075	*atpD*	-2,3	4,7,E-03
	LIMLP_06050	*atpE*	-2,3	3,0,E-03
Cofactor metabolism	LIMLP_14320	*petE*	-1,5	3,9,E-02
	LIMLP_19175	*phhB*	-1,7	4,4,E-02
	LIMLP_16325		-2,0	4,2,E-02
	LIMLP_00245	*pntA*	-2,1	1,5,E-02
	LIMLP_16320	*pntB*	-2,7	1,8,E-03
Methylglyoxal pathway	LIMLP_01865		6,8	8,3,E-25
	LIMLP_03805	*mgsA*	5,2	1,1,E-17
	LIMLP_08945	*gloB*	2,6	1,2,E-04
	LIMLP_17535	*kefC*	-1,6	5,7,E-03
	LIMLP_11025	*kefB*	-1,8	6,5,E-04
	LIMLP_00375		-1,8	2,3,E-03
	LIMLP_17560	*kefB*	-1,9	2,9,E-05
Carbohydrate transport	LIMLP_08890		3,7	5,6,E-14
	LIMLP_18450	*pmgA*	3,3	7,6,E-06
	LIMLP_07055	*gck*	3,2	6,1,E-11
	LIMLP_06570		1,9	5,8,E-03

	LIMLP_07550	*ptsH*	1,6	3,2,E-02
	LIMLP_14140	*glcD*	1,4	2,3,E-02
	LIMLP_03500	*coaX*	-1,4	4,8,E-02
	LIMLP_15695		-1,4	3,1,E-03
	LIMLP_16655	*adhP*	-1,5	4,2,E-03
	LIMLP_00240		-1,6	3,3,E-02
	LIMLP_06625		-1,6	5,5,E-03
	LIMLP_11505	*nagC*	-1,7	7,6,E-03
	LIMLP_10350	*tpiA*	-1,7	2,3,E-02
	LIMLP_02640		-1,8	1,3,E-04
	LIMLP_13685		-1,8	2,5,E-03
	LIMLP_17370	*acs*	-1,8	9,0,E-03
	LIMLP_08060	*tal*	-1,9	5,0,E-03
	LIMLP_09605		-1,9	5,9,E-03
	LIMLP_15480		-1,9	2,4,E-04
	LIMLP_01075		-1,9	2,1,E-03
	LIMLP_07950		-2,2	1,9,E-05
	LIMLP_04590	*adhP*	-2,4	2,0,E-15
	LIMLP_18670		-2,9	5,1,E-05
	LIMLP_14640		-3,2	1,6,E-09
	LIMLP_16375	*adhP*	-3,6	7,9,E-14
**Cycle de Krebs**	LIMLP_12355	*sucB*	-1,4	1,9,E-02
	LIMLP_02815	*gltA*	-1,7	7,9,E-03
	LIMLP_00845	*fumC*	-2,0	6,4,E-07
	LIMLP_01230	*icd*	-2,0	1,4,E-02
	LIMLP_12885	*sucC*	-2,1	4,9,E-03
	LIMLP_11605	*prpB*	-2,3	5,4,E-06
**Lipid transport and metabolism**	LIMLP_11535	*sch/ fabF*	6,2	1,1,E-06
	LIMLP_08580	*fadH*	2,3	1,3,E-03
	LIMLP_17400		2,3	5,0,E-05
	LIMLP_16885		1,9	1,9,E-02
	LIMLP_05560		1,9	3,3,E-02
	LIMLP_13460	*acpS*	1,4	3,1,E-02
	LIMLP_05340	*icmF*	-1,5	2,8,E-02
	LIMLP_09620		-1,6	2,1,E-04
	LIMLP_08930	*glpK*	-1,6	1,6,E-05
	LIMLP_16515	*ccr*	-1,8	2,9,E-02
	LIMLP_14735	*pldB*	-1,8	4,1,E-02
	LIMLP_16035		-1,8	2,4,E-03
	LIMLP_12830		-1,8	1,2,E-02
	LIMLP_06675	*psd*	-2,0	1,2,E-03
	LIMLP_15525		-2,4	7,8,E-10
	LIMLP_14590	*glpK*	-3,1	1,6,E-14
	LIMLP_11000		-3,2	2,9,E-03
**Fat ty**	LIMLP_00095	*fabG*	-1,5	4,1,E-03

	LIMLP_18160	*acpP*	-1,6	1,9,E-02
	LIMLP_07405		-1,8	3,1,E-02
	LIMLP_16970	*fas1*	-2,4	2,9,E-04
	LIMLP_14230	*fabG*	-2,7	1,1,E-09
**Beta oxydation**	LIMLP_09860	*caiA*	1,4	1,7,E-02
	LIMLP_03845	*caiD*	-1,5	3,8,E-04
	LIMLP_04665		-1,7	1,1,E-02
	LIMLP_12760		-1,8	8,1,E-04
	LIMLP_08150	*caiD*	-1,9	5,4,E-03
	LIMLP_15025	*caiA*	-2,0	3,4,E-04
	LIMLP_15860		-2,2	5,9,E-05
	LIMLP_13700	*caiA*	-2,3	3,1,E-05
**Ribosomal structure and biogenesis**	LIMLP_13505	*rimL*	2,3	3,4,E-04
	LIMLP_02720	*rpsA*	2,1	6,5,E-08
	LIMLP_11740	*bipA*	2,1	4,6,E-02
	LIMLP_02725	*cspR*	2,1	2,2,E-09
	LIMLP_07790	*pth*	1,8	4,1,E-04
	LIMLP_16085	*mtaB*	1,4	1,2,E-02
	LIMLP_03205	*rpsK*	-1,5	3,8,E-02
	LIMLP_13895	*rpsI*	-1,6	1,0,E-02
	LIMLP_13900	*rplM*	-1,6	3,8,E-02
	LIMLP_13440	*rpsB*	-1,7	2,4,E-02
	LIMLP_13955	*rplU*	-1,7	1,6,E-03
	LIMLP_03115	*rplP*	-1,7	3,3,E-02
	LIMLP_13930	*rpsG*	-1,7	2,2,E-02
	LIMLP_03140	*rplE*	-1,8	3,0,E-02
	LIMLP_03155	*rplF*	-1,8	1,8,E-02
	LIMLP_03220	*rplQ*	-1,8	5,6,E-03
	LIMLP_03135	*rplX*	-1,8	2,4,E-02
	LIMLP_15595	*rplY*	-1,9	5,9,E-04
	LIMLP_03110	*rpsC*	-1,9	1,4,E-02
	LIMLP_03100	*rpsS*	-1,9	2,4,E-02
	LIMLP_03095	*rplB*	-1,9	2,6,E-02
	LIMLP_03210	*rpsD*	-1,9	3,6,E-03
	LIMLP_03175	*rplO*	-2,0	4,2,E-03
	LIMLP_13950	*rplL*	-2,0	2,5,E-05
	LIMLP_03585	*rpmA*	-2,3	2,2,E-05
**Translation**	LIMLP_00100	*sua*	2,9	6,4,E-09
	LIMLP_08730	*prfH*	1,5	1,5,E-02
	LIMLP_10110	*asnS*	-1,4	1,5,E-02
	LIMLP_01160	*gatB*	-1,5	4,0,E-02
	LIMLP_12290	*thrS*	-1,6	9,5,E-03
	LIMLP_16365	*yqxC*	-1,6	1,0,E-02
	LIMLP_13435	*tsf*	-1,7	3,1,E-02
	LIMLP_07120	*gatA*	-1,8	6,5,E-03

	LIMLP_15590	*pth*	-1,8	2,4,E-02
	LIMLP_08535	*sua5*	-1,9	2,2,E-02
**Biosynthesis**	LIMLP_09645	*pcnB*	2,4	8,7,E-05
	LIMLP_07370	*def*	1,9	5,9,E-04
	LIMLP_11910	*gatA*	1,8	1,2,E-02
**Amino acid metabolism**	LIMLP_00290	*cysE*	3,5	7,5,E-08
	LIMLP_02360	*hisI*	2,4	4,5,E-06
	LIMLP_07785	*cimA*	2,3	3,4,E-05
	LIMLP_10260	*panD*	2,3	3,4,E-05
	LIMLP_14155		2,2	1,1,E-02
	LIMLP_08570	*leuA*	2,2	5,7,E-04
	LIMLP_00400	*dapF*	2,1	4,5,E-08
	LIMLP_08880		1,9	3,7,E-03
	LIMLP_06420		1,8	4,5,E-02
	LIMLP_11565	*atoAD*	1,8	2,7,E-02
	LIMLP_16210	*gcvT*	1,7	1,1,E-03
	LIMLP_08670	*argC*	1,6	8,7,E-04
	LIMLP_05335		1,5	3,4,E-02
	LIMLP_13480	*dapA*	1,5	8,4,E-03
	LIMLP_06645	*prlC*	1,5	1,9,E-02
	LIMLP_13475	*dapB*	1,5	3,1,E-03
	LIMLP_00600	*hisH*	-1,3	4,5,E-02
	LIMLP_00320	*gltP*	-1,3	3,4,E-02
	LIMLP_00605	*hisA*	-1,4	3,5,E-02
	LIMLP_08775	*leuB*	-1,4	1,7,E-02
	LIMLP_08770	*argD*	-1,5	4,9,E-02
	LIMLP_16190	*serC*	-1,5	9,8,E-03
	LIMLP_18295	*argB*	-1,6	3,6,E-03
	LIMLP_19035	*ablA*	-1,6	1,9,E-04
	LIMLP_03600	*proA*	-1,6	2,7,E-02
	LIMLP_08865	*putP*	-1,7	1,0,E-02
	LIMLP_07125	*hisF*	-1,7	2,4,E-02
	LIMLP_15515	*glmS*	-1,7	5,2,E-04
	LIMLP_18175	*aroB*	-1,8	3,5,E-05
	LIMLP_16730	*argJ*	-1,9	1,3,E-05
	LIMLP_11985	*glnA*	-2,0	1,6,E-02
	LIMLP_08755		-2,2	9,5,E-07

Supplementary Table 5: Differentially expressed genes encoding enzymes involved in c-di-GMP metabolism

	Locus	Fold Change	padj
**HD-GYP domain**	LIMLP_07630	2,6	6,9,E-05
	LIMLP_00730	1,5	1,2,E-02
	LIMLP_00735	-2,5	4,5,E-03
	LIMLP_05760	Not significantly modulated/ NS	
**GGDEF domain**	LIMLP_18785	1,5	2,0,E-02
	LIMLP_18780	NS	
	LIMLP_05485	NS	
	LIMLP_01765	NS	
	LIMLP_11285	NS	
	LIMLP_07050	NS	
	LIMLP_06380	NS	
	LIMLP_05475	NS	
	LIMLP_05470	-1,6	4,2,E-03
	LIMLP_05465	-1,7	7,2,E-06
	LIMLP_05460	-2,0	9,7,E-04
	LIMLP_05455	-2,7	1,7,E-06
	LIMLP_05450	-3,2	4,2,E-07
**EAL domain**	LIMLP_09580	2,6	1,1,E-04
	LIMLP_05845	NS	
	LIMLP_18870	NS	
	LIMLP_18375	NS	
	LIMLP_04775	-1,6	3,7,E-02
**Hybrids GGDEF/EAL**	LIMLP_04405	NS	
	LIMLP_18775	NS	
	LIMLP_01855	NS	
**Effectors** (identified by Vasconcelos *et al.*, 2023)	LIMLP_12520	1,8	3,0,E-03
	LIMLP_06735	NS	
	LIMLP_08965	NS	
	LIMLP_17575	NS	
	LIMLP_11140	NS	
	LIMLP_03470	NS	
	LIMLP_01855	NS	
	LIMLP_11285	NS	
	LIMLP_06380	NS	
	LIMLP_14530	NS	
	LIMLP_03970	NS	
	LIMLP_07670	NS	
	LIMLP_12435	NS	
	LIMLP_09575	NS	
	LIMLP_02460	NS	
	LIMLP_06095	-1,8	2,2,E-03

	LIMLP_03855	-1,9	1,8,E-03

Supplementary Table 6: Differentially expressed genes related to the general stress response

	Locus	*Gene name*	Fold Change	padj
**Chaperone**	LIMLP_10060	*clpB*	21,4	1,3,E-22
	LIMLP_10970	*ibpA*	11,3	3,5,E-04
	LIMLP_10975	*ibpB?/hsp*	9,6	5,8,E-04
	LIMLP_15115	*dnaK*	3,9	3,6,E-04
	LIMLP_15120	*dnaJ*	3,7	8,0,E-04
	LIMLP_12325		3,7	3,7,E-10
	LIMLP_15110	*grpE*	3,6	9,1,E-06
	LIMLP_16505		3,5	2,8,E-13
	LIMLP_15105	*hrcA*	3,5	7,0,E-08
	LIMLP_02755		-1,7	1,8,E-04
**Protease (clp, lon)**	LIMLP_09010	*clpS*	3,6	1,3,E-06
	LIMLP_09005	*clpA*	2,8	1,2,E-06
	LIMLP_03390	*degQ/ mucD*	2,8	1,2,E-09
	LIMLP_07500		2,8	1,7,E-07
	LIMLP_06915	*clpP*	2,2	2,9,E-04
	LIMLP_18560	*htpX*	1,8	1.996e-8
	LIMLP_06920	*clpX*	1,7	2,4,E-09
	LIMLP_14705	*lon*	1,7	1,5,E-05
**DNA Repair SOS response**	LIMLP_11400	*radC*	4,6	9,4,E-05
	LIMLP_03395	*ruvB*	3,1	1,0,E-09
	LIMLP_07750	*xseB*	2,8	6,8,E-03
	LIMLP_06425		2,5	1,2,E-02
	LIMLP_07915	*recN*	2,3	4,5,E-02
	LIMLP_11765	*alkA*	1,9	5,6,E-07
	LIMLP_08725	*uvrC*	1,9	1,0,E-04
	LIMLP_08665	*recA*	1,8	2,4,E-02
	LIMLP_12570	*mutL*	1,8	9,7,E-05
	LIMLP_16525	*rad50*	1,8	2,5,E-02
	LIMLP_05535	*radA*	1,8	2,0,E-03
	LIMLP_07755	*xseA*	1,7	3,5,E-05
	LIMLP_17705	*recR*	1,7	2,7,E-02
	LIMLP_09175	*spoVK*	1,7	3,7,E-03
	LIMLP_08800	*mutS*	1,7	7,0,E-04
	LIMLP_06550	*phr*	1,4	3,2,E-03
	LIMLP_05155	*recJ*	1,3	4,8,E-02
	LIMLP_14660	*dprA*	-1,5	1,7,E-02
	LIMLP_04440	*hsdR*	-1,5	1,8,E-02
	LIMLP_12765		-1,6	2,3,E-03
	LIMLP_12955	*uvrD*	-1,6	5,2,E-03
	LIMLP_01930		-1,6	2,4,E-04

**Replication**	LIMLP_04105	*cdc9*	5,9	3,4,E-10
	LIMLP_08885	*ssl*	2,2	1,6,E-03
	LIMLP_08300		2,1	5,3,E-03
	LIMLP_10290	*topA*	1,9	6,2,E-03
	LIMLP_01345	*hepA*	1,7	7,9,E-03
	LIMLP_07570	*priA*	1,6	1,2,E-02
	LIMLP_07935	*pcrA*	1,6	1,2,E-03
	LIMLP_00025	*gyrB*	1,4	1,3,E-02
	LIMLP_00010	*dnaN*	1,4	3,3,E-02
	LIMLP_00030	*gyrA*	1,3	1,1,E-02
	LIMLP_03780		-1,6	1,8,E-02
	LIMLP_04495		-1,9	3,5,E-06
	LIMLP_06480		-3,3	2,0,E-12
**Recombination**	LIMLP_15170		1,9	1,0,E-02
	LIMLP_07730		1,7	2,7,E-05
	LIMLP_13615		1,7	1,3,E-04
	LIMLP_05875		1,7	2,3,E-05
	LIMLP_09845		1,7	1,9,E-04
	LIMLP_07810		1,7	6,5,E-04
	LIMLP_12550	*rarA*	1,6	5,2,E-03
	LIMLP_08920		1,6	8,6,E-05
	LIMLP_02990		1,6	3,5,E-04
	LIMLP_09325		1,6	6,8,E-04
	LIMLP_18750		1,6	7,3,E-04
	LIMLP_04300		1,6	2,6,E-03
	LIMLP_18610		1,5	4,1,E-03
	LIMLP_01995		1,5	1,2,E-03
	LIMLP_18385		1,5	3,3,E-03
	LIMLP_05775		1,4	1,5,E-02
**Copper efflux operon**	LIMLP_02515	*csoR*	29,6	4,1,E-09
	LIMLP_02520	*copZ*	28,4	9,1,E-09
	LIMLP_02525	*copA*	11,5	1,1,E-16
	LIMLP_14415	*arsR*	2,4	2,1,E-02
**Defense mechanisms**	LIMLP_02080	*acrR*	3,0	2,0,E-03
	LIMLP_01780	*acrB*	3,2	3,9,E-19
	LIMLP_01775	*tolC*	2,3	4,9,E-04
	LIMLP_00720	*mdlb*	2,1	8,1,E-03
	LIMLP_10285	*modF*	1,9	5,0,E-03
	LIMLP_02355	*acrB-like*	1,6	9,3,E-03
	LIMLP_07365		1,5	2,9,E-02
	LIMLP_08515	*czcD*	1,5	1,3,E-02
	LIMLP_09060	*dppD*	1,4	6,3,E-03
**Redox Homeost asis**	LIMLP_16870		5,9	6,2,E-22
	LIMLP_11965	*dsbD*	4,5	2,0,E-15
	LIMLP_04015	*dsbH*	3,1	8,0,E-16

	LIMLP_07145		2,7	8,1,E-04
	LIMLP_09870	*trxA*	2,5	5,5,E-08
	LIMLP_18140		2,5	5,1,E-10
	LIMLP_03055		2,4	1,4,E-03
	LIMLP_02615	*nadA*	2,3	1,6,E-03
	LIMLP_11770	*tlpA-like*	2,3	1,1,E-05
	LIMLP_06190		2,1	4,7,E-03
	LIMLP_07165	*trxB*	2,0	4,5,E-03
	LIMLP_12405		1,9	1,2,E-02
**Cytochrome C**	LIMLP_04655	*ccmG*	3,1	3,1,E-05
	LIMLP_13650	*ccmI*	4,7	6,4,E-06
	LIMLP_13635	*ccmE*	3,8	2,5,E-11
	LIMLP_13640	*ccmF*	2,6	1,6,E-05
	LIMLP_13645	*ccmH*	2,3	1,5,E-03
**Glutathion metabolism**	LIMLP_13670	*yfcG*	10,9	4,3,E-26
	LIMLP_08975	*bolA*	3,1	2,6,E-02
	LIMLP_08995	*gshA*	2,6	3,4,E-14
	LIMLP_08990	*gshB*	2,6	2,6,E-13
	LIMLP_08985		2,6	8,2,E-08
	LIMLP_08980		2,5	1,2,E-03
	LIMLP_09000	*ggt*	1,6	1,5,E-02
**Sulfur metabolism**	LIMLP_00290	*cysE*	3,5	7,5,E-08
	LIMLP_17230	*cysH*	2,2	2,1,E-03
	LIMLP_05710		2,1	3,8,E-02
	LIMLP_17220	*cysN*	2,0	3,5,E-03
	LIMLP_17225	*cysD*	2,0	2,3,E-03
**Fe-S Cluster assembly**	LIMLP_14560	*sufU*	4,8	1,5,E-06
	LIMLP_14555	*sufT*	3,7	2,8,E-09
	LIMLP_14565	*sufS*	2,3	4,1,E-06
	LIMLP_13200	*nifU*	2,2	6,1,E-04
	LIMLP_14570	*bedB*	2,2	3,7,E-04
	LIMLP_05960	*sufB*	1,7	4,9,E-04
	LIMLP_14575	*sufD*	1,7	4,6,E-02
	LIMLP_08130	*nifS*	1,7	7,5,E-03
	LIMLP_14580	*sufC*	1,6	6,9,E-04
**Fe uptake**	LIMLP_04310	*fecR*	8,3	3,8,E-30
	LIMLP_06430	*bfr*	2,7	3,3,E-02
	LIMLP_06840	*feoB*	1,8	9,7,E-04
	LIMLP_04270	*fecA*	-5,1	2,2,E-02
**Fe-S cluster target**	LIMLP_07845	*mrp*	4,1	1,2,E-09
	LIMLP_17335		2,8	2,8,E-04
	LIMLP_06405		2,0	1,6,E-04
	LIMLP_09120	*fer*	1,8	6,2,E-03
	LIMLP_04595		-2,3	3,2,E-02

Supplementary Table 7: Comparison of the differentially expressed genes upon H2O2 treatment for 1h. Green highlighting indicates a different regulation pattern.

Locus	WT with 1 mM H2O2 vs WT Log_2FC	WT with 1 mM H2O2 vs WT padj	WT biofilm vs planktonic Log2FC	WT biofilm vs planktonic padj
LIMLP_00020	1,00	0,00	2,29	0,00
LIMLP_00130	-1,80	0,00		
LIMLP_00140	-1,08	0,00		
LIMLP_00175	-1,30	0,00		
LIMLP_00180	-1,16	0,00		
LIMLP_00185	-1,22	0,00		
LIMLP_00330	-1,04	0,00		
LIMLP_00340	-1,14	0,00		
LIMLP_00370	1,26	0,00	2,00	0,00
LIMLP_00430	3,06	0,00		
LIMLP_00440	1,27	0,00		
LIMLP_00455	1,11	0,00		
LIMLP_00460	1,61	0,00		
LIMLP_00510	-2,31	0,00		
LIMLP_00615	-1,07	0,00	-1,11	0,02
LIMLP_00700	1,69	0,00		
LIMLP_00770	3,79	0,00	1,73	0,00
LIMLP_00795	1,37	0,00		
LIMLP_00810	-1,49	0,00		
LIMLP_00815	-1,36	0,00		
LIMLP_00820	-2,41	0,00	-1,15	0,00
LIMLP_00825	-2,20	0,00	-1,44	0,00
LIMLP_00830	-1,54	0,00		
LIMLP_00895	3,66	0,00	1,30	0,03
LIMLP_01025	1,03	0,00	1,64	0,00
LIMLP_01045	1,22	0,00	1,40	0,00
LIMLP_01055	-1,01	0,00		
LIMLP_01115	-1,90	0,03		
LIMLP_01200	1,55	0,00		
LIMLP_01260	1,00	0,00		
LIMLP_01280	2,41	0,00		
LIMLP_01370	-2,36	0,04		
LIMLP_01410	-1,09	0,00		
LIMLP_01455	-1,44	0,00	-1,38	0,00
LIMLP_01480	-1,40	0,00	-1,85	0,00
LIMLP_01540	-1,43	0,00		
LIMLP_01545	2,44	0,00		
LIMLP_01550	1,40	0,00		
LIMLP_01665	-1,00	0,00		

LIMLP_01735	-1,31	0,00		
LIMLP_01740	-1,45	0,00		
LIMLP_01810	1,08	0,00		
LIMLP_01845	1,11	0,00		
LIMLP_01865	1,93	0,00	2,77	0,00
LIMLP_01965	-1,67	0,00		
LIMLP_02010	-3,31	0,00		
LIMLP_02040	1,49	0,00	1,85	0,00
LIMLP_02045	1,34	0,00	2,30	0,00
LIMLP_02105	1,31	0,00	1,37	0,02
LIMLP_02170	2,33	0,00		
LIMLP_02225	-1,74	0,00	-2,50	0,00
LIMLP_02290	-1,27	0,03		
LIMLP_02395	-1,04	0,00		
LIMLP_02400	-1,04	0,00	-2,39	0,00
LIMLP_02420	1,50	0,00		
LIMLP_02470	-1,18	0,00		
LIMLP_02515	2,62	0,00	4,89	0,00
LIMLP_02520	2,06	0,00	4,83	0,00
LIMLP_02525	1,94	0,00	3,52	0,00
LIMLP_02605	-1,00	0,00		
LIMLP_02635	1,38	0,00		
LIMLP_02670	-1,07	0,00		
LIMLP_02675	-1,15	0,00		
LIMLP_02740	-1,04	0,00		
LIMLP_02795	5,82	0,00		
LIMLP_02820	-1,52	0,00		
LIMLP_02870	-1,11	0,00		
LIMLP_02875	-1,29	0,00		
LIMLP_02880	-1,58	0,00		
LIMLP_02885	-1,21	0,00		
LIMLP_02915	-1,40	0,04		
LIMLP_02930	-1,30	0,00		
LIMLP_03095	-1,03	0,00		
LIMLP_03100	-1,07	0,00		
LIMLP_03105	-1,26	0,00		
LIMLP_03110	-1,18	0,00		
LIMLP_03115	-1,20	0,00		
LIMLP_03120	-1,31	0,00		
LIMLP_03125	-1,39	0,00		
LIMLP_03130	-1,40	0,00		
LIMLP_03135	-1,32	0,00		
LIMLP_03140	-1,35	0,00		
LIMLP_03150	-1,47	0,00		
LIMLP_03155	-1,50	0,00		
LIMLP_03160	-1,53	0,00		

LIMLP_03165	-1,56	0,00		
LIMLP_03170	-1,42	0,00		
LIMLP_03175	-1,32	0,00		
LIMLP_03180	-1,29	0,00		
LIMLP_03185	-1,00	0,00		
LIMLP_03190	-1,12	0,00		
LIMLP_03195	-1,19	0,00		
LIMLP_03200	-1,36	0,00		
LIMLP_03205	-1,20	0,00		
LIMLP_03210	-1,20	0,00		
LIMLP_03215	-1,74	0,00		
LIMLP_03220	-1,69	0,00		
LIMLP_03245	1,37	0,00		
LIMLP_03290	-1,43	0,00		
LIMLP_03295	-1,05	0,00		
LIMLP_03360	1,94	0,00	2,00	0,00
LIMLP_03365	1,48	0,00		
LIMLP_03390	1,41	0,00	1,49	0,00
LIMLP_03590	-1,07	0,00	-1,19	0,01
LIMLP_03705	-1,39	0,00	-1,09	0,00
LIMLP_03710	-1,48	0,00	-1,34	0,00
LIMLP_03715	-1,16	0,00	-1,11	0,00
LIMLP_03805	1,06	0,00	2,39	0,00
LIMLP_03975	-1,02	0,00		
LIMLP_03990	1,09	0,02		
LIMLP_04095	-1,24	0,00	1,90	0,00
LIMLP_04100	-1,04	0,00	2,40	0,00
LIMLP_04120	-1,47	0,00		
LIMLP_04175	-1,07	0,00		
LIMLP_04180	-1,41	0,00		
LIMLP_04200	-1,06	0,00		
LIMLP_04210	-1,10	0,00		
LIMLP_04220	-1,41	0,00	-1,13	0,00
LIMLP_04225	-1,17	0,00	-1,12	0,00
LIMLP_04230	-1,22	0,00		
LIMLP_04265	-1,53	0,04	-2,95	0,04
LIMLP_04295	1,02	0,00		
LIMLP_04310	1,59	0,00	3,05	0,00
LIMLP_04325	-1,05	0,00		
LIMLP_04335	-1,17	0,00		
LIMLP_04345	1,34	0,00		
LIMLP_04475	1,28	0,00		
LIMLP_04480	1,88	0,00	3,06	0,00
LIMLP_04485	1,21	0,00		
LIMLP_04555	2,40	0,00	2,79	0,00
LIMLP_04610	-1,37	0,00		

LIMLP_04615	-1,64	0,00		
LIMLP_04635	1,30	0,00		
LIMLP_04660	-2,66	0,00		
LIMLP_04670	-1,05	0,00		
LIMLP_04685	1,21	0,00		
LIMLP_04735	-1,10	0,00	1,04	0,00
LIMLP_04765	-1,25	0,00		
LIMLP_04775	1,90	0,00		
LIMLP_04805	-1,16	0,02		
LIMLP_04870	1,13	0,00		
LIMLP_04895	-1,15	0,03		
LIMLP_04935	-2,71	0,00		
LIMLP_04940	-1,29	0,00		
LIMLP_05015	1,26	0,00		
LIMLP_05020	-2,45	0,00		
LIMLP_05035	-1,25	0,00	1,01	0,01
LIMLP_05055	1,12	0,00		
LIMLP_05110	3,45	0,00	1,44	0,00
LIMLP_05115	6,38	0,00	2,03	0,02
LIMLP_05120	6,19	0,00	2,18	0,02
LIMLP_05125	1,12	0,00		
LIMLP_05135	1,22	0,00	2,62	0,00
LIMLP_05215	1,56	0,00	2,32	0,00
LIMLP_05225	-1,01	0,00		
LIMLP_05250	-1,68	0,00		
LIMLP_05255	-2,45	0,00		
LIMLP_05260	-2,30	0,00		
LIMLP_05265	-2,10	0,00		
LIMLP_05275	1,16	0,00		
LIMLP_05450	-2,16	0,00	-1,67	0,00
LIMLP_05455	-1,42	0,00	-1,43	0,00
LIMLP_05555	3,90	0,00		
LIMLP_05560	4,09	0,00		
LIMLP_05565	2,39	0,00		
LIMLP_05620	2,21	0,00	2,00	0,00
LIMLP_05860	1,32	0,00		
LIMLP_05955	4,01	0,00		
LIMLP_05960	2,23	0,00		
LIMLP_06050	-1,08	0,00	-1,23	0,00
LIMLP_06055	-1,07	0,00	-1,01	0,02
LIMLP_06060	-1,28	0,00		
LIMLP_06065	-1,44	0,00		
LIMLP_06070	-1,59	0,00		
LIMLP_06075	-1,67	0,00	-1,19	0,00
LIMLP_06080	-1,43	0,00	-1,18	0,00
LIMLP_06115	-1,07	0,00		

LIMLP_06165	-1,15	0,00		
LIMLP_06170	-1,23	0,00		
LIMLP_06175	-1,16	0,00		
LIMLP_06205	1,47	0,00		
LIMLP_06295	1,02	0,00		
LIMLP_06315	1,09	0,00		
LIMLP_06320	1,35	0,00		
LIMLP_06425	-1,63	0,00	1,31	0,01
LIMLP_06430	-1,27	0,00	1,42	0,03
LIMLP_06480	-1,39	0,00	-1,71	0,00
LIMLP_06500	-1,27	0,00		
LIMLP_06540	3,36	0,00		
LIMLP_06545	3,33	0,00		
LIMLP_06555	1,10	0,00		
LIMLP_06580	1,04	0,00		
LIMLP_06605	1,09	0,00		
LIMLP_06630	1,11	0,00	1,11	0,00
LIMLP_06705	-1,08	0,00		
LIMLP_06710	-1,18	0,00		
LIMLP_06715	-1,29	0,00		
LIMLP_06805	-1,23	0,00		
LIMLP_06825	-1,70	0,00		
LIMLP_06830	-1,57	0,00		
LIMLP_06835	-1,55	0,00		
LIMLP_06855	-1,18	0,00		
LIMLP_06870	1,01	0,00		
LIMLP_06930	-1,05	0,00		
LIMLP_07045	1,29	0,00	2,46	0,00
LIMLP_07065	1,05	0,00		
LIMLP_07070	1,51	0,00	1,31	0,01
LIMLP_07105	-1,33	0,00		
LIMLP_07110	-1,67	0,00	-1,12	0,00
LIMLP_07145	2,24	0,00	1,41	0,00
LIMLP_07150	4,96	0,00	1,90	0,01
LIMLP_07165	1,91	0,00	1,01	0,00
LIMLP_07190	-1,27	0,00		
LIMLP_07265	-1,31	0,00		
LIMLP_07335	-1,03	0,00		
LIMLP_07360	2,35	0,00		
LIMLP_07365	-1,05	0,00		
LIMLP_07380	1,23	0,00		
LIMLP_07420	-1,71	0,00		
LIMLP_07425	-1,11	0,00	-1,08	0,05
LIMLP_07600	-1,26	0,00		
LIMLP_07625	1,02	0,00	1,43	0,00
LIMLP_07780	1,02	0,00		

LIMLP_07915	5,03	0,00	1,20	0,04
LIMLP_07920	2,08	0,00		
LIMLP_07925	1,17	0,00		
LIMLP_07950	-1,12	0,00	-1,14	0,00
LIMLP_07965	-1,89	0,00		
LIMLP_07970	-1,33	0,00	-2,17	0,00
LIMLP_08080	-1,03	0,00		
LIMLP_08410	2,62	0,00		
LIMLP_08415	2,24	0,00		
LIMLP_08420	1,43	0,00		
LIMLP_08465	-1,13	0,00	-1,23	0,00
LIMLP_08590	1,29	0,00		
LIMLP_08665	2,65	0,00		
LIMLP_08685	1,07	0,00		
LIMLP_08870	-1,42	0,00	1,04	0,01
LIMLP_08930	-1,40	0,00		
LIMLP_08985	1,23	0,00	1,36	0,00
LIMLP_09015	1,18	0,00	1,92	0,00
LIMLP_09085	1,13	0,00		
LIMLP_09095	1,03	0,01	1,64	0,00
LIMLP_09190	-1,17	0,00		
LIMLP_09200	1,38	0,00		
LIMLP_09240	-1,51	0,00		
LIMLP_09265	-1,27	0,00	-1,16	0,00
LIMLP_09320	1,09	0,00		
LIMLP_09360	-1,09	0,00		
LIMLP_09365	-1,46	0,02		
LIMLP_09375	-1,26	0,00		
LIMLP_09415	1,18	0,03		
LIMLP_09595	-1,27	0,00		
LIMLP_09605	-1,01	0,00		
LIMLP_09630	-1,22	0,03		
LIMLP_09650	5,15	0,00		
LIMLP_09655	-1,17	0,01		
LIMLP_09770	-1,23	0,00		
LIMLP_09780	1,21	0,00		
LIMLP_09810	-1,57	0,00		
LIMLP_09815	1,54	0,00		
LIMLP_09940	1,52	0,00		
LIMLP_09960	-1,16	0,00		
LIMLP_09995	1,44	0,00	1,98	0,00
LIMLP_10015	1,08	0,00		
LIMLP_10020	-1,42	0,00		
LIMLP_10050	-1,13	0,00		
LIMLP_10060	2,11	0,00	4,42	0,00
LIMLP_10075	-1,03	0,00		

LIMLP_10145	2,76	0,00		
LIMLP_10150	2,70	0,00	1,65	0,00
LIMLP_10155	3,57	0,00		
LIMLP_10235	-1,39	0,00	-1,22	0,01
LIMLP_10270	1,21	0,00		
LIMLP_10275	5,47	0,00	1,64	0,01
LIMLP_10370	-1,07	0,00		
LIMLP_10405	-1,01	0,00		
LIMLP_10870	1,09	0,00		
LIMLP_10875	1,08	0,00		
LIMLP_10880	1,26	0,00		
LIMLP_10885	1,44	0,00		
LIMLP_10890	1,46	0,00		
LIMLP_10895	1,77	0,00		
LIMLP_10900	1,94	0,00		
LIMLP_10905	1,93	0,00		
LIMLP_10910	2,24	0,00		
LIMLP_10915	2,34	0,00		
LIMLP_10920	2,72	0,00		
LIMLP_10925	2,62	0,00		
LIMLP_10930	2,60	0,00		
LIMLP_10935	2,54	0,00		
LIMLP_10940	2,45	0,00		
LIMLP_10945	2,30	0,00		
LIMLP_10965	2,40	0,00		
LIMLP_10970	6,79	0,00	3,49	0,00
LIMLP_10975	6,59	0,00	3,27	0,00
LIMLP_10985	-1,04	0,00		
LIMLP_10990	-1,47	0,00		
LIMLP_10995	-1,70	0,00	-1,13	0,02
LIMLP_11000	-1,45	0,00	-1,66	0,00
LIMLP_11005	-1,33	0,00	-1,49	0,00
LIMLP_11035	1,03	0,00		
LIMLP_11130	-1,28	0,00		
LIMLP_11170	-1,25	0,00		
LIMLP_11175	-1,40	0,00		
LIMLP_11180	-1,57	0,00		
LIMLP_11185	-1,74	0,00		
LIMLP_11190	1,13	0,03		
LIMLP_11230	-1,61	0,00	-1,09	0,01
LIMLP_11395	1,62	0,00		
LIMLP_11400	3,46	0,00	2,22	0,00
LIMLP_11405	3,02	0,00	2,24	0,00
LIMLP_11410	1,32	0,00		
LIMLP_11435	1,68	0,00		
LIMLP_11440	2,40	0,00		

LIMLP_11445	1,19	0,04		
LIMLP_11485	1,56	0,00		
LIMLP_11675	-1,36	0,00		
LIMLP_11685	-1,97	0,00		
LIMLP_11715	1,05	0,00		
LIMLP_11780	-1,72	0,00		
LIMLP_11785	-1,20	0,00		
LIMLP_11840	-1,02	0,00	-1,94	0,00
LIMLP_11965	1,47	0,00	2,18	0,00
LIMLP_11980	1,36	0,00		
LIMLP_11985	1,43	0,00		
LIMLP_11995	1,07	0,00		
LIMLP_12005	1,05	0,00	1,90	0,00
LIMLP_12045	-1,32	0,00		
LIMLP_12055	1,13	0,03		
LIMLP_12130	-1,68	0,03		
LIMLP_12205	-1,72	0,00		
LIMLP_12210	-2,23	0,00		
LIMLP_12215	-1,47	0,00		
LIMLP_12400	1,10	0,00		
LIMLP_12425	1,58	0,00	2,22	0,00
LIMLP_12430	1,11	0,00	1,28	0,00
LIMLP_12480	1,02	0,00	1,34	0,00
LIMLP_12510	4,52	0,00	-2,24	0,04
LIMLP_12515	1,58	0,00		
LIMLP_12520	1,28	0,00		
LIMLP_12545	1,56	0,00	1,36	0,00
LIMLP_12590	-1,35	0,00		
LIMLP_12640	1,14	0,00		
LIMLP_12645	1,67	0,00		
LIMLP_12670	1,48	0,00	1,53	0,00
LIMLP_12680	-1,11	0,00		
LIMLP_12685	-1,39	0,00	-1,07	0,02
LIMLP_12690	-1,13	0,00		
LIMLP_12750	1,42	0,00		
LIMLP_12770	-1,28	0,00		
LIMLP_12785	2,61	0,00		
LIMLP_12790	2,57	0,00		
LIMLP_12795	1,96	0,00		
LIMLP_12905	-1,49	0,00		
LIMLP_12910	-1,79	0,00		
LIMLP_12920	1,50	0,00		
LIMLP_13010	1,15	0,00	1,64	0,01
LIMLP_13015	1,23	0,00	1,70	0,02
LIMLP_13020	1,32	0,00	1,66	0,01
LIMLP_13025	1,02	0,00	1,68	0,01

LIMLP_13145	3,87	0,00		
LIMLP_13190	-1,25	0,00		
LIMLP_13240	-1,13	0,00		
LIMLP_13490	-1,32	0,00		
LIMLP_13665	1,33	0,00	1,04	0,00
LIMLP_13670	1,76	0,00	3,44	0,00
LIMLP_13715	-1,16	0,00	-1,35	0,00
LIMLP_13720	-1,44	0,00	-1,96	0,00
LIMLP_13725	-1,39	0,00	-2,16	0,00
LIMLP_13730	-1,16	0,00	-2,19	0,00
LIMLP_13740	1,21	0,00		
LIMLP_13750	-1,01	0,00		
LIMLP_13765	3,89	0,00	1,01	0,05
LIMLP_13830	-1,25	0,00	-1,30	0,00
LIMLP_13835	-1,39	0,01		
LIMLP_13880	1,26	0,00	2,00	0,00
LIMLP_13925	1,51	0,00		
LIMLP_13930	-1,00	0,00		
LIMLP_13985	1,06	0,00		
LIMLP_14070	1,62	0,04		
LIMLP_14160	2,18	0,00		
LIMLP_14170	2,82	0,00		
LIMLP_14175	1,80	0,00		
LIMLP_14180	1,41	0,00		
LIMLP_14190	-2,13	0,00		
LIMLP_14195	-1,70	0,00	-1,09	0,00
LIMLP_14200	-1,11	0,00	-1,38	0,00
LIMLP_14205	-1,02	0,00	-1,29	0,00
LIMLP_14280	-1,22	0,00		
LIMLP_14375	1,01	0,00		
LIMLP_14415	1,65	0,00	1,24	0,02
LIMLP_14420	1,27	0,00		
LIMLP_14425	1,08	0,00		
LIMLP_14450	-1,70	0,00		
LIMLP_14455	-1,76	0,00		
LIMLP_14460	-1,86	0,00	-1,21	0,00
LIMLP_14465	2,04	0,00		
LIMLP_14480	1,17	0,00		
LIMLP_14560	1,11	0,00	2,26	0,00
LIMLP_14585	1,03	0,00		
LIMLP_14590	-1,02	0,00	-1,65	0,00
LIMLP_14610	-1,40	0,00		
LIMLP_14615	-1,29	0,00		
LIMLP_14620	-1,21	0,00	-1,24	0,00
LIMLP_14650	1,01	0,00	1,38	0,00
LIMLP_14715	1,10	0,00		

LIMLP_14745	-1,13	0,00		
LIMLP_14970	-1,22	0,00		
LIMLP_14990	-1,32	0,00		
LIMLP_15080	-1,12	0,00		
LIMLP_15090	-1,41	0,00		
LIMLP_15105	3,59	0,00	1,79	0,00
LIMLP_15110	3,61	0,00	1,83	0,00
LIMLP_15115	3,35	0,00	1,98	0,00
LIMLP_15120	2,61	0,00	1,88	0,00
LIMLP_15255	-1,00	0,00		
LIMLP_15260	-1,26	0,00		
LIMLP_15265	-1,11	0,00		
LIMLP_15275	-1,10	0,00		
LIMLP_15300	1,34	0,00		
LIMLP_15315	-1,44	0,00		
LIMLP_15335	-1,55	0,00	-1,53	0,04
LIMLP_15340	-1,07	0,00		
LIMLP_15510	-1,49	0,00		
LIMLP_15525	-1,30	0,00	-1,25	0,00
LIMLP_15535	4,10	0,00		
LIMLP_15540	3,53	0,00		
LIMLP_15620	-1,74	0,00		
LIMLP_15680	1,15	0,00		
LIMLP_15715	-1,83	0,00		
LIMLP_15720	2,05	0,00		
LIMLP_15810	-1,41	0,00		
LIMLP_15825	1,04	0,01		
LIMLP_16015	1,03	0,00	2,47	0,00
LIMLP_16025	1,78	0,00		
LIMLP_16035	-1,82	0,00		
LIMLP_16065	1,30	0,00		
LIMLP_16165	-1,29	0,00		
LIMLP_16170	-1,61	0,00		
LIMLP_16245	1,09	0,00	-1,16	0,01
LIMLP_16265	1,13	0,00	1,40	0,00
LIMLP_16420	-1,37	0,00		
LIMLP_16505	1,00	0,00	1,81	0,00
LIMLP_16520	3,83	0,00		
LIMLP_16525	2,96	0,00		
LIMLP_16645	-1,25	0,00		
LIMLP_16665	1,26	0,00		
LIMLP_16690	-1,23	0,00		
LIMLP_16745	-1,04	0,00		
LIMLP_16765	1,07	0,00	1,64	0,00
LIMLP_16800	1,20	0,00	2,00	0,00
LIMLP_16805	1,29	0,00	1,97	0,00

LIMLP_16810	1,92	0,00	1,91	0,00
LIMLP_16825	1,17	0,00		
LIMLP_16870	1,98	0,00	2,55	0,00
LIMLP_16925	-1,55	0,00		
LIMLP_16930	-1,17	0,00		
LIMLP_16935	-1,08	0,00		
LIMLP_16975	-1,08	0,00		
LIMLP_16990	-1,09	0,00	-1,66	0,00
LIMLP_17060	-1,11	0,00		
LIMLP_17130	-1,15	0,00		
LIMLP_17165	-1,54	0,00		
LIMLP_17200	-1,48	0,00	-1,69	0,00
LIMLP_17230	1,09	0,00	1,14	0,00
LIMLP_17235	1,28	0,00		
LIMLP_17250	-1,02	0,00	1,51	0,00
LIMLP_17305	1,08	0,00		
LIMLP_17420	-1,04	0,00	2,26	0,01
LIMLP_17425	-1,40	0,00		
LIMLP_17430	-1,08	0,00		
LIMLP_17435	-1,23	0,00		
LIMLP_17440	-1,17	0,00		
LIMLP_17445	-1,44	0,00		
LIMLP_17450	-1,16	0,00		
LIMLP_17455	-1,05	0,00		
LIMLP_17460	-1,08	0,00		
LIMLP_17465	-1,81	0,00		
LIMLP_17470	-1,69	0,00		
LIMLP_17475	-1,65	0,00		
LIMLP_17600	-1,73	0,00		
LIMLP_17655	1,02	0,00		
LIMLP_17835	2,92	0,00		
LIMLP_17840	1,20	0,00		
LIMLP_18140	1,22	0,00	1,34	0,00
LIMLP_18145	1,26	0,00		
LIMLP_18245	-1,42	0,00		
LIMLP_18250	-1,47	0,00		
LIMLP_18275	1,44	0,00		
LIMLP_18310	1,18	0,00		
LIMLP_18455	-1,34	0,00		
LIMLP_18460	-2,44	0,00	1,21	0,00
LIMLP_18465	-2,54	0,00	1,41	0,01
LIMLP_18470	-2,18	0,00		
LIMLP_18475	-2,09	0,00		
LIMLP_18480	-2,07	0,00		
LIMLP_18485	-2,10	0,00		
LIMLP_18490	-2,44	0,00		

LIMLP_18495	-2,07	0,00		
LIMLP_18500	-1,93	0,00	1,12	0,02
LIMLP_18505	-1,94	0,00		
LIMLP_18510	-1,92	0,00	1,21	0,00
LIMLP_18515	-1,66	0,00	1,21	0,00
LIMLP_18520	-1,50	0,00		
LIMLP_18525	-1,00	0,00		
LIMLP_18595	1,18	0,00		
LIMLP_18600	1,07	0,00		
LIMLP_18620	2,12	0,00		
LIMLP_18625	2,15	0,00		
LIMLP_18670	-1,03	0,00	-1,53	0,00
LIMLP_18675	-1,37	0,00	-1,07	0,00
LIMLP_18680	-1,91	0,00	-1,16	0,00
LIMLP_18690	-1,09	0,00		
LIMLP_18695	-1,07	0,00	-1,13	0,00
LIMLP_18700	-1,21	0,00	-1,50	0,00
LIMLP_18705	-1,22	0,00	-1,11	0,01
LIMLP_18725	1,14	0,00	-1,59	0,00
LIMLP_18840	1,05	0,00		
LIMLP_18990	-1,04	0,00		
LIMLP_19000	1,39	0,00	1,74	0,00
LIMLP_19070	1,45	0,00		
LIMLP_19075	-1,50	0,00		
LIMLP_19080	-1,13	0,00		
LIMLP_19115	-1,32	0,00		
LIMLP_19165	1,09	0,00		
LIMLP_19180	1,21	0,00		
LIMLP_19195	1,30	0,00	2,67	0,00
LIMLP_19320	-1,37	0,00	-3,28	0,00
LIMLP_19370	1,55	0,00		
LIMLP_19450	1,11	0,00	1,17	0,00
LIMLP_19510	1,03	0,00	1,10	0,00
LIMLP_19545	-1,21	0,00		
LIMLP_19550	-1,09	0,00		
LIMLP_19605	1,09	0,00	1,04	0,02
LIMLP_19610	1,18	0,00	3,04	0,00

Supplementary Table 8: Differentially expressed genes related to virulence

Locus	Gene name	Fold Change	padj
LIMLP_15115	*dnaK*	3,9	3,6,E-04
LIMLP_00490		3,7	1,5,E-05
LIMLP_11105		2,2	2,6,E-05
LIMLP_03820		-1,5	5,0,E-02
LIMLP_07160		-1,8	7,6,E-06
LIMLP_13775	*flaA1*	-2,0	8,4,E-04
LIMLP_04845		-2,0	5,5,E-03
LIMLP_13750	*orfC*	-2,0	4,0,E-02
LIMLP_11985	*glnA*	-2,0	1,6,E-02
LIMLP_00285	*rlpA*	-2,2	1,4,E-06
LIMLP_13280		-2,2	4,6,E-04
LIMLP_18410	*rsbU*	-2,2	2,6,E-02
LIMLP_06600	*lipL*	-2,4	2,1,E-03
LIMLP_00265		-2,6	6,3,E-10
LIMLP_11235		-2,8	2,3,E-05
LIMLP_16990		-3,2	2,8,E-05
LIMLP_10145	*cat*	Not significantly modulated (NS)	
LIMLP_15405		NS	
LIMLP_15415		NS	
LIMLP_03070	*tufB*	NS	
LIMLP_06640		NS	
LIMLP_05255		NS	
LIMLP_09705		NS	
LIMLP_04780		NS	
LIMLP_02010		NS	
LIMLP_08500		NS	
LIMLP_12950		NS	
LIMLP_16665		NS	
LIMLP_09740	*eno*	NS	
LIMLP_15190		NS	
LIMLP_05865		NS	
LIMLP_15185		NS	
LIMLP_04025		NS	
LIMLP_03665		NS	
LIMLP_17395		NS	
LIMLP_11665	*ttg2D*	NS	
LIMLP_07685		NS	
LIMLP_09115		NS	
LIMLP_11180		NS	
LIMLP_14510		NS	
LIMLP_14055		NS	
LIMLP_02125		NS	
LIMLP_09235		NS	

LIMLP_04660		NS
LIMLP_15240		NS
LIMLP_16490		NS
LIMLP_03045		NS
LIMLP_03370		NS
LIMLP_05790	*citE*	NS
LIMLP_03480	*paaJ*	NS
LIMLP_04390		NS
LIMLP_13605		NS
LIMLP_17720		NS

Supplementary Table 9: Comparison with *L.biflexa* biofilm.

The orthology was retrieved from MaGe: minLrap ≥ 0.8 ; maxLrap ≥ 0 ; Identity ≥ 35%

	L. biflexa			*L. interrogans* Biofilm d21 vs Planktonic cells d5			Comparison	
Condition	UP	DOWN	Total	UP	DOWN	Total	Upregulated in both conditions	Downregulated in both conditions
BvsP_48h	73	140	213	10	23	33	1	8
BvsP_120h	82	60	142	5	10	15	2	6
B_120hvs48h	101	116	217	4	19	23	2	17
P_120h_vs48h	180	191	371	21	37	58	9	29

Supplementary Table 10: List of genes removed from the analysis

Locus	Locus	log2FC	padj
LIMLP_RS15465	LIMLP_15505	-0,67	4,9,E-02
LIMLP_RS15980	LIMLP_16020	1,03	1,4,E-04
LIMLP_RS20030	rnpA	-0,64	5,0,E-02
LIMLP_RS20045		1,10	2,2,E-02
LIMLP_RS20140		1,67	1,3,E-02
LIMLP_RS20165		1,77	3,6,E-02
LIMLP_RS20330		-0,80	2,7,E-02
LIMLP_RS21990		1,13	2,3,E-02
LIMLP_RS22115		2,48	5,4,E-08
LIMLP_RS22330		1,87	6,1,E-06
LIMLP_RS22515		-0,92	3,5,E-02
LIMLP_RS22620		1,23	3,2,E-02
LIMLP_RS23135		1,46	3,6,E-03
LIMLP_RS23210		-0,82	1,0,E-02
LIMLP_RS23365		3,33	2,6,E-02
LIMLP_RS23430		-1,79	7,1,E-11
LIMLP_RS23480		1,64	4,0,E-02
LIMLP_RS23525		1,62	1,3,E-06
LIMLP_RS23575		0,92	1,4,E-02
LIMLP_RS13715	LIMLP_13745	-1,69	3,3,E-04

Supplementary Table 11: List of the primers used for RNA-seq validation

Primer name	Sequences (5' to 3')
LIMLP_02515_F	ACGAGGAATTCAAGGGATGATTGA
LIMLP_02515_R	GCAATCCCCTAGATACGCCG
LIMLP_04310_F	GGAGGGTTCTCAGGCAAAGA
LIMLP_04310_R	TCACCGGAACGAATTCCCAA
LIMLP_13670_F	AGGAGTCGGTCCAATGCAAG
LIMLP_13670_R	GATCGACAAATCCTTGCCGC
LIMLP_19325_F	ATCACAGAATCCGAGTTAGACGA
LIMLP_19325_R	CCTTACGGAAACCGGTGGATT
LIMLP_14975_F	TGGATCGGACAAGGTCCTAGA
LIMLP_14975_R	TCGTTTTACTCCACCGGGTT
16Slept-outF	GGCGGCGCGTCTTAAACATG
16Slept-inR2	CTTAACCTGCTGCCTCCCGTA
